# Characterization of the transcriptome of fast and slow muscle myotomal fibres in the pacu (*Piaractus mesopotamicus*)

**DOI:** 10.1186/s12864-015-1423-6

**Published:** 2015-03-14

**Authors:** Edson A Mareco, Daniel Garcia de la Serrana, Ian A Johnston, Maeli Dal-Pai-Silva

**Affiliations:** Institute of Biosciences of Botucatu, São Paulo State University - UNESP, Botucatu, 18618-970 São Paulo Brazil; School of Biology, Scottish Oceans Institute, University of St Andrews, St Andrews, KY16 8LB Scotland, UK

**Keywords:** Paralogues, Ubiquitin-specific proteases (USP), Aquaculture genomics

## Abstract

**Background:**

The Pacu (*Piaractus mesopotamicus*) is a member of the Characiform family native to the Prata Basin (South America) and a target for the aquaculture industry. A limitation for the development of a selective breeding program for this species is a lack of available genetic information. The primary objectives of the present study were 1) to increase the genetic resources available for the species, 2) to exploit the anatomical separation of myotomal fibres types to compare the transcriptomes of slow and fast muscle phenotypes and 3) to systematically investigate the expression of Ubiquitin Specific Protease (USP) family members in fast and slow muscle in response to fasting and refeeding.

**Results:**

We generated 0.6 Tb of pair-end reads from slow and fast skeletal muscle libraries. Over 665 million reads were assembled into 504,065 contigs with an average length of 1,334 bp and N50 = 2,772 bp. We successfully annotated nearly 47% of the transcriptome and identified around 15,000 unique genes and over 8000 complete coding sequences. 319 KEGG metabolic pathways were also annotated and 380 putative microsatellites were identified. 956 and 604 genes were differentially expressed between slow and fast skeletal muscle, respectively. 442 paralogues pairs arising from the teleost-specific whole genome duplication were identified, with the majority showing different expression patterns between fibres types (301 in slow and 245 in fast skeletal muscle). 45 members of the USP family were identified in the transcriptome. Transcript levels were quantified by qPCR in a separate fasting and refeeding experiment. USP genes in fast muscle showed a similar transient increase in expression with fasting as the better characterized E3 ubiquitin ligases.

**Conclusion:**

We have generated a 53-fold coverage transcriptome for fast and slow myotomal muscle in the pacu (*Piaractus mesopotamicus*) significantly increasing the genetic resources available for this important aquaculture species. We describe significant differences in gene expression between muscle fibre types for fundamental components of general metabolism, the Pi3k/Akt/mTor network and myogenesis, including detailed analysis of paralogue expression. We also provide a comprehensive description of USP family member expression between muscle fibre types and with changing nutritional status.

**Electronic supplementary material:**

The online version of this article (doi:10.1186/s12864-015-1423-6) contains supplementary material, which is available to authorized users.

## Background

Fish myotomes are composed of anatomically segregated muscle fibre types each with distinct contractile and metabolic phenotypes [1]. Based on their contractile speed skeletal muscle fibres are classified as slow, intermediate or fast [2,3]. Fast twitch (white) muscle fibres comprise the bulk of the myotome and are recruited for energetic movements associated with prey capture and escape behaviour [4]. Fast fibres have elevated densities of myofibrils, reduced myoglobin content and higher a capillary density than slow fibres and utilise phosphagen breakdown and anaerobic glycolysis to power contraction [5]. Sustained swimming activity is supported by superficial layers of slow (red) and intermediate (pink) twitch fibres which are recruited at slow and high cruising speeds respectively [3]. Slow fibres have extensive lipid and glycogen stores, abundant mitochondria and high capillary densities reflecting their reliance on aerobic metabolism [5]. Intermediate (pink) muscle fibre types are found between the slow and fast fibre layers, but express distinct isoforms of myosin heavy chains [6] and have intermediate contraction speeds and metabolic characteristics [3,7].

Muscle growth reflects the balance between protein synthesis and degradation. These two processes are influenced by numerous biotic and abiotic factors including food availability, growth factors, age, sex, diet composition, swimming activity, oxygen saturation, light and temperature [8,9]. The Insulin-like growth factor (Igf) network, composed of Igfs, binding proteins (Igfbp) and receptors (Igf1r and Igf2r), plays a pivotal role in integrating internal and external inputs to regulate muscle mass [8]. Igf1 regulates several signalling pathways including the Pi3k/Akt/mTor network that controls protein synthesis [8,9]. Typically, fibre production continues until 45% of the maximum body length of the fish, and subsequent growth is entirely by fibre elongation and hypertrophy [10-12]. Myogenesis involves the activation, proliferation and fusion of a resident myoblast population involving hundreds of structural and regulatory genes [10,11].

Muscle protein degradation occurs through three major pathways [13,14] namely: membrane-bound lysosomal enzymes, calpain proteases [14,15], and the Ubiquitin Proteasome (Ub) Pathway (UPP) [16]. UPP represents the most important system for degradation of unnecessary or damaged proteins. Targeted proteins are linked to ubiquitin, which acts as a recognition tag for the proteasome. Ubiquitin tagging of proteins requires the coordinated activity of three classes of enzymes known as E1, E2 and E3. It is the E3 enzymes, also known as E3-ubiquitin ligases, which conjugate ubiquitin to the target protein [17]. Ubiquitin mediated degradation can be reversed through action of deubiquitinating enzymes (DUB), a large group of proteases that cleave ubiquitin-protein conjugates removing the UPP signal, and play an essential role in the regulation of protein degradation [18]. DUBs are subdivided into four families: ubiquitin C-terminal hydrolases (UCHs), ovarian tumour proteases (OTUs), JAB1/MPN/MOV34 metalloenzymes and ubiquitin-specific proteases (USPs) [18]. USP is the largest family of DUB and regulates a wide variety of cellular processes. Although the essential role of USPs in protein degradation is well established, less is known about the function and regulation of specific family members: for example Usp7 has been associated with p53 and Akt turnover, Usp8 with receptor tyrosine endocytosis, Usp33 with the Von Hippel–Lindau disease (VHL) pathway and Usp19 is thought to have a role in muscle development [19]. There have been several studies of the expression of protein degradation related genes in fish, mostly in response to varying nutrition and focused on E3-ubiquitin ligases (particularly Fbox32 and Murf1) [20,21]. In contrast, nothing is known about the transcriptional regulation of USP family members.

The pacu (*Piaractus mesopotamicus*) is a member of the characiform family (SuperOder Ostariophysi) native to the Prata Basin (South America) and is a target for finfish aquaculture in Brazil. One of the main limitations for the development of a selective breeding program for this species is a lack of genetic information as well as limited knowledge about its physiology. Next Generation Sequencing (NGS) technologies have dramatically increased the amount of sequence data for teleosts and helped to overcome the lack of annotated genomes (so far only 12 fish annotated genomes are publicly available) [22].

The primary objectives of the present study were 1) to increase the genetic resources available for pacu 2) to exploit the anatomical separation of fibre types to characterise the expression signatures of fast and slow muscles and 3) to systematically investigate the expression of USPs in fast and slow muscle in response to fasting and refeeding. Teleost fish underwent a teleost-specific genome duplication (TSGD) event around 450 million years ago (Mya) which was followed by diploidisation and gene loss [23,24]. It is thought that around 15 to 20% of TSGD paralogues have been retained in the diploid genome of extant species [23-26]. Several studies have demonstrated that paralogues from the TSGD or the salmonid-specific whole-genome duplication (WGD), which occurred 88Mya [27], can display different patterns of expression during myogenesis and muscle growth [27-29]. A secondary objective was therefore to search for evidence of differential expression between teleost specific paralogues within and between muscle fibre types.

## Results and discussion

### De novo assembly

Individual barcoded libraries from fast and slow skeletal muscle tissues were generated from 5 adult pacu (1.5 ± 0.61 kg; mean ± SD) and sequenced using HiSeq 2000 platform yielding a total of 374,952,267 and 390,706,230 pair-end reads respectively (~0.6 Tb) (raw reads were deposited in the European Nucleotide Archive accession number PRJEB6656). 665,042,497 trimmed paired-end reads (86% of the total) were successfully assembled into 504,065 contigs with an average length of 1,334 bp and N50 = 2,772 bp (Table 1). The present study significantly improves upon previous teleost transcriptomes [30]. A total of 237,637 contigs (47%) were successfully annotated, representing over 15,000 unique genes with an average coverage of 53 and over 8,000 genes with >90% of the coding sequence (CDS) represented (Table 1; annotation results can be found in Additional file 1). The number of genes found represent between 56-65% of all protein-coding genes currently identified in *Danio rerio* (Cypriniformes) and *Astyanax mexicanus* (Characiformes) the only two other Ostariophysi genomes available [22]. Pacu contigs were also annotated and classified into 319 different vertebrate signalling and metabolic pathways (Additional file 2). In some cases, such as for the Pi3k/Akt/mTor network we found that over 90% of the genes were represented.

**Table 1:** **Pacu** ***de novo*** **transcriptome metrics**

**Parameters**	**Slow skeletal muscle**	**Fast skeletal muscle**
Reads	704,550,985	765,658,497
Reads assembled	521,568,496	665,042,497
Contigs	504,065	
Contig mean lenght (bp)	1,334	
N50 (bp)	2,772	
Contigs Annotated	232,637	
Unique genes	15,000	
Paralogues	406	
Differentially expressed genes	3,473	2,458
Annotated Pathway	314	

N50: The value was computed by sorting all contigs from largest to smallest and by determining the minimum set of contigs whose sizes total 50% of the entire transcriptome.

**Figure 1:**
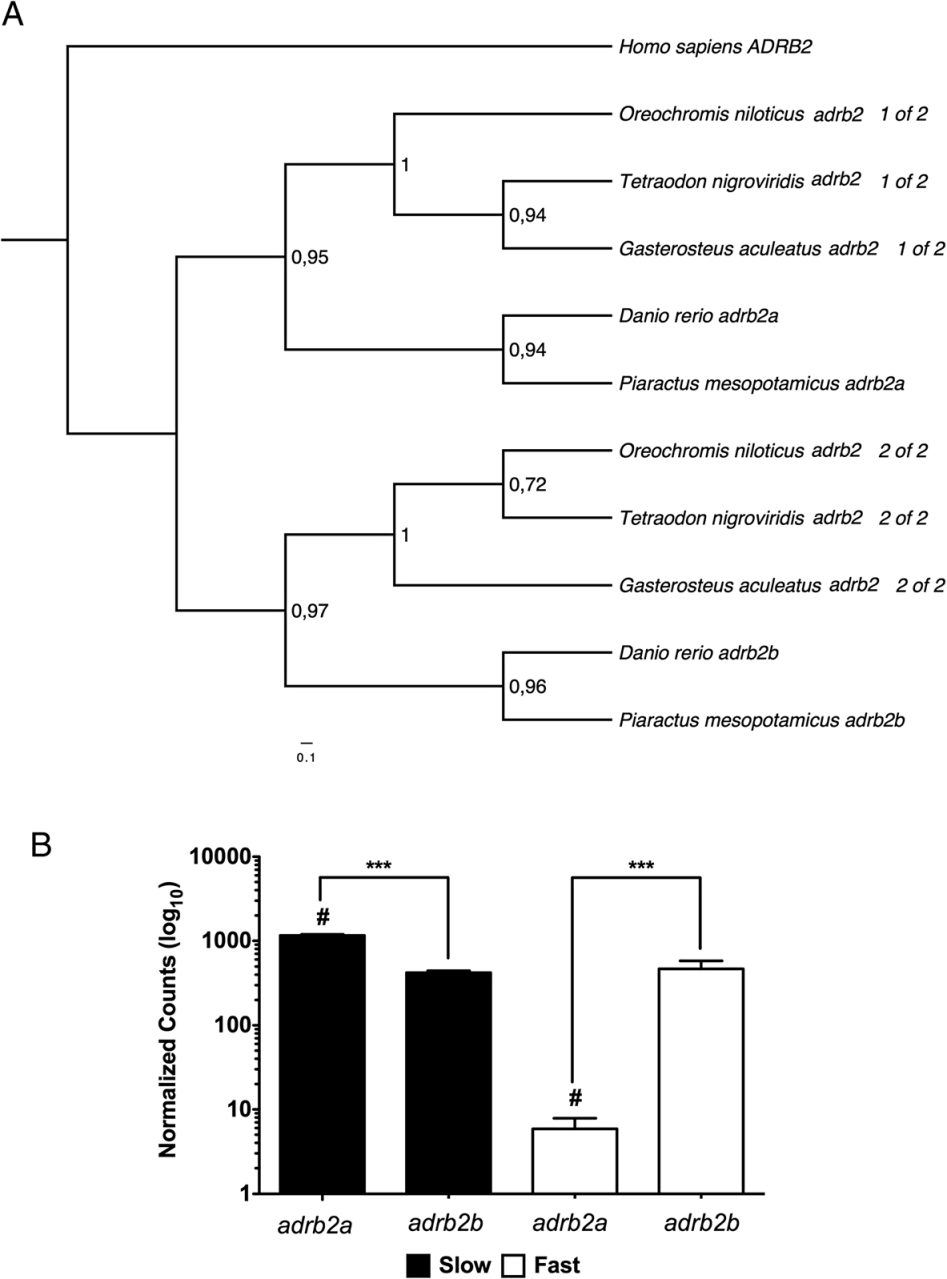
**Example of a teleost-specific paralogue phylogenetic and digital expression analysis in pacu fast and slow skeletal muscle. (A)** Phylogenetic analysis of the *adrb2* teleost-specific paralogues. Human *ADRB2* was used as an out-group. **(B)** *adrb2a* and *adrb2b* digital gene expression. Digital gene expression is represented using a logarithmic scale for slow (full bars) and fast (empty bars) skeletal muscle. Significant differences for paralogues within (***; FDR < 0.001) and between (#; FDR < 0.05) fibre types are indicated. Values represent mean ± SE (n = 5). *Adrb2*: *beta-2 adrenergic receptor.*

The current transcriptome dramatically increases the genetic resources available for the future development of genetic-based breeding programmes in pacu. Simple sequence repeats (SSRs) or microsatellites are widely used for parentage identification and stock management in family selection programs [31]. Screening for SSRs was focused on those contigs for which over 90% of the CDS was present to assure that repeated sequences were correctly identified in the UTR regions. A total of 380 SSRs were identified in the transcriptome, and the great majority of them (54%) were repeats of a dinucleotide motif (Table 2; Additional file 3) increasing the number of potential microsatellites for this species [26]. We also screened the annotated transcriptome to identify TSGD-paralogues. It has been estimated that around 15 to 20% of teleost-specific paralogues have been retained in the diploid genomes of extant species [23-28] with a slightly higher proportion retained in Ostariphysi genomes [26]. We identified a total of 442 paralogues pairs in the pacu when compare with zebrafish (884 genes; Figure 1A, Additional file 4). Although this is a significant improvement when compared with previous studies [22] the number of paralogues identified was still lower than what we could expect from a 16,000 gene transcriptome.

**Table 2:** **Microsatellite identification**

**Motif**	**Count**	**%**
AAC	1	0,3
AC	113	36,7
ACAG	1	0,3
ACCT	1	0,3
AG	107	34,7
AGCG	1	0,3
AGGC	1	0,3
AT	28	9,1
ATC	1	0,3
C	52	16,9
CG	2	0,6

### Digital gene expression analysis

Individual fast and slow muscle libraries were mapped to the complete transcriptome. Mapped reads were normalized by contig length, library size and only those with an average of more than 10 mapped were used to estimate digital gene expression (DGE). Whereas there are several transcriptomic studies in teleosts [29,32,33] to our knowledge this is the first one to compare global patterns of DGE in muscle different fibre types. Global DGE analysis revealed 956 and 604 genes differentially expressed between slow and fast skeletal muscle respectively (Additional file 5). Gene ontology (GO) enrichment analysis of the differently expressed genes (Table 3; Additional file 6) revealed a significant enrichment in genes involved in oxidative phosphorylation and lipid metabolism (GO:0005739; 0006629; 0009055; 0005811; 0018925; Table 3) in slow skeletal muscle and carbohydrate metabolism and kinase activity (GO:0005975; 0019752; 0016301) in fast skeletal muscle. These results are in agreement with the main differences described between slow and fast muscle with respect to their predominant means of energy supply [7]. To further validate GO analysis results, reads mapped were individually explored in all components related to general metabolic pathways present in our transcriptome: glycolysis, ß-oxidation, Krebs cycle and the electron transport chain (Additional file 7). The majority of glycolysis-related enzymes found, such as *glucose-6-phosphate isomerase* (*gpi*), *6-phosphofructokinase* (*pfkm*) or *enolase* (*eno*), were significantly more highly expressed in fast skeletal muscle (FDR < 0.05) (Additional file 7). As expected slow muscle had a significantly higher DGE in almost all components of the Krebs cycle, ß-oxidation pathway and the electron transport chain (Additional file 8). Therefore DGE results captured fundamental physiological and metabolic differences between tissues. We also studied DGE in relation to the Pi3k/mTor pathway, myogenesis-related genes and TSGD-paralogues between fibre types. Previous studies have reported that TSGD-paralogues can display different expression profiles in muscle during myogenesis and in response to varying nutrition, however those studies were always restricted to fast skeletal muscle and to a limited number of paralogues [34,35]. By using DGE we found that 301 and 245 TSGD-paralogues pairs were differentially expressed for slow and fast skeletal muscle respectively (FDR < 0.05; example in Figure 1B; Additional file 9). Similarly, we found 124 paralogues with significant differences in DGE between muscle types (FDR < 0.05; Figure 1B). Various mechanisms have been postulated to explain TSGD paralogue retention during evolution including mutations leading to differential regulation in expression (sub-functionalization) and/or the evolution of some novel function (neofunctionalization) [36]. Whilst we cannot distinguish between these possibilities in the present study it is apparent that TSGD paralogue retention has significantly contributed to phenotypic patterning of gene expression between fast and slow muscle fibre types.

**Table 3:** **Gene ontology (GO) enrichment analysis**

	**GO Term**	**Name**	**Type**	**FDR**	**Genes in category**
Slow muscle	GO:0003779	Actin binding	F	2,30E-31	174
	GO:0005739	Mitochondrion	C	1,20E-30	305
	GO:0006811	Ion transport	P	6,20E-28	209
	GO:0006091	Generation of precursor metabolites and energy	P	4,00E-21	125
	GO:0003774	Motor activity	F	9,10E-21	79
	GO:0030154	Cell differentiation	P	2,70E-14	398
	GO:0009653	Anatomical structure morphogenesis	P	3,50E-14	397
	GO:0005198	Structural molecule activity	F	6,10E-11	126
	GO:0007275	Multicellular organismal development	P	9,30E-09	561
	GO:0005509	Calcium ion binding	F	9,30E-09	107
	GO:0005216	Ion channel activity	F	9,60E-09	63
	GO:0006629	Lipid metabolic process	P	2,50E-08	153
	GO:0009055	Electron carrier activity	F	8,40E-08	39
	GO:0007010	Cytoskeleton organization	P	4,50E-07	181
	GO:0005886	Plasma membrane	C	8,80E-07	420
	GO:0005783	Endoplasmic reticulum	C	4,80E-06	149
	GO:0005615	Extracellular space	C	2,00E-04	61
	GO:0000166	Nucleotide binding	F	2,50E-04	397
	GO:0019725	Cellular homeostasis	P	3,60E-04	93
	GO:0007267	Cell-cell signaling	P	4,00E-04	105
	GO:0005811	Lipid particle	C	2,10E-03	13
	GO:0019825	Oxygen binding	F	3,20E-03	5
	GO:0005975	Carbohydrate metabolic process	P	3,50E-03	99
Fast muscle	GO:0006091	Generation of precursor metabolites and energy	P	7,50E-81	194
	GO:0005975	Carbohydrate metabolic process	P	1,00E-71	225
	GO:0005783	Endoplasmic reticulum	C	4,90E-23	174
	GO:1901564	Organonitrogen compound metabolic process	P	4,90E-23	115
	GO:0019752	Carboxylic acid metabolic process	P	4,90E-23	115
	GO:0005216	Ion channel activity	F	4,90E-23	80
	GO:0005509	Calcium ion binding	F	7,20E-23	122
	GO:0006811	Ion transport	P	4,90E-15	140
	GO:0016301	Kinase activity	F	8,60E-15	188
	GO:0019725	Cellular homeostasis	P	3,50E-14	107
	GO:0003779	Actin binding	F	5,50E-12	101
	GO:0005829	Cytosol	C	1,20E-08	263
	GO:0005886	Plasma membrane	C	1,60E-07	331
	GO:0005856	Cytoskeleton	C	7,60E-06	223
	GO:0043234	Protein complex	C	1,20E-04	321
	GO:0009056	Catabolic process	P	3,10E-04	244

**Figure 2:**
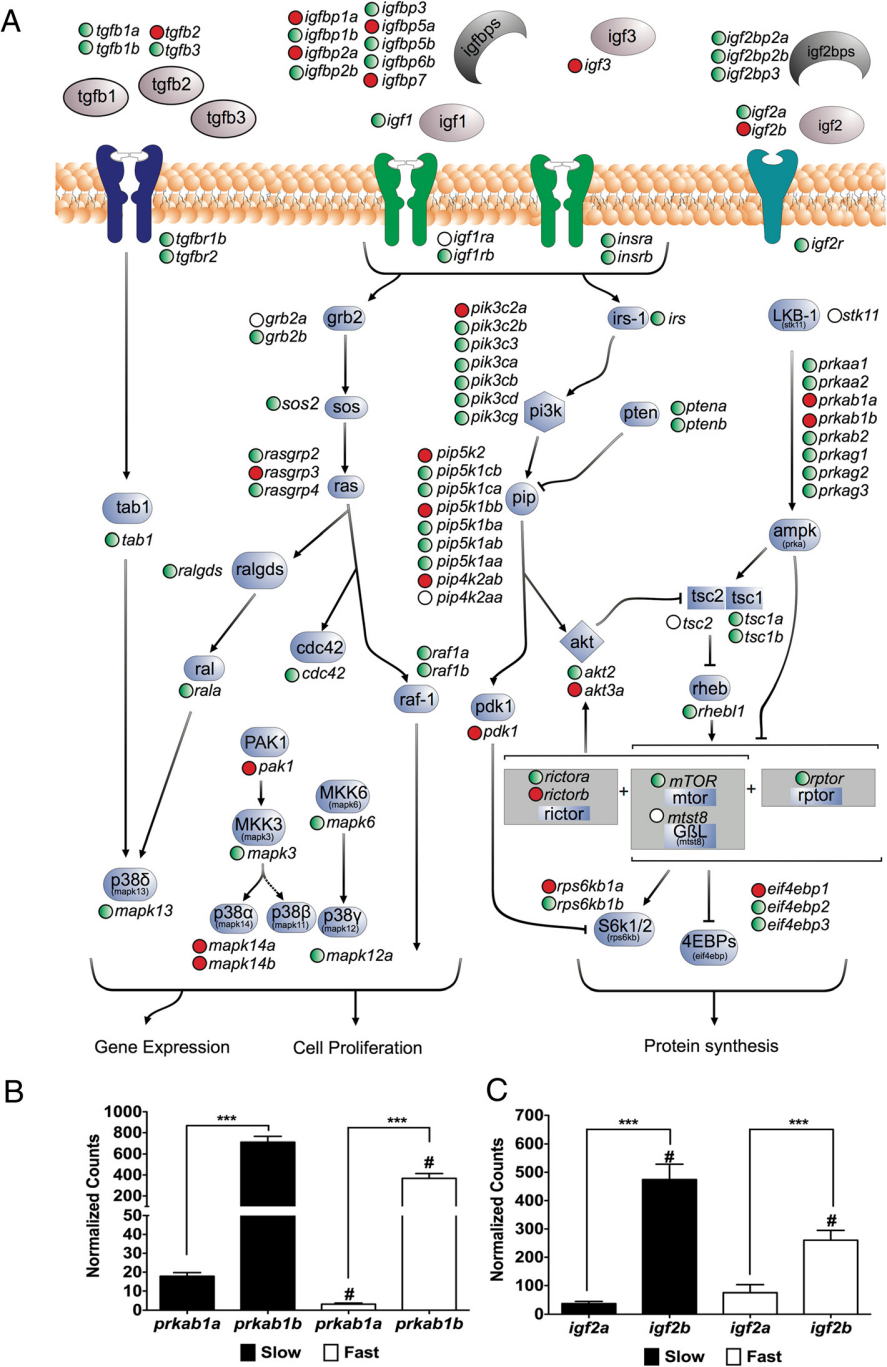
**Digital gene expression of the Pi3k/mTor pathway components in pacu fast and slow skeletal muscle. (A)** Pi3k/mTor components represented in the transcriptome mapped into a reconstruction of the same pathway. “Red circles” and “Empty circles” indicate components significantly higher in slow and fast skeletal muscle respectively muscle (FDR < 0.05). “Green circles” indicate components with no significant differences between fibre types. **(B)** *igf2a* and *igf2b* digital gene expression. **(C)** *prkab1a* and *prkab1b* digital gene expression. Digital gene expression is represented using a logarithmic scale for slow (full bars) and fast (empty bars) skeletal muscle. Significant differences for paralogues within (***; FDR < 0.001) and between (#; FDR < 0.05) fibre types are indicated. Values represent mean ± SE (n = 5). *Igf2: insulin-like growth factor 2; Prkab1: 5’ AMP-activated protein kinase subunit beta 1*.

The Pi3k/Akt/mTor network is involved in several cellular processes: muscle growth, cell cycle, muscle differentiation and myoblast proliferation [8,37,38]. The majority of the pathway’s components were identified in the transcriptome, including several paralogues (Figure 2; Additional file 10). We found differences in DGE between muscle types for 28 components (FDR < 0.05; Figure 2). Only 6 genes were more abundant in fast skeletal muscle including *insulin-like growth factor receptor 1a* (*igfr1a*) and *tuberous sclerosis 2* (*tsc2*) (Figure 2, empty circles). A total of 22 components of the pathway were found to be more highly expressed in slow skeletal muscle including *insulin-like growth factor 2b* (*igf2b*), several insulin-like growth factors binding proteins (*igfbp1a*, *igfbp2a*, *igfbp5a* and *igfbp7*), *protein kinase B gamma* (*akt3*), *ribosomal protein S6 kinase beta 1a* (*rps6kb1a*), *eukaryotic translation initiation factor 4E-binding protein 1* (*eif4ebp1*) and mitogen-activated protein kinases (*mapk14a* and *mapk14b*) (Figure 2, red circles). Those genes significantly up regulated in slow skeletal muscle are directly involved in the stimulation of protein synthesis, suggesting a higher protein synthesis potential in this fibre type [39,40]. Some of the genes significantly up regulated in fast skeletal muscle are associated with the regulation of protein synthesis during fasting. For instance, *lkb1* is expressed when energy levels are low, *mlst8* binds *mTor* to stabilize the complex when amino acids are not optimal and *tsc2* is related with protein synthesis inhibition [40].

**Figure 3:**
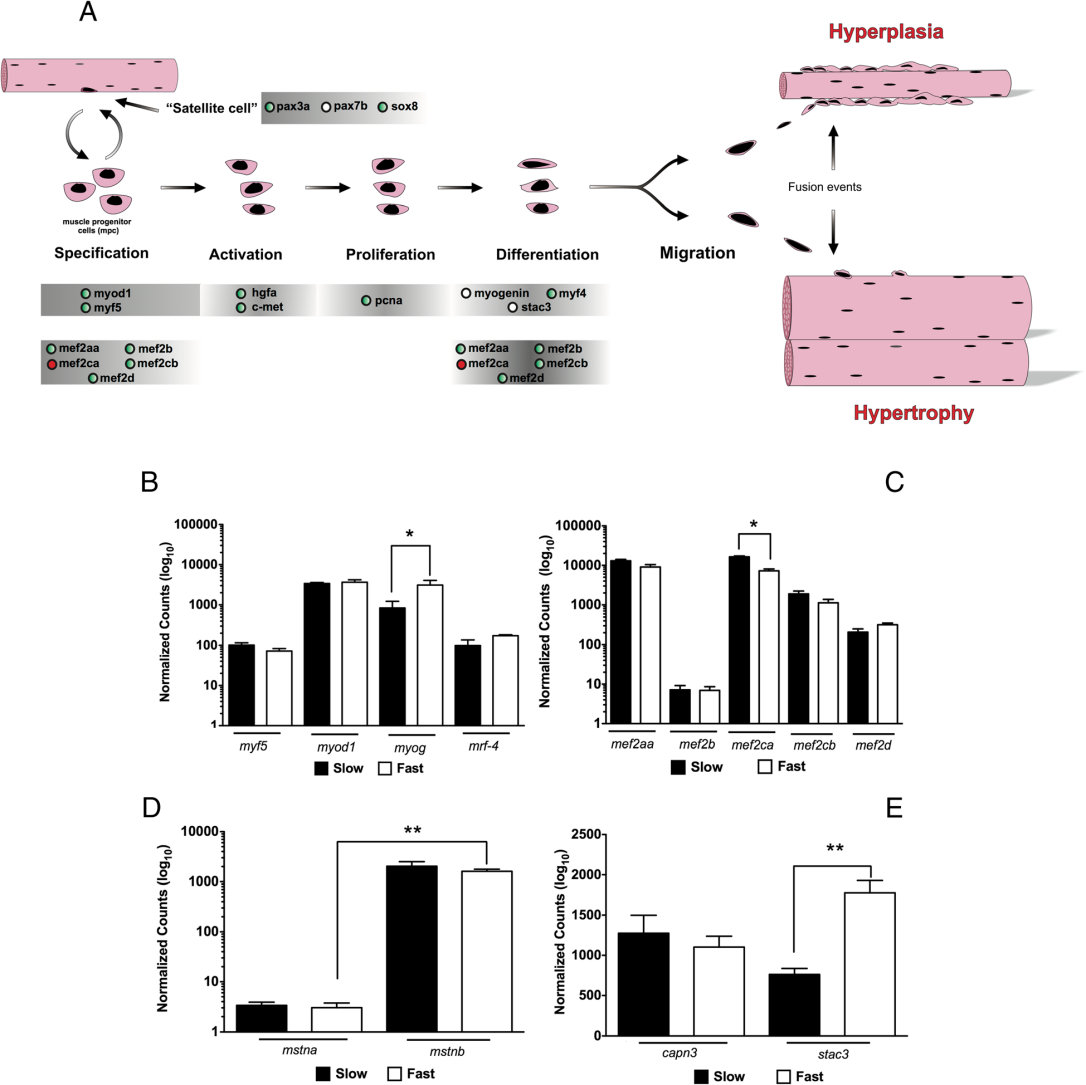
**Digital gene expression of myogenic related genes in pacu fast and slow skeletal muscle. (A)** Myogenic related genes represented in the transcriptome mapped into a schematic reconstruction of the myogenesis process. “Red circles” and “Empty circles” indicate components significantly higher in slow and fast skeletal muscle respectively muscle (FDR < 0.05). “Green circles” indicate components with no significant differences between fibre types. **(B)** Myogenic regulatory factors (MRFs) digital gene expression. **(C)** Myocyte-specific enhancer factor 2 genes digital gene expression. **(D)** Myostatin digital gene expression. **(E)** Calpain 3 and cysteine rich domain 3 digital gene expression. Digital gene expression is represented using a logarithmic scale for slow (full bars) and fast (empty bars) skeletal muscle. Significant differences for paralogues within (***; FDR < 0.001) and between (#; FDR < 0.05) fibre types are indicated. Values represent mean ± SE (n = 5). *Myod1*: *myoblast determination protein 1*; *myf5*: *myogenic factor 5*; *mrf4*: *myogenic regulatory factor 4*; *myog*: myogenin; *mef2*: *myocyte specific enhancer factor 2*; *capn3*: *calpain-3*; *stac3*: *cysteine rich domain 3*; *mstn*: *myostatin*.

DGE was also analysed for the genes associated with myoblast activation, proliferation, differentiation and migration (Figure 3A). *Stac3* is a multifunctional signal adaptor protein which shows a strong correlation with myogenin expression both in cell culture and *in vivo* and is required for normal myotube formation and sarcomere assembly [41]. *Stac3* localises to T-tubules and also functions in the mediation of voltage-induced Ca^2+^ release and contractility [42]. The higher expression of *stac3*, in correlation with *myogenin*, in fast than slow muscle (Figure 3E) may therefore reflect the more extensive development of T-tubules and sarcoplasmic reticulum in this fibre type which are required to achieve shorter contraction cycles during high speed swimming [43]. In contrast, the other myogenic regulatory factors (Figure 3B) and members of the myocyte enhancer gene family (except *mef2ca*) (Figure 3C) had similar expression patterns in fast and slow muscles. Differences in expression for myostatin paralogues has been previously reported in other fish species including rainbow trout (*Oncorhynchus mykiss*) and tilapia (*Oreochromis niloticus*) with *mstna* expression (also known as *mstn2*) confined to brain, testes and spleen and *mstnb* (also known as *mstn1*) more expressed in heart and muscle [44,45].

### Transcriptional regulation of USP and E3-ubiquitin ligases

**Figure 4:**
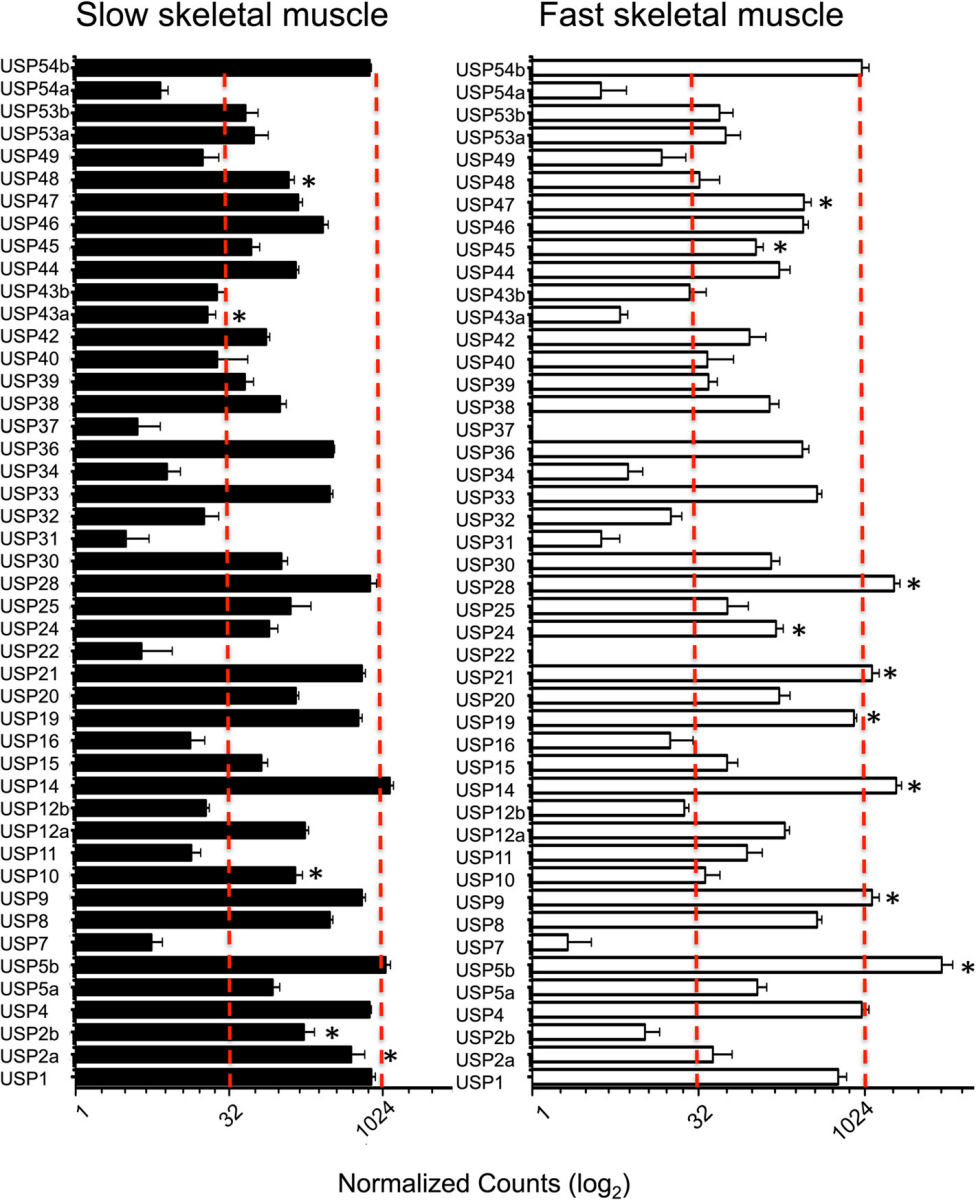
**USP digital gene expression in fast and slow skeletal muscle.** Digital gene expression for all USPs is represented using a 2-logarithmic scale for slow (full bars) and fast (empty bars) skeletal muscle. Significant differences and between fibre types (FDR < 0.05) are indicated (*). Values represent mean ± SE (n = 5).

**Figure 5:**
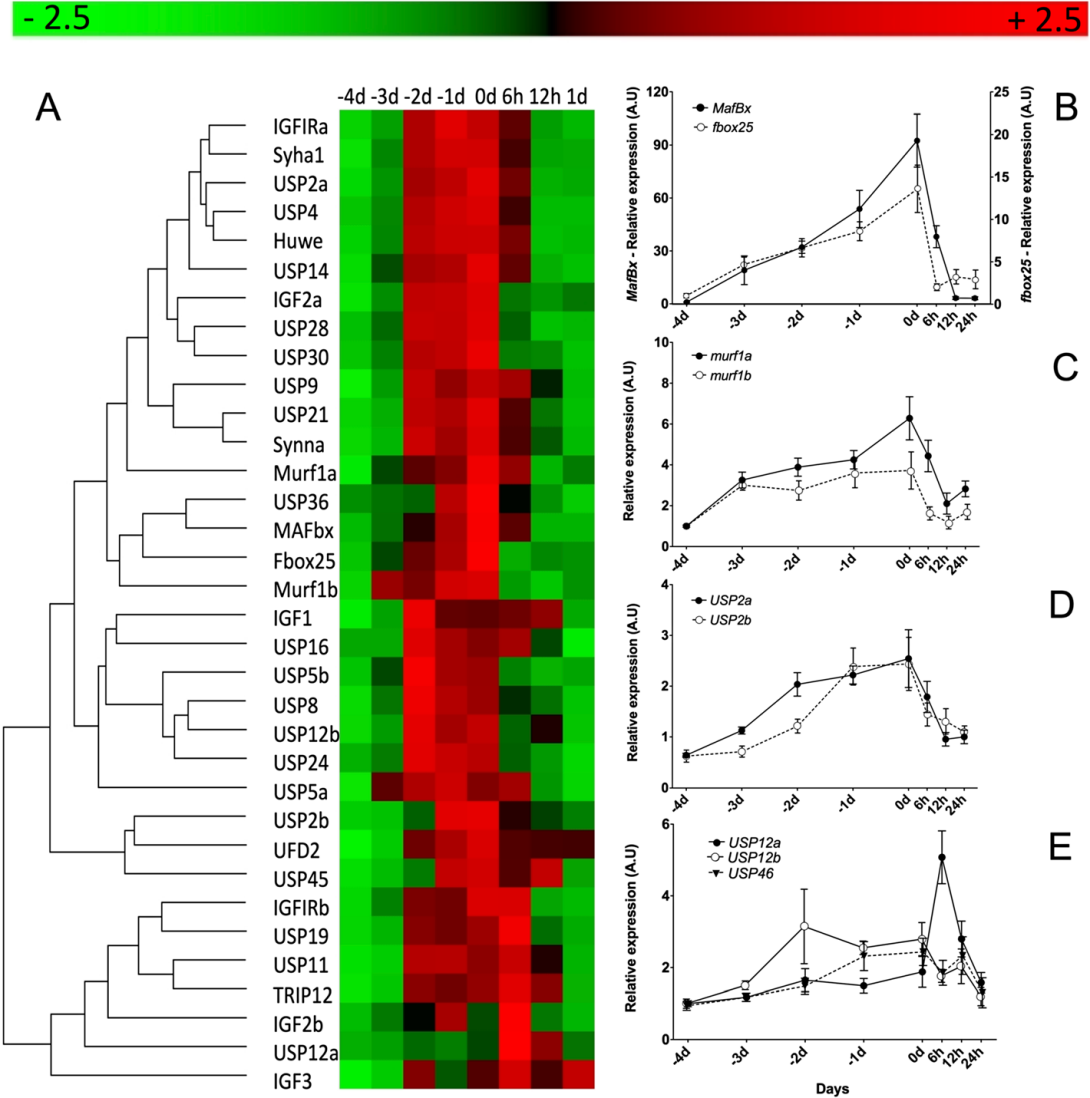
**Relative gene expression in response to fasting and satiation feeding in pacu juveniles in fast skeletal muscle. (A)** Heat map summary and hierarchical cluster for all genes analysed in fast skeletal muscle during transition from 4 days of fasting fasting (−4, −3, −2 and −1) to 24 h of satiation feeding (0, 6, 12 and 24 hours). Rows are standardized to have a mean of 0 and standard deviation of 1. Red indicates high and green low expression values. Relative gene expression graphs for *mafbx* (full circles) and *fbox25* (empty circles) **(B)** *murf1a* (full circles) and *murf1b* (empty circles) **(C)** *usp2a* (full circles) and *usp2b* (empty circles) **(D)** *usp12a* (full circles), *usp12b* (empty circles) and *usp46* (inverted triangles) **(E)**. Values represents mean ± SE (n = 8). *Usp*: *ubiquiting specific peptidase*; *fbox*: *f-box only protein*; *mafbx*: *f-box only protein 32*; *murf1*: *E3-ubiquitin ligase TRIM63*.

The quality of the present transcriptome allowed us to study the regulation of the Ubiquitin Specific Proteases (USP) family, a group of DUBs for which very little is known in skeletal muscle [18]. USPs are a highly diverse family with a common Ubiquitin carboxyl-terminal hydrolase (UCH 2_3) functional domain. Several USPs members contain a variety of other functional domains including dual specific phosphatases (DUSP), zinc-finger in ubiquitin carboxyl-terminal hydrolase (ZF-UBP), ubiquitin associated domains (UBA) or even an Hsp90 binding motif (MEEVD) (Additional file 11). A total of 45 USPs, representing almost the complete repertoire identified zebrafish, were found in the pacu transcriptome. Again, the normalized number of reads mapped was used to estimate their abundance in fast and slow skeletal muscle (Figure 4). We found that 4 USPs were significantly more abundant in slow (*usp2b, usp10, usp43a usp48*) (Figure 4A) and 9 USPs were more highly expressed in fast skeletal muscle (*usp5b, usp9, usp14, usp19, usp21, usp24, usp28, usp45, usp47*) (Figure 4B). The lack of studies concerning USP function in fish makes it difficult to explain these differences in a physiological context. With the objective of gaining knowledge about the role of USPs in muscle growth we performed a nutritional challenge experiment to analyse their expression during the transition from a catabolic to an anabolic state. To this end pacu juveniles were fasted for 4 days followed by a short period of satiation feeding (24 h) to stimulate protein synthesis and a subset of muscle expressed USP genes were measured by real-time PCR (GE) (Figure 4). The sub-set was chosen to reflect their diversity with respect to functional domains present (*usp2a, usp2b, usp4, usp5a, usp5b, usp8, usp9, usp11, usp12a, usp12b, usp14, usp16, usp19, usp21, usp28, usp30, usp36* and *usp46*). The expression of several Pi3k/Akt pathway components (*igf1, igf2a, igf2b, igf1ra, igf1rb* and *igf3*) and E3-ubiquiting ligases (*mafbx, murf1a, murf1b, fbox-25, huwe, ufd2, trip12, syyna* and *syah*) were also measured to gain a better understanding of the metabolic context in which USP expression was occurring (Figure 5A). Transcript abundance from the transcriptome was highly correlated with that determined by qPCR (R^2^ = 0.76; n = 21; P < 0.0001; data not show).

The E3-ubiquitin ligases are an essential part of the proteasome system, directly involved in protein degradation [45,46]. All E3-ubiquitin ligases increased their relative abundance during fasting, for example, *fbox-32* a muscle specific E3-ubiquitin ligase increased 100-fold (Figure 5B) and *fbox25* increased 10-fold, in line with results from other similar studies [21,47]. The majority of the USPs increased their expression around 2-fold with fasting and recovered pre-fasting levels 24 h after re-feeding (Figure 5A). In contrast, *Usp12a*, showed a transient increase 6 h after re-feeding, and *usp46* and *usp5b* did not change in expression (Figure 5D and E). The overlapping expression profiles of USPs and E3-ubiquitin ligases suggests that USPs play an important role during muscle atrophy. USPs can cleave ubiquitin from proteins, effectively removing the proteasome signal [15]. It is possible that the increase in their abundance in fasted fish is related to fine tuning of the regulation of protein degradation. For example, many USPs targets are essential to maintain cell homeostasis including *mdm4, p53, h2a, h2b, fbw7, fancd2* or *brca2* [17,48-50], and it is possible that these proteins may be relatively spared during fasting.

## Conclusions

We have produced an in depth transcriptome for fast and slow myotomal muscle for the pacu (*Piaractus mesopotamicus*), an important South American aquaculture species. This resource allowed us to characterise the expression signatures of the main myotomal muscle fibre types and identify candidate microsatellite sequences that could be used in breeding programs. The availability of the transcriptome allowed us to identify and analyse the expression of teleost-specific paralogues retained in the Ostariophysi lineage. The transcriptome also enabled a comprehensive study of E3 ubiquitin ligase and USP gene expression in the context of the transition between the fasting (catabolic state) and satiation feeding (anabolic state). We identified differences in expression within gene family members thereby identifying candidates for further investigation.

## Methods

### Fish

For the generation of *de novo* transcriptome fast and slow skeletal muscles were dissected from 5 adult pacu (*Piaractus mesopotamicus*) (1.50 ± 0.61 kg; mean ± SD body mass). Fish were maintained in 1000 litres fibreglass tanks at the Aquaculture Centre of the University of West Paulista (Unoeste) Presidente Prudente, São Paulo, Brazil, under natural photoperiod (12 L: 12D) and temperature (28°C ± 1°C, range). Fast skeletal muscle was dissected from the dorsal epaxial region at 0.5 fork length (FL) (FL, tip of snout to fork in the tail) whereas slow skeletal muscle was dissected from the lateral line and any remains of fast skeletal muscle carefully removed under a dissection microscope to obtain pure slow muscle. Tissues were preserve in RNAlater (Ambion/Applied Biosystems, Oslo, Norway) and frozen at −20°C until further analysis.

For the fasting-re-feeding experiment, 15 g pacu (n = 80) were maintained in duplicate fibreglass 500 litre tanks as described above and fed with a commercial diet until the start the experiment. Fish were fasted for 4 days followed by a period of satiation feeding for 24 h. Fast skeletal muscle was sampled before fasting (−4d), daily during food deprivation (−3d, −2d, −1d, 0d; n = 8) and 6, 12 and 24 h (n = 8) after re-feeding. Dissected fast skeletal muscle was preserved in RNAlater at −20°C until further analysis. All fish were sacrificed according to the Ethical Principles In Animal Research adopted by Brazilian College of Animal Experimentation (COBEA) and was approved by the Ethics Committee on Use of Animals/ Bioscience Institute/Unesp (CEUA = 506).

### Samples sequencing and de novo assembly

Total RNA from adult pacu fast and slow skeletal muscle was used to prepare 10 individual Illumina libraries. Libraries preparation and sequencing was performed at the Centre for Applied Genomics of the Hospital for Sick Children (SickKids), Toronto, Canada. The resulting libraries were paired end sequenced using in an Illumina HiSeq2000.

Raw paired end reads generated were processed by the Department of Informatics of the Centre for Applied Genomics of SickKids hospital. After removing low quality reads, 86% of the paired end reads were *de novo* assembled using Trinity software [51]. RSEM application was used to identify transcript abundance by estimating the number of reads mapped per contig. The DEseq algorithm from the Bioconductor/R packages was used to identify differentially expressed transcripts [52].

### Functional annotation

Contigs were annotated using Blast2GO software [53]. Sequences were blasted against the NCBI non-redundant (nr) database using BLASTx with an e-value cut-off of 10^−3^ followed by functional annotation using software default parameters [53]. Contigs were mapped against the known vertebrate metabolic and molecular pathways using the online KEGG Automatic Annotation Server (KAAS) [54]. KAAS annotation was performed using the single-directional Best Hit (SBH) method against *Homo sapiens*, *Pan troglodites, Mus musculus, Rattus norvegicus, Sus scrofa, Gallus gallus, Meleagris gallopavo, Danio rerio* and *Xenopus laevis.*

### Identification of complete coding sequence

Annotated contigs were blasted against the Zebrafish complete proteome [22] using tBLASTn algorithm in BioEdit software [55]. BLAST alignments were explored to evaluate the percentage of conding sequence cover by the contig compared with its zebrafish orthologue. Sequences with more than 90% of coverage were considered as complete coding sequence (CDS). The CDS amino acids sequence was predicted using the Virtual Ribosome server [56].

### Microsatellite identification

Sequences successfully annotated covering >90% of the CDS were investigated for SSR using msatcommander-1.0.2-alpha [57].

### RNA Extraction and cDNA synthesis

Total RNA was extracted using 1 ml TRIsure (Bioline, London, UK) following the manufacturer’s recommendations. Integrity was confirmed by ethidium bromide gel electrophoresis of 1 μg of total RNA. Concentration, 260/280 and 260/230 ratios were estimated using a NanoDrop 1000 spectrophotometer (Thermo Fischer Scientific, Waltman, MA). All RNA samples used had 260/280 nm and 260/230 ratios above 1.9 and 2.2 respectively. 1 μg of total RNA was reverse transcribed into cDNA for 30 min at 42°C using a Quantitect (QIAGEN, Manchester, UK) reverse transcription kit following manufacture’s recommendations including a genomic DNA wipe-out step. To ensure that no genomic DNA was present in the samples a RT- control without the reverse transcriptase enzyme was performed simultaneously.

### Quantitative Real-time PCR

The following procedures were compliant with the minimal information requirements for publication of quantitative PCR guidelines [58]. Primers were designed to have a Tm of 60°C using Net primer online server (Premier BioSoft). Where possible primers were designed to cross exon-exon junctions. Exon-exon junctions were predicted by aligning the pacu contig against their zebrafish orthologue complete gene sequence retrieved from Ensembl [22] using Spidey online server [59]. Primers pairs, amplicon size and efficiency are listed in Additional file 12.

Quantitative PCR (qPCR) was performed using a MX3005P qPCR machine (Agilent, La Jolla, CA, USA). Each qPCR reaction contained 7.5 μl of SensiFast (Bioline) Master Mix, 6 μl cDNA (80-fold dilution and 40-fold dilution for *igf* genes) and 0.75 μl of each primer at 500 nM to a final volume of 15 μl. Duplicate reactions were performed in 96-well plates (Agilent) with the following protocol: initial activation 95°C for 2 min followed by 40 cycles of 5 s at 95°C, 20s at 65°C. The qPCR was followed by a dissociation-melting curve from 60 to 95°C to confirm that a single product was amplified. Control reactions included no-template and RT- were simultaneously amplified.

*Ribosomal protein 13* and *19* (*rpl13*, *rpl19*), *Peptidylprolyl isomerase Aa* (*ppiaa*), *elongation factor 1 alpha*, *Glyceraldehyde-3-phosphate dehydrogenase* (*gapdh*) and *hypoxanthine phosphoribosyltransferase 1* (*hprt1*) were tested as reference genes. BestKeeper [60] analysis showed that *hprt1* was the most stable reference gene and was used for data normalisation. Relative expression was calculated using the Pfalff method [61].

### Teleost specific paralogues identification

Contigs were blasted (BLASTx) against the Zebrafish proteome using BioEdit software with an e-value threshold of e^−40^ [55].

To confirm that contigs found were truly paralogues amino acids sequences from potential pacu paralogues were blasted (BLASTp) against the zebrafish (*Danio rerio*), stickleback (*Gasterosteus aculeatus*), takifugu (*Takifugu rubripes*), medaka (*Oryzias latipes*), green pufferfish (*Tetraodon nigroviridis*), chicken (*Gallus gallus*), frog (*Xenopus laevis*) and human (*Homo sapiens*) proteomes using Ensembl BLAST server [22]. Best hits amino acids sequences from each proteome were retrieved. Sequences were aligned using the MAFFT online server [62]. Phylogenetic trees were constructed using a Maximum Likelihood analysis using PhyML online server combined with the G-Blocks option to cure unreliable aligned sections [63]. For each case the best evolutionary model was estimated using MEGA5 [64].

### Statistical analysis

Global DGE statistic analysis was performed using DEseq package from R-Bioconductor [52,65]. For testing specific hypothesis involving differential mapping of specific pathways gene expression significance was tested using *t*-test, or Mann–Whitney *U* test when parametric were not fulfilled, followed by a Benjamin-Hochberg correction (False Discovery Rate, FDR). Differences between time-points in qPCR expression during fasting re-feeding were tested using Kruskal-Wallis test. Differences were considered significant when FDR, for differential mapping, or p-value, for qPCR expression, were <0.05. Gene expression data was clustered using an unsupervised hierarchical clustering algorithm using PermutMatrix [66].

## Additional files

Additional file 1:
**Annotated transcriptome.** Annotated nucleotide using blast2go from the pacu slow and fast muscle transcriptome. Additional file 2:
**KEEG Automatic Annotation Sever results.** Hierarchical information of the sequences from annotated transcriptome mapped to the KEGG pathways maps using the automatic annotation tool KAAS [54]. Additional file 3:
**Simple Sequence Repeats Identification.** Simple Sequence Repeats identified in contigs containing over 90% of the CDS using Msatcommander software [57]. Additional file 4:
**Teleost-specific paralogues sequences and phylogenetic trees.** Supplementary file contains the amino acids sequences for the pacu paralogues and the corresponding orthologues for zebrafish, stickleback, tilapia, green puffer fish and human, their MAFFT alignments and individual phylogenetic trees. Additional file 5:
**Fast and slow skeletal muscle global digital gene expression analysis.** Global digital gene expression analysis between fast and slow skeletal muscle was performed using the number of reads mapped normalized by length and library size was investigated using R-Bioconductor. Normalized counts for individual animals per contig are shown. Differences between tissues were analysed using the DESEQ package. Significant differences were considered when FDR < 0.05. Additional file 6:
**Fast and slow skeletal muscle Gene Ontology (GO) analysis.** GO analysis was performed for differently expressed contigs originating from the fast and slow skeletal muscle comparison. Significantly enriched categories were investigated using Fisher Exact Test with the whole annotated transcriptome as reference and considered significant when FDR < 0.05. Additional file 7:
**Digital gene expression of metabolic pathways in fast and slow skeletal muscle.** Components from the different metabolic pathways were extracted from KAAS annotation results. Analysis between fast and slow skeletal muscle was performed using the number of reads mapped normalized by length and library size. Normalized counts for individual animals per contig are shown. Differences between tissues were analysed by *t*-test followed by a False Discovery Rate correction. Significant differences were considered when FDR < 0.05. Additional file 8:
**Schematic representation of fast and slow skeletal muscle main metabolic pathways.** Metabolic genes represented in the transcriptome mapped into a reconstruction of the main metabolic pathways. “Red circles” and “Empty circles” indicate components significantly higher in slow and fast skeletal muscle respectively muscle (FDR < 0.05). “Green circles” indicate components with no significant differences between fibre types. Additional file 9:
**Teleost-specific paralogues digital gene expression.** Teleost-specific paralogues digital gene expression in slow and fast skeletal muscle. Comparisons were made between paralogue pairs and fibres types. Analysis between fast and slow skeletal muscle was performed using the number of reads mapped normalized by length and library size. Normalized counts for individual animals per contig are shown. Differences between tissues were analysed by *t*-test followed by a False Discovery Rate correction. Significant differences were considered when FDR < 0.05. Additional file 10:
**Pi3k/mTor pathway components digital gene expression.** Pi3k/mTor components digital gene expression for slow and fast skeletal muscle. Analysis between fast and slow skeletal muscle was performed using the number of reads mapped normalized by length and library size. Normalized counts for individual animals per contig are shown. Differences between tissues were analysed by *t*-test followed by a False Discovery Rate correction. Significant differences were considered when FDR < 0.05. Additional file 11:
**Pacu ubiquitin specific proteases functional domains and digital gene expression.** Ubiquitin specific proteases (USP) digital gene expression analysis between slow and fast skeletal muscle. Analysis between fast and slow skeletal muscle was performed using the number of reads mapped normalized by length and library size. Normalized counts for individual animals per contig are shown. Differences between tissues were analysed by *t*-test followed by a False Discovery Rate correction. Significant differences were considered when FDR < 0.05. Pacu, zebrafish and human protein USP functional domains were determined using InterProScan webserver. Relative gene expression was analysed for *fbox25, huwe, mafbx, murf1a, murf1b, syah1, syyna, trip12, ufd2, igf1, igf2a, igf2b, igf3, igf1ra, igf1rb, usp2a, usp2b, usp4, usp5a, usp5b, usp8, usp9, usp11, usp12b, usp12b, usp14, usp16, usp19, usp21, usp24, usp28, usp30, usp36* and *usp46*, Values represents mean ± SE (n = 8 fish). Additional file 12:
**qPCR primers sequence of target genes:** ***fbox25, huwe, mafbx, murf1a, murf1b, syah1, syyna, trip12, ufd2, igf1, igf2a, igf2b, igf3, igf1ra, igf1rb, usp2a, usp2b, usp4, usp5a, usp5b, usp8, usp9, usp11, usp12b, usp12b, usp14, usp16, usp19, usp21, usp24, usp28, usp30, usp36 and usp46 and housekeeping genes: Ef1a, Polr2a, Gapdh, Hprt1, ppiaa, sdha, Rpl13, Rpl19.***
