# Supplementary figures and images for: Characterization of the transcriptome of fast and slow muscle myotomal fibres in the pacu (Piaractus mesopotamicus)
Source: BMC Genomics. 2015 Mar 14;16(1):182. doi: 10.1186/s12864-015-1423-6 (PMC4372171; doi:10.1186/s12864-015-1423-6)

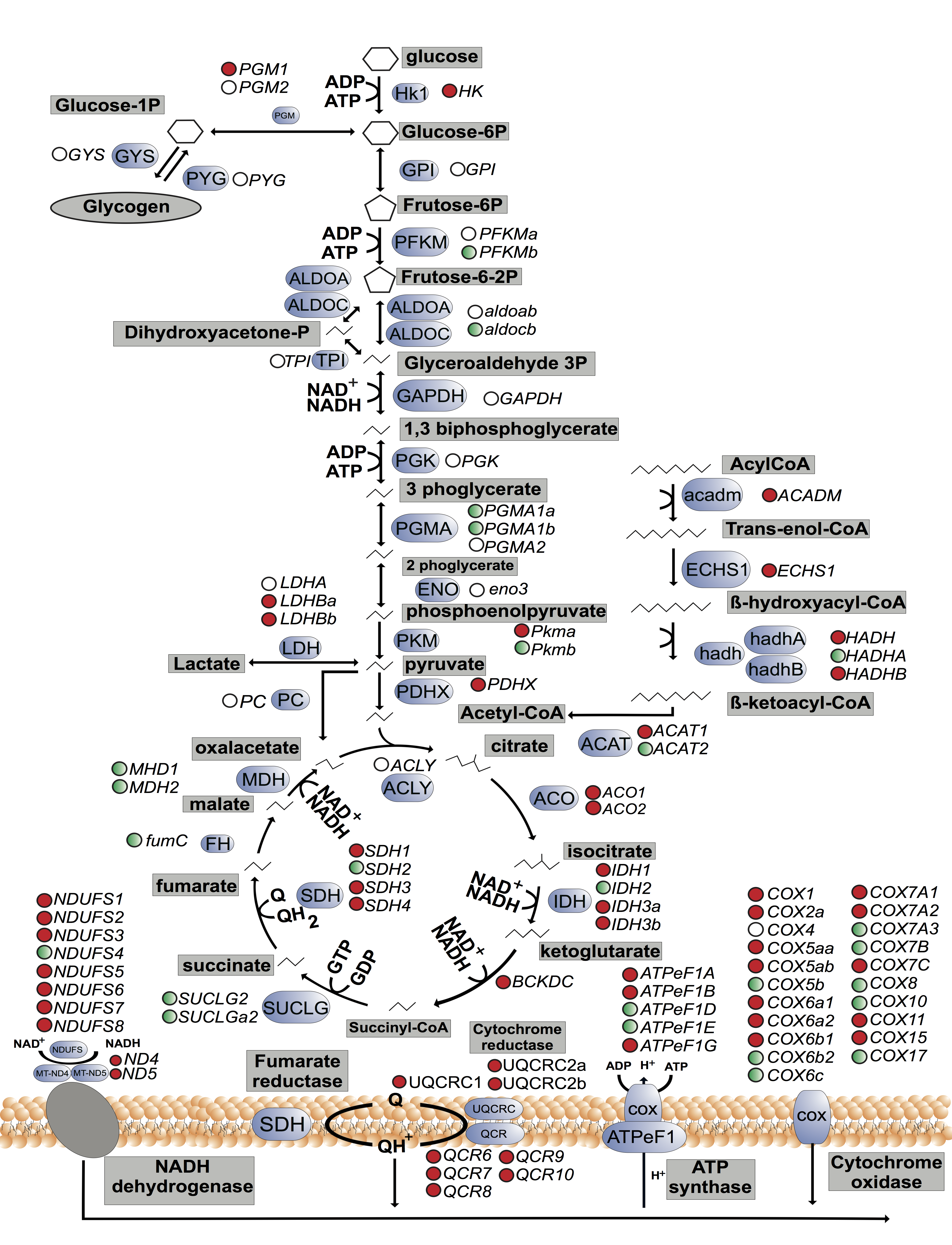

Supplement: Additional file 8: — Schematic representation of fast and slow skeletal muscle main metabolic pathways. Metabolic genes represented in the transcriptome mapped into a reconstruction of the main metabolic pathways. “Red circles” and “Empty circles” indicate components significantly higher in slow and fast skeletal muscle respectively muscle (FDR < 0.05). “Green circles” indicate components with no significant differences between fibre types. [file 12864_2015_1423_MOESM8_ESM.tiff]

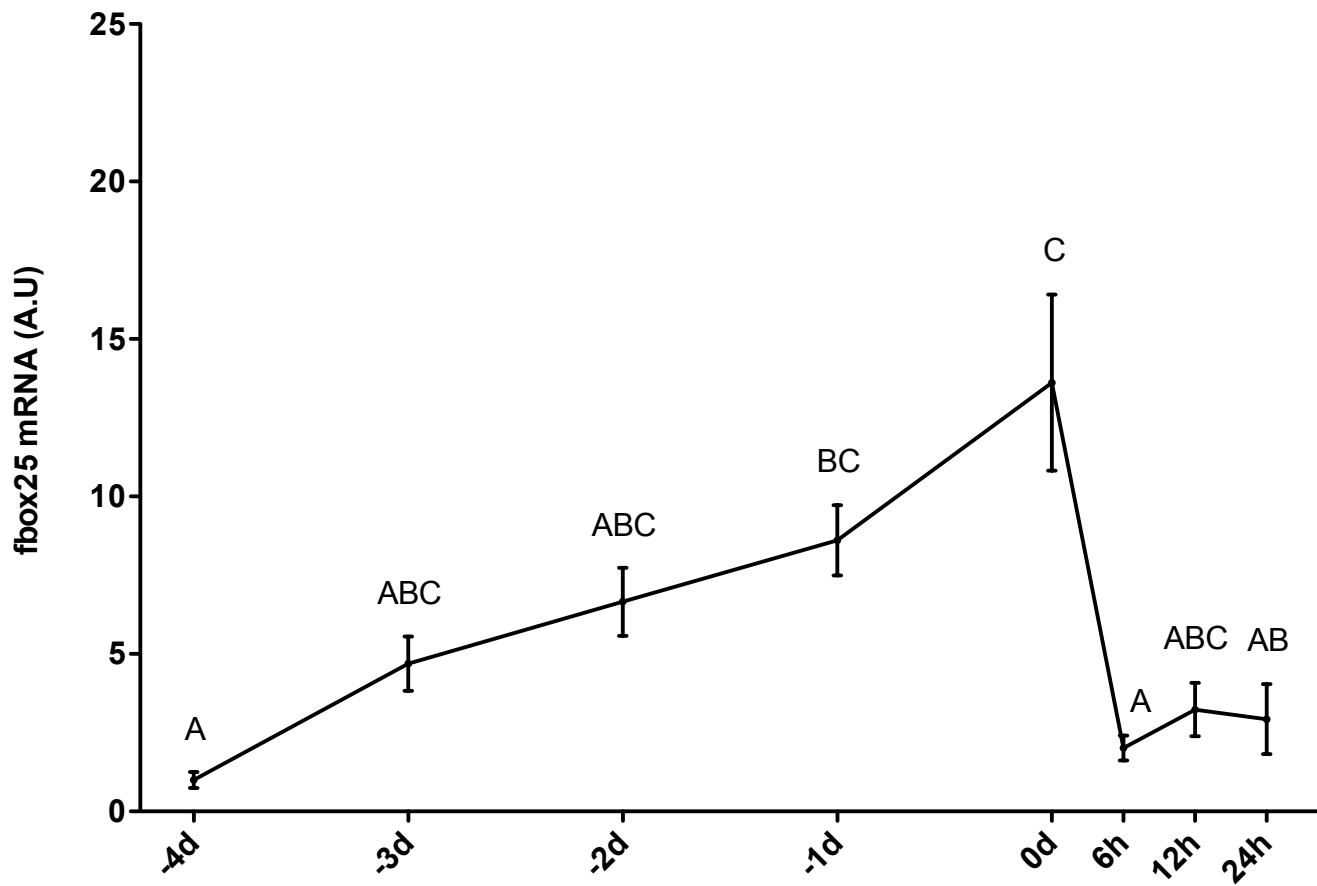

Supplement: Additional file 11: — Pacu ubiquitin specific proteases functional domains and digital gene expression. Ubiquitin specific proteases (USP) digital gene expression analysis between slow and fast skeletal muscle. Analysis between fast and slow skeletal muscle was performed using the number of reads mapped normalized by length and library size. Normalized counts for individual animals per contig are shown. Differences between tissues were analysed by t-test followed by a False Discovery Rate correction. Significant differences were considered when FDR < 0.05. Pacu, zebrafish and human protein USP functional domains were determined using InterProScan webserver. Relative gene expression was analysed for fbox25, huwe, mafbx, murf1a, murf1b, syah1, syyna, trip12, ufd2, igf1, igf2a, igf2b, igf3, igf1ra, igf1rb, usp2a, usp2b, usp4, usp5a, usp5b, usp8, usp9, usp11, usp12b, usp12b, usp14, usp16, usp19, usp21, usp24, usp28, usp30, usp36 and usp46, Values represents mean ± SE (n = 8 fish). [file 12864_2015_1423_MOESM11_ESM.zip › Supplementary_file_11_USPs_domains/Graphs_gene_expression_E3-ubiquitin_USP_IGF/fbox25.pdf]

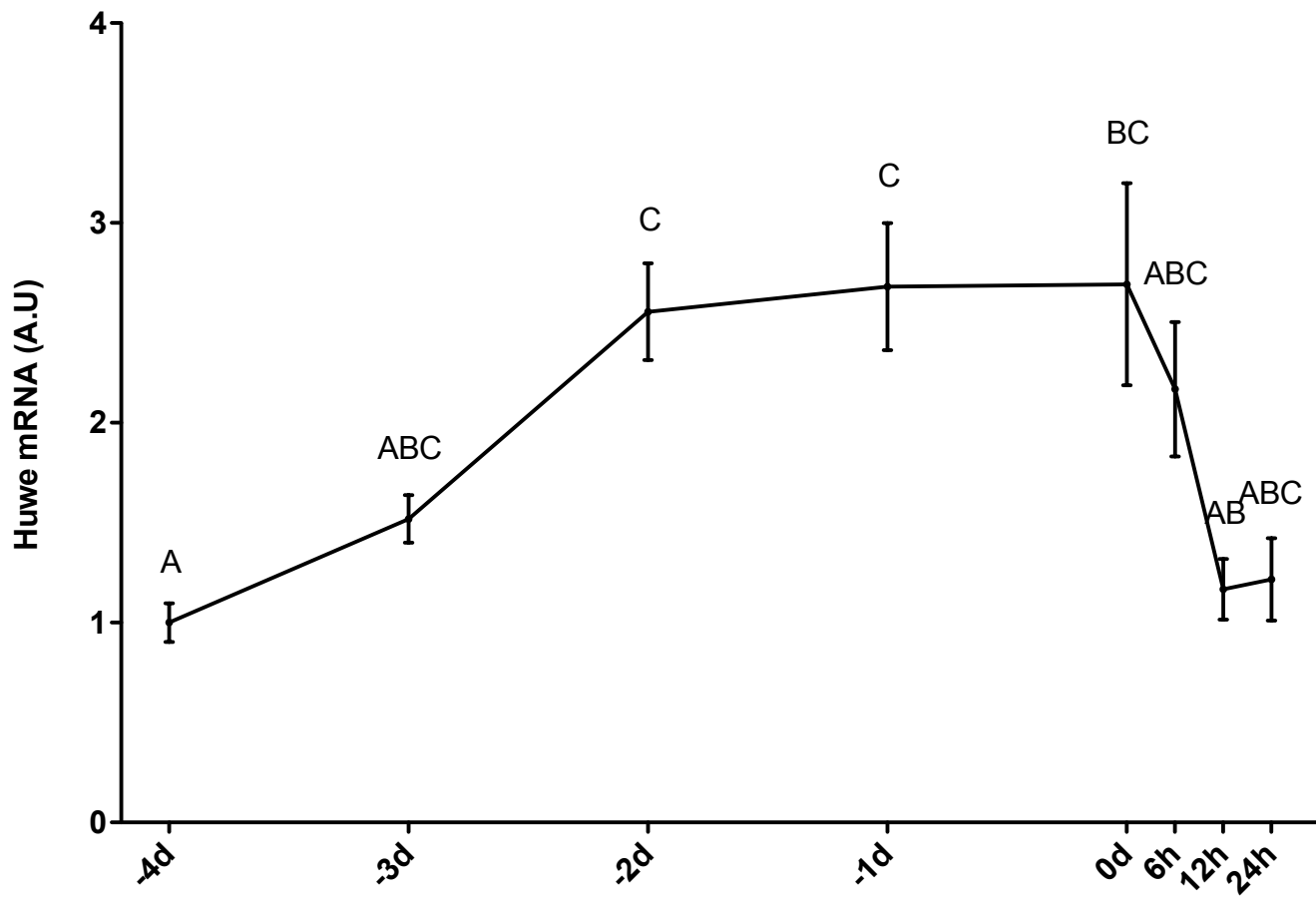

Supplement: Additional file 11: — Pacu ubiquitin specific proteases functional domains and digital gene expression. Ubiquitin specific proteases (USP) digital gene expression analysis between slow and fast skeletal muscle. Analysis between fast and slow skeletal muscle was performed using the number of reads mapped normalized by length and library size. Normalized counts for individual animals per contig are shown. Differences between tissues were analysed by t-test followed by a False Discovery Rate correction. Significant differences were considered when FDR < 0.05. Pacu, zebrafish and human protein USP functional domains were determined using InterProScan webserver. Relative gene expression was analysed for fbox25, huwe, mafbx, murf1a, murf1b, syah1, syyna, trip12, ufd2, igf1, igf2a, igf2b, igf3, igf1ra, igf1rb, usp2a, usp2b, usp4, usp5a, usp5b, usp8, usp9, usp11, usp12b, usp12b, usp14, usp16, usp19, usp21, usp24, usp28, usp30, usp36 and usp46, Values represents mean ± SE (n = 8 fish). [file 12864_2015_1423_MOESM11_ESM.zip › Supplementary_file_11_USPs_domains/Graphs_gene_expression_E3-ubiquitin_USP_IGF/Huwe.pdf]

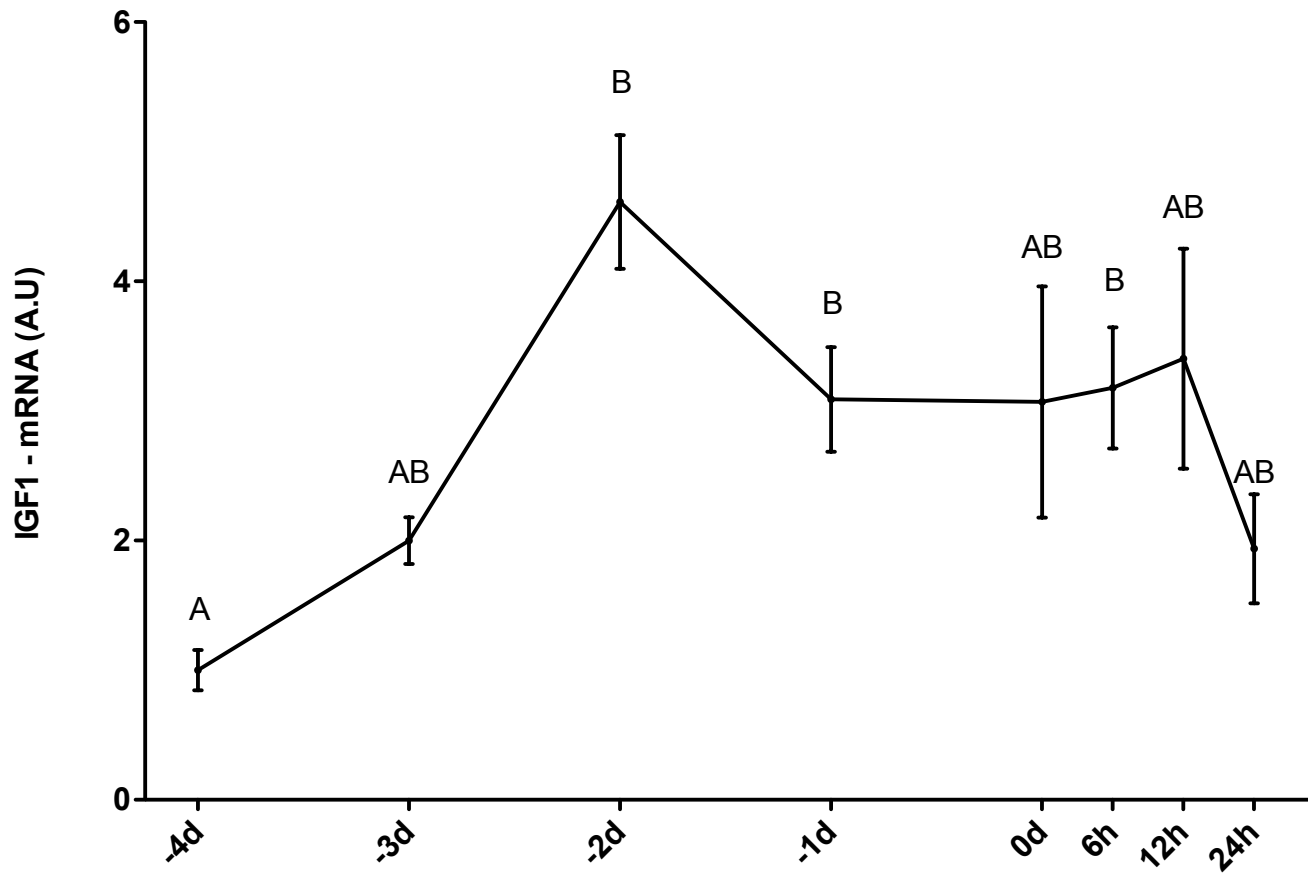

Supplement: Additional file 11: — Pacu ubiquitin specific proteases functional domains and digital gene expression. Ubiquitin specific proteases (USP) digital gene expression analysis between slow and fast skeletal muscle. Analysis between fast and slow skeletal muscle was performed using the number of reads mapped normalized by length and library size. Normalized counts for individual animals per contig are shown. Differences between tissues were analysed by t-test followed by a False Discovery Rate correction. Significant differences were considered when FDR < 0.05. Pacu, zebrafish and human protein USP functional domains were determined using InterProScan webserver. Relative gene expression was analysed for fbox25, huwe, mafbx, murf1a, murf1b, syah1, syyna, trip12, ufd2, igf1, igf2a, igf2b, igf3, igf1ra, igf1rb, usp2a, usp2b, usp4, usp5a, usp5b, usp8, usp9, usp11, usp12b, usp12b, usp14, usp16, usp19, usp21, usp24, usp28, usp30, usp36 and usp46, Values represents mean ± SE (n = 8 fish). [file 12864_2015_1423_MOESM11_ESM.zip › Supplementary_file_11_USPs_domains/Graphs_gene_expression_E3-ubiquitin_USP_IGF/IGF1.pdf]

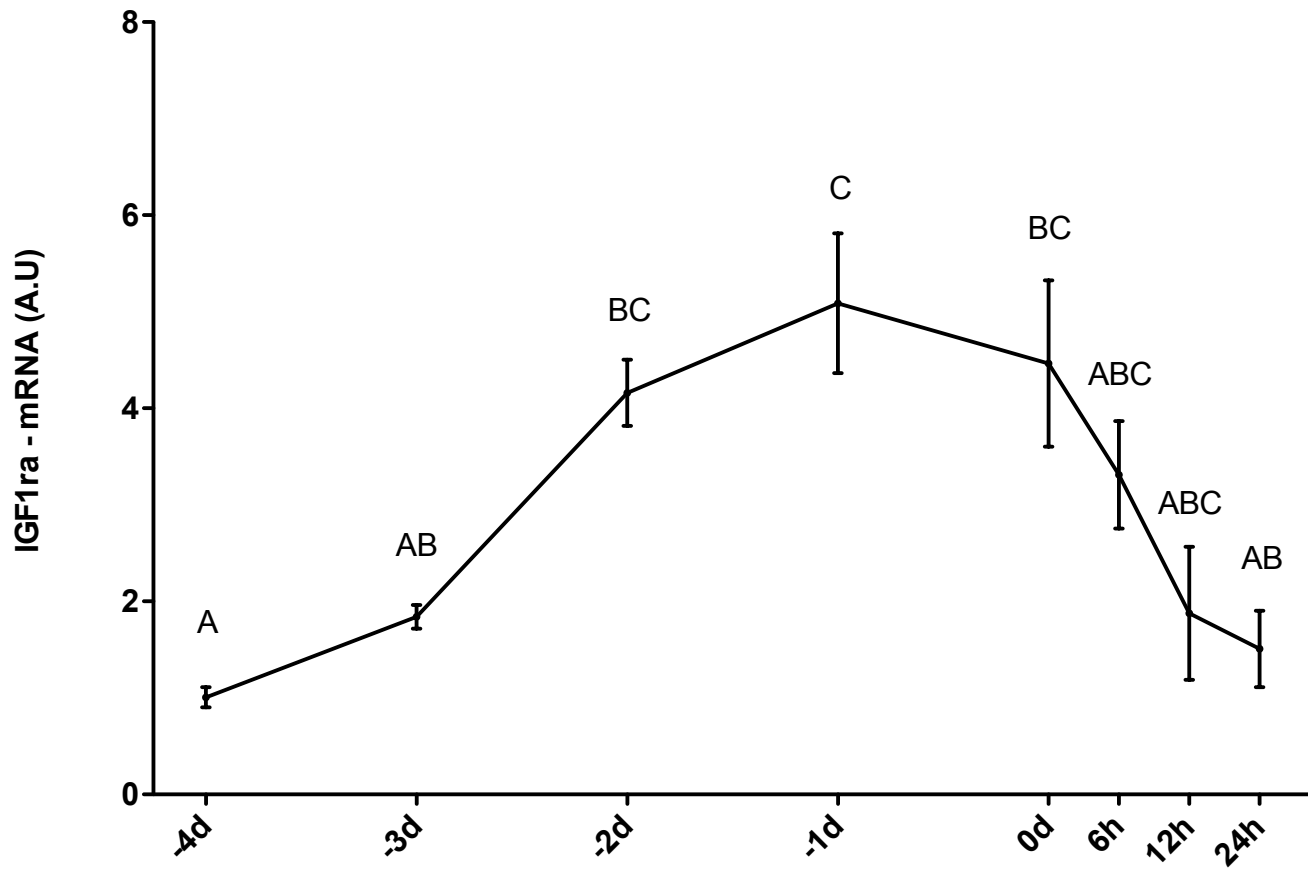

Supplement: Additional file 11: — Pacu ubiquitin specific proteases functional domains and digital gene expression. Ubiquitin specific proteases (USP) digital gene expression analysis between slow and fast skeletal muscle. Analysis between fast and slow skeletal muscle was performed using the number of reads mapped normalized by length and library size. Normalized counts for individual animals per contig are shown. Differences between tissues were analysed by t-test followed by a False Discovery Rate correction. Significant differences were considered when FDR < 0.05. Pacu, zebrafish and human protein USP functional domains were determined using InterProScan webserver. Relative gene expression was analysed for fbox25, huwe, mafbx, murf1a, murf1b, syah1, syyna, trip12, ufd2, igf1, igf2a, igf2b, igf3, igf1ra, igf1rb, usp2a, usp2b, usp4, usp5a, usp5b, usp8, usp9, usp11, usp12b, usp12b, usp14, usp16, usp19, usp21, usp24, usp28, usp30, usp36 and usp46, Values represents mean ± SE (n = 8 fish). [file 12864_2015_1423_MOESM11_ESM.zip › Supplementary_file_11_USPs_domains/Graphs_gene_expression_E3-ubiquitin_USP_IGF/IGF1ra.pdf]

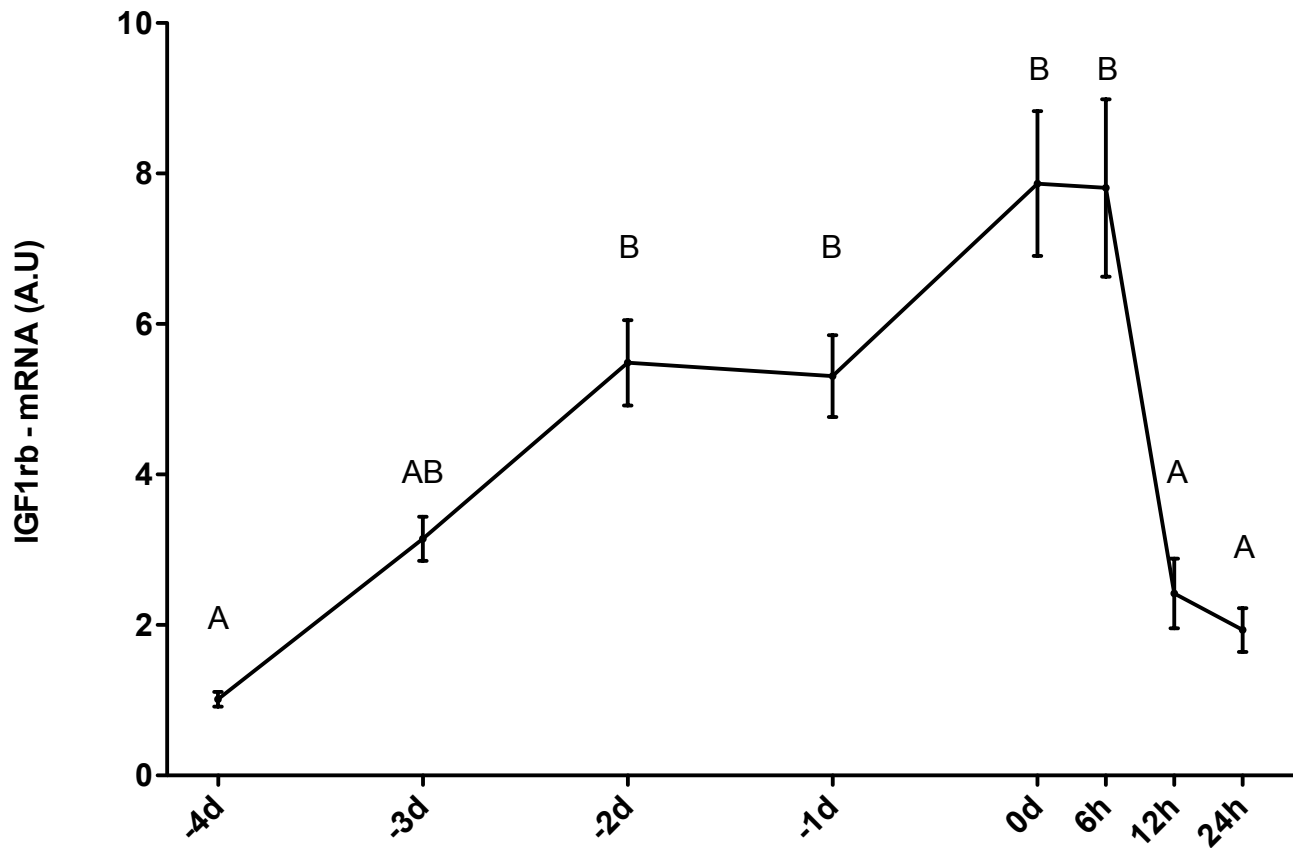

Supplement: Additional file 11: — Pacu ubiquitin specific proteases functional domains and digital gene expression. Ubiquitin specific proteases (USP) digital gene expression analysis between slow and fast skeletal muscle. Analysis between fast and slow skeletal muscle was performed using the number of reads mapped normalized by length and library size. Normalized counts for individual animals per contig are shown. Differences between tissues were analysed by t-test followed by a False Discovery Rate correction. Significant differences were considered when FDR < 0.05. Pacu, zebrafish and human protein USP functional domains were determined using InterProScan webserver. Relative gene expression was analysed for fbox25, huwe, mafbx, murf1a, murf1b, syah1, syyna, trip12, ufd2, igf1, igf2a, igf2b, igf3, igf1ra, igf1rb, usp2a, usp2b, usp4, usp5a, usp5b, usp8, usp9, usp11, usp12b, usp12b, usp14, usp16, usp19, usp21, usp24, usp28, usp30, usp36 and usp46, Values represents mean ± SE (n = 8 fish). [file 12864_2015_1423_MOESM11_ESM.zip › Supplementary_file_11_USPs_domains/Graphs_gene_expression_E3-ubiquitin_USP_IGF/IGF1rb.pdf]

IGF2a - mRNA (A.U)

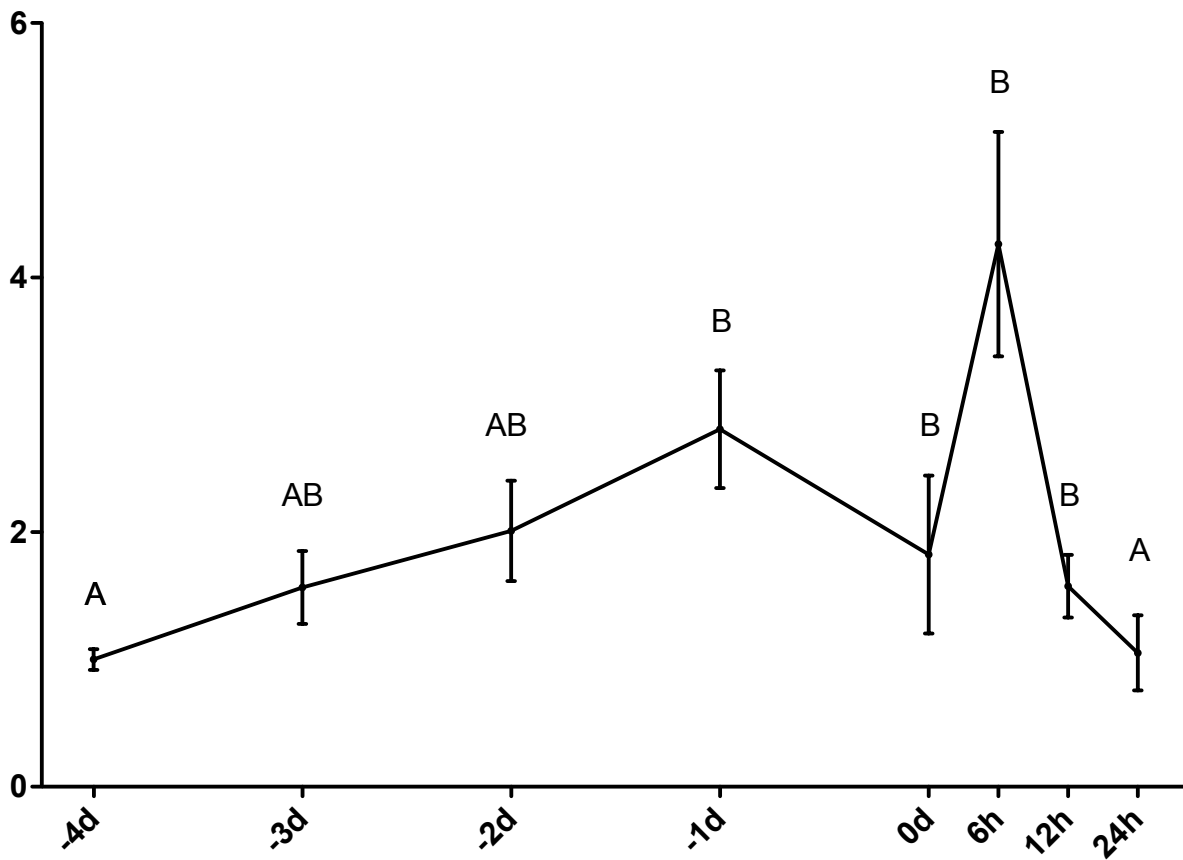

Supplement: Additional file 11: — Pacu ubiquitin specific proteases functional domains and digital gene expression. Ubiquitin specific proteases (USP) digital gene expression analysis between slow and fast skeletal muscle. Analysis between fast and slow skeletal muscle was performed using the number of reads mapped normalized by length and library size. Normalized counts for individual animals per contig are shown. Differences between tissues were analysed by t-test followed by a False Discovery Rate correction. Significant differences were considered when FDR < 0.05. Pacu, zebrafish and human protein USP functional domains were determined using InterProScan webserver. Relative gene expression was analysed for fbox25, huwe, mafbx, murf1a, murf1b, syah1, syyna, trip12, ufd2, igf1, igf2a, igf2b, igf3, igf1ra, igf1rb, usp2a, usp2b, usp4, usp5a, usp5b, usp8, usp9, usp11, usp12b, usp12b, usp14, usp16, usp19, usp21, usp24, usp28, usp30, usp36 and usp46, Values represents mean ± SE (n = 8 fish). [file 12864_2015_1423_MOESM11_ESM.zip › Supplementary_file_11_USPs_domains/Graphs_gene_expression_E3-ubiquitin_USP_IGF/IGF2a.pdf]

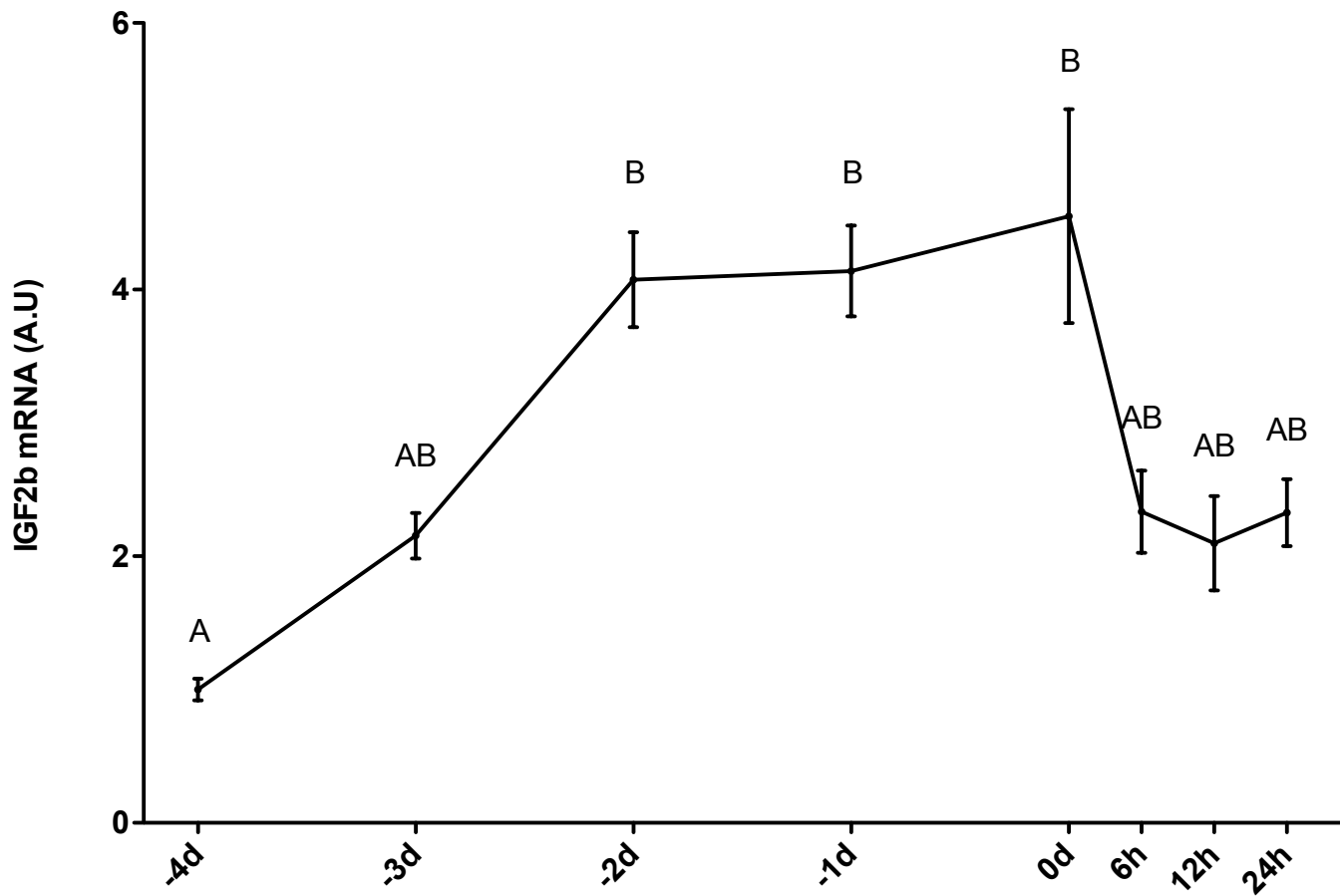

Supplement: Additional file 11: — Pacu ubiquitin specific proteases functional domains and digital gene expression. Ubiquitin specific proteases (USP) digital gene expression analysis between slow and fast skeletal muscle. Analysis between fast and slow skeletal muscle was performed using the number of reads mapped normalized by length and library size. Normalized counts for individual animals per contig are shown. Differences between tissues were analysed by t-test followed by a False Discovery Rate correction. Significant differences were considered when FDR < 0.05. Pacu, zebrafish and human protein USP functional domains were determined using InterProScan webserver. Relative gene expression was analysed for fbox25, huwe, mafbx, murf1a, murf1b, syah1, syyna, trip12, ufd2, igf1, igf2a, igf2b, igf3, igf1ra, igf1rb, usp2a, usp2b, usp4, usp5a, usp5b, usp8, usp9, usp11, usp12b, usp12b, usp14, usp16, usp19, usp21, usp24, usp28, usp30, usp36 and usp46, Values represents mean ± SE (n = 8 fish). [file 12864_2015_1423_MOESM11_ESM.zip › Supplementary_file_11_USPs_domains/Graphs_gene_expression_E3-ubiquitin_USP_IGF/IGF2b.pdf]

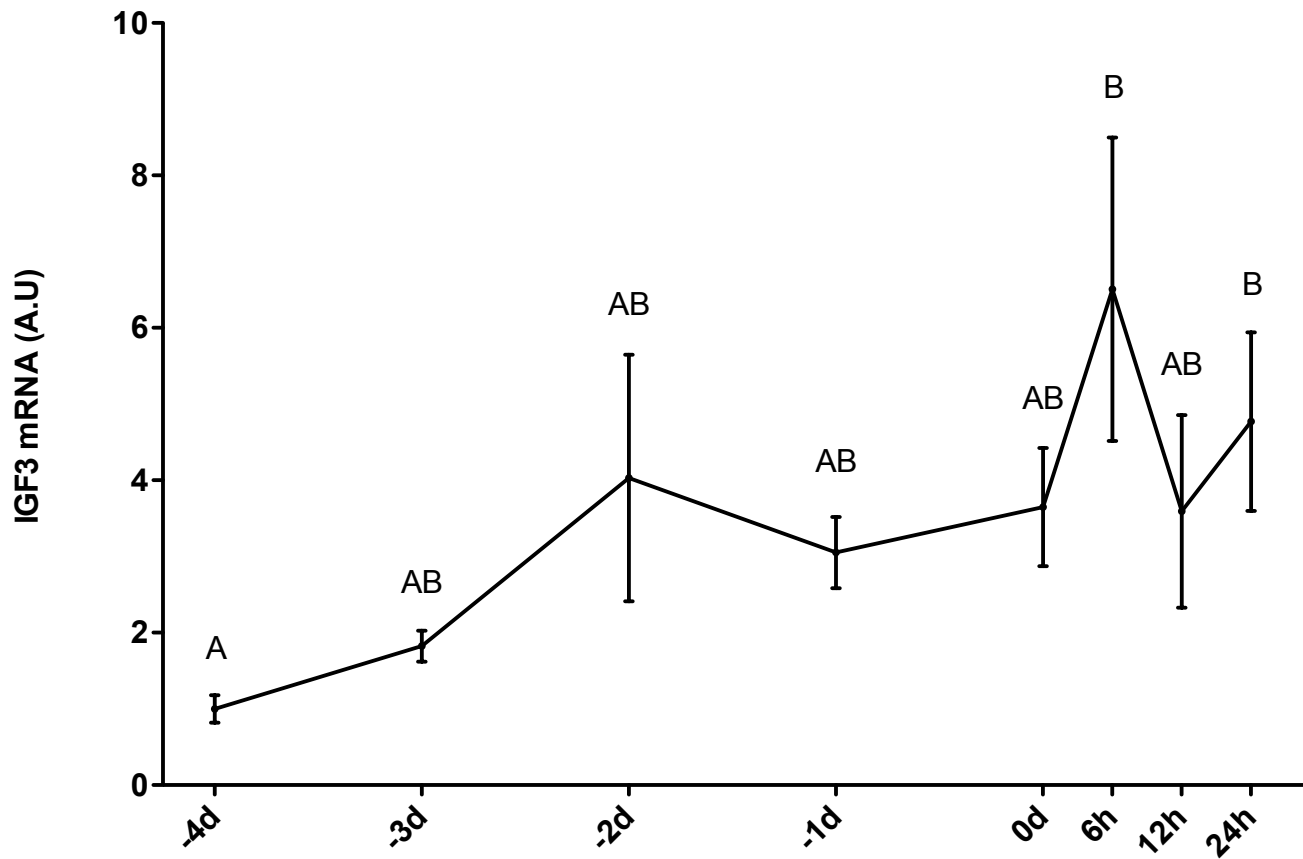

Supplement: Additional file 11: — Pacu ubiquitin specific proteases functional domains and digital gene expression. Ubiquitin specific proteases (USP) digital gene expression analysis between slow and fast skeletal muscle. Analysis between fast and slow skeletal muscle was performed using the number of reads mapped normalized by length and library size. Normalized counts for individual animals per contig are shown. Differences between tissues were analysed by t-test followed by a False Discovery Rate correction. Significant differences were considered when FDR < 0.05. Pacu, zebrafish and human protein USP functional domains were determined using InterProScan webserver. Relative gene expression was analysed for fbox25, huwe, mafbx, murf1a, murf1b, syah1, syyna, trip12, ufd2, igf1, igf2a, igf2b, igf3, igf1ra, igf1rb, usp2a, usp2b, usp4, usp5a, usp5b, usp8, usp9, usp11, usp12b, usp12b, usp14, usp16, usp19, usp21, usp24, usp28, usp30, usp36 and usp46, Values represents mean ± SE (n = 8 fish). [file 12864_2015_1423_MOESM11_ESM.zip › Supplementary_file_11_USPs_domains/Graphs_gene_expression_E3-ubiquitin_USP_IGF/IGF3.pdf]

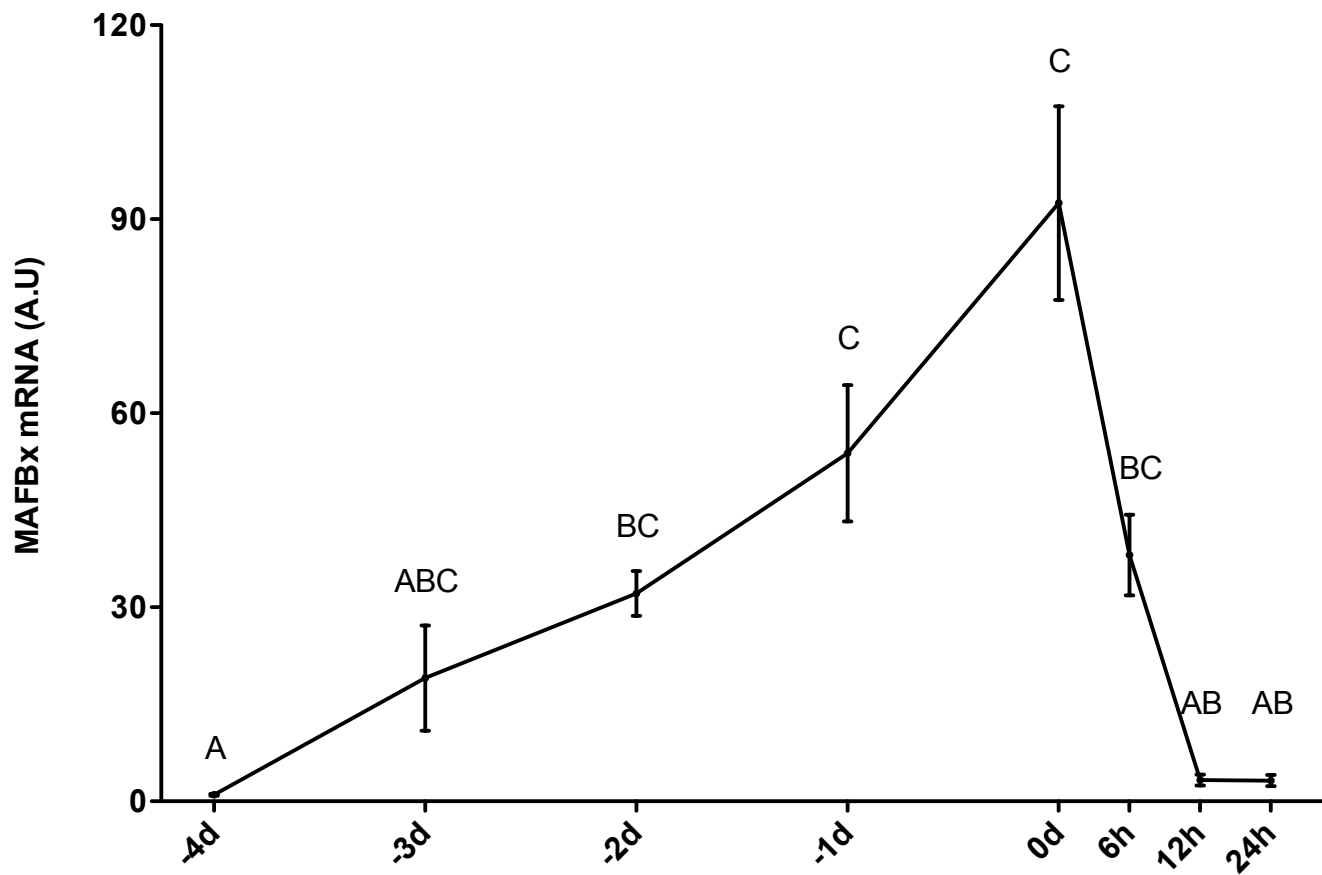

Supplement: Additional file 11: — Pacu ubiquitin specific proteases functional domains and digital gene expression. Ubiquitin specific proteases (USP) digital gene expression analysis between slow and fast skeletal muscle. Analysis between fast and slow skeletal muscle was performed using the number of reads mapped normalized by length and library size. Normalized counts for individual animals per contig are shown. Differences between tissues were analysed by t-test followed by a False Discovery Rate correction. Significant differences were considered when FDR < 0.05. Pacu, zebrafish and human protein USP functional domains were determined using InterProScan webserver. Relative gene expression was analysed for fbox25, huwe, mafbx, murf1a, murf1b, syah1, syyna, trip12, ufd2, igf1, igf2a, igf2b, igf3, igf1ra, igf1rb, usp2a, usp2b, usp4, usp5a, usp5b, usp8, usp9, usp11, usp12b, usp12b, usp14, usp16, usp19, usp21, usp24, usp28, usp30, usp36 and usp46, Values represents mean ± SE (n = 8 fish). [file 12864_2015_1423_MOESM11_ESM.zip › Supplementary_file_11_USPs_domains/Graphs_gene_expression_E3-ubiquitin_USP_IGF/MAFBx.pdf]

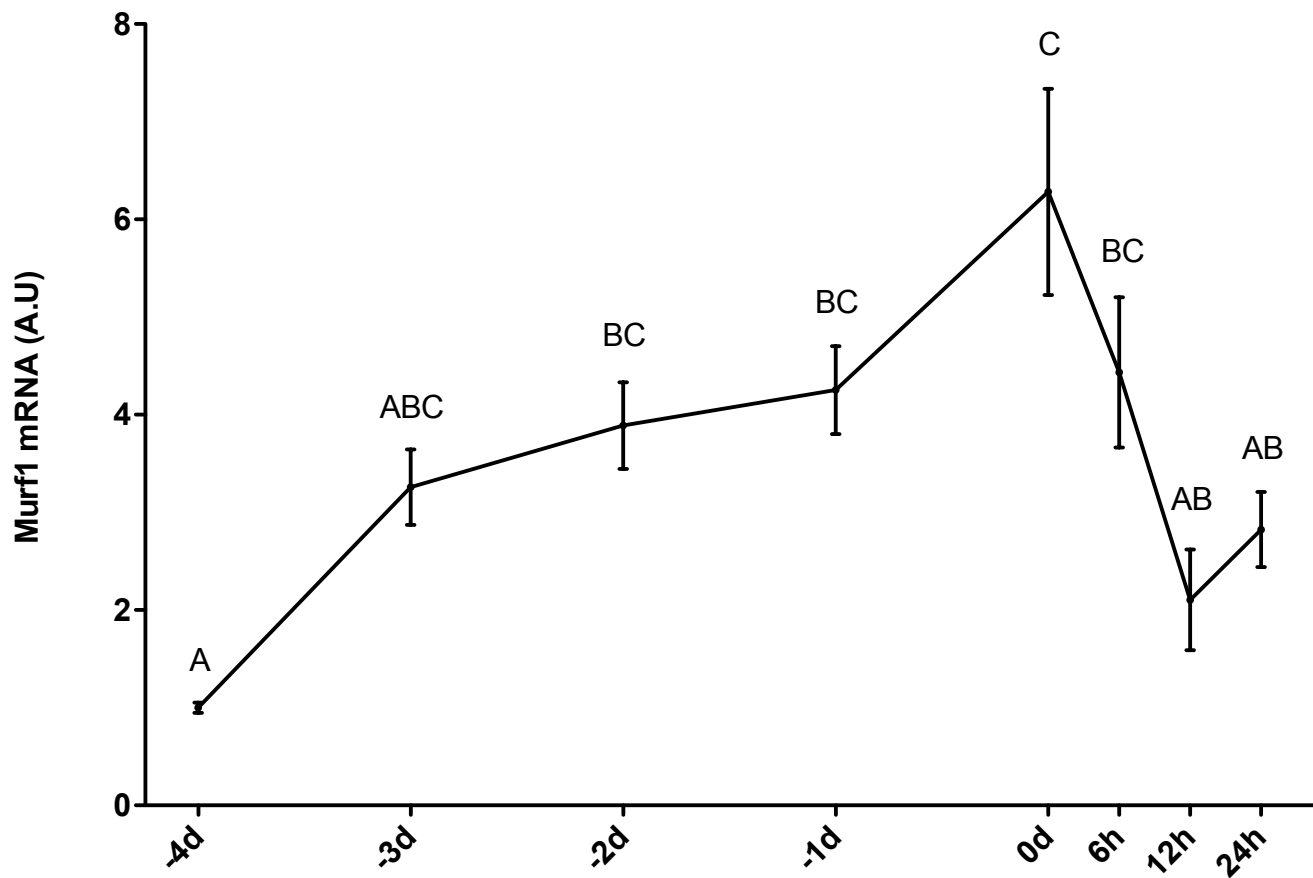

Supplement: Additional file 11: — Pacu ubiquitin specific proteases functional domains and digital gene expression. Ubiquitin specific proteases (USP) digital gene expression analysis between slow and fast skeletal muscle. Analysis between fast and slow skeletal muscle was performed using the number of reads mapped normalized by length and library size. Normalized counts for individual animals per contig are shown. Differences between tissues were analysed by t-test followed by a False Discovery Rate correction. Significant differences were considered when FDR < 0.05. Pacu, zebrafish and human protein USP functional domains were determined using InterProScan webserver. Relative gene expression was analysed for fbox25, huwe, mafbx, murf1a, murf1b, syah1, syyna, trip12, ufd2, igf1, igf2a, igf2b, igf3, igf1ra, igf1rb, usp2a, usp2b, usp4, usp5a, usp5b, usp8, usp9, usp11, usp12b, usp12b, usp14, usp16, usp19, usp21, usp24, usp28, usp30, usp36 and usp46, Values represents mean ± SE (n = 8 fish). [file 12864_2015_1423_MOESM11_ESM.zip › Supplementary_file_11_USPs_domains/Graphs_gene_expression_E3-ubiquitin_USP_IGF/Murf1a.pdf]

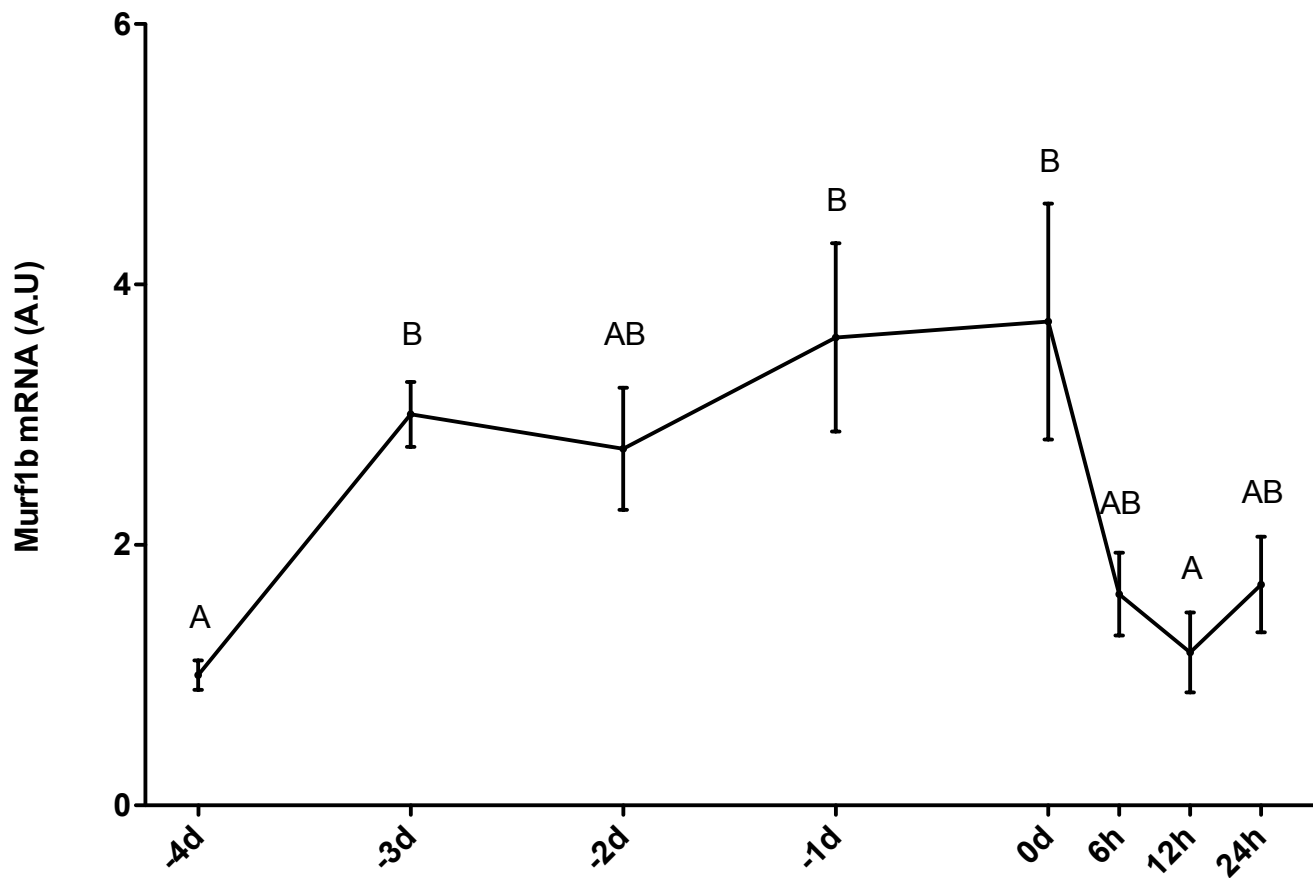

Supplement: Additional file 11: — Pacu ubiquitin specific proteases functional domains and digital gene expression. Ubiquitin specific proteases (USP) digital gene expression analysis between slow and fast skeletal muscle. Analysis between fast and slow skeletal muscle was performed using the number of reads mapped normalized by length and library size. Normalized counts for individual animals per contig are shown. Differences between tissues were analysed by t-test followed by a False Discovery Rate correction. Significant differences were considered when FDR < 0.05. Pacu, zebrafish and human protein USP functional domains were determined using InterProScan webserver. Relative gene expression was analysed for fbox25, huwe, mafbx, murf1a, murf1b, syah1, syyna, trip12, ufd2, igf1, igf2a, igf2b, igf3, igf1ra, igf1rb, usp2a, usp2b, usp4, usp5a, usp5b, usp8, usp9, usp11, usp12b, usp12b, usp14, usp16, usp19, usp21, usp24, usp28, usp30, usp36 and usp46, Values represents mean ± SE (n = 8 fish). [file 12864_2015_1423_MOESM11_ESM.zip › Supplementary_file_11_USPs_domains/Graphs_gene_expression_E3-ubiquitin_USP_IGF/Murf1b.pdf]

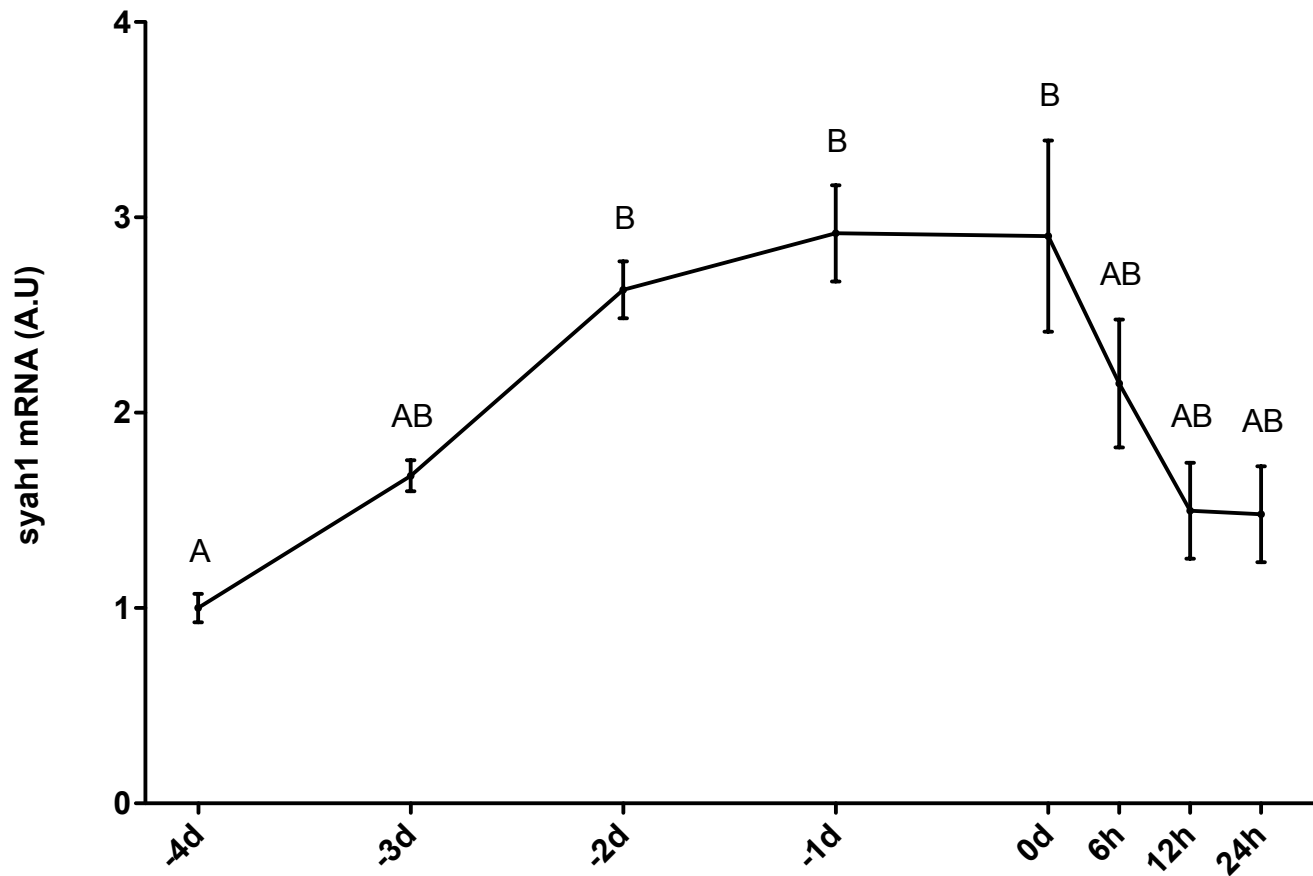

Supplement: Additional file 11: — Pacu ubiquitin specific proteases functional domains and digital gene expression. Ubiquitin specific proteases (USP) digital gene expression analysis between slow and fast skeletal muscle. Analysis between fast and slow skeletal muscle was performed using the number of reads mapped normalized by length and library size. Normalized counts for individual animals per contig are shown. Differences between tissues were analysed by t-test followed by a False Discovery Rate correction. Significant differences were considered when FDR < 0.05. Pacu, zebrafish and human protein USP functional domains were determined using InterProScan webserver. Relative gene expression was analysed for fbox25, huwe, mafbx, murf1a, murf1b, syah1, syyna, trip12, ufd2, igf1, igf2a, igf2b, igf3, igf1ra, igf1rb, usp2a, usp2b, usp4, usp5a, usp5b, usp8, usp9, usp11, usp12b, usp12b, usp14, usp16, usp19, usp21, usp24, usp28, usp30, usp36 and usp46, Values represents mean ± SE (n = 8 fish). [file 12864_2015_1423_MOESM11_ESM.zip › Supplementary_file_11_USPs_domains/Graphs_gene_expression_E3-ubiquitin_USP_IGF/syah1.pdf]

syyna mRNA (A.U)

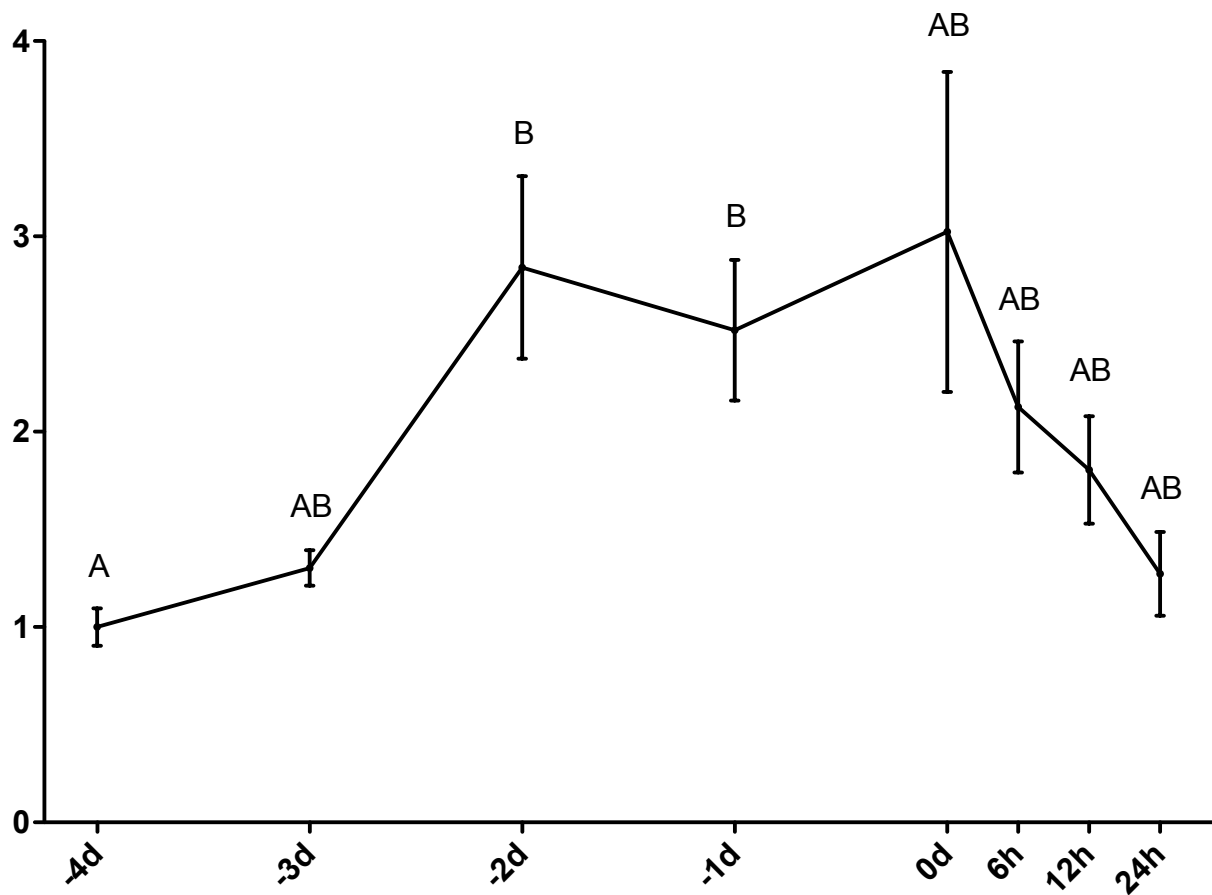

Supplement: Additional file 11: — Pacu ubiquitin specific proteases functional domains and digital gene expression. Ubiquitin specific proteases (USP) digital gene expression analysis between slow and fast skeletal muscle. Analysis between fast and slow skeletal muscle was performed using the number of reads mapped normalized by length and library size. Normalized counts for individual animals per contig are shown. Differences between tissues were analysed by t-test followed by a False Discovery Rate correction. Significant differences were considered when FDR < 0.05. Pacu, zebrafish and human protein USP functional domains were determined using InterProScan webserver. Relative gene expression was analysed for fbox25, huwe, mafbx, murf1a, murf1b, syah1, syyna, trip12, ufd2, igf1, igf2a, igf2b, igf3, igf1ra, igf1rb, usp2a, usp2b, usp4, usp5a, usp5b, usp8, usp9, usp11, usp12b, usp12b, usp14, usp16, usp19, usp21, usp24, usp28, usp30, usp36 and usp46, Values represents mean ± SE (n = 8 fish). [file 12864_2015_1423_MOESM11_ESM.zip › Supplementary_file_11_USPs_domains/Graphs_gene_expression_E3-ubiquitin_USP_IGF/syyna.pdf]

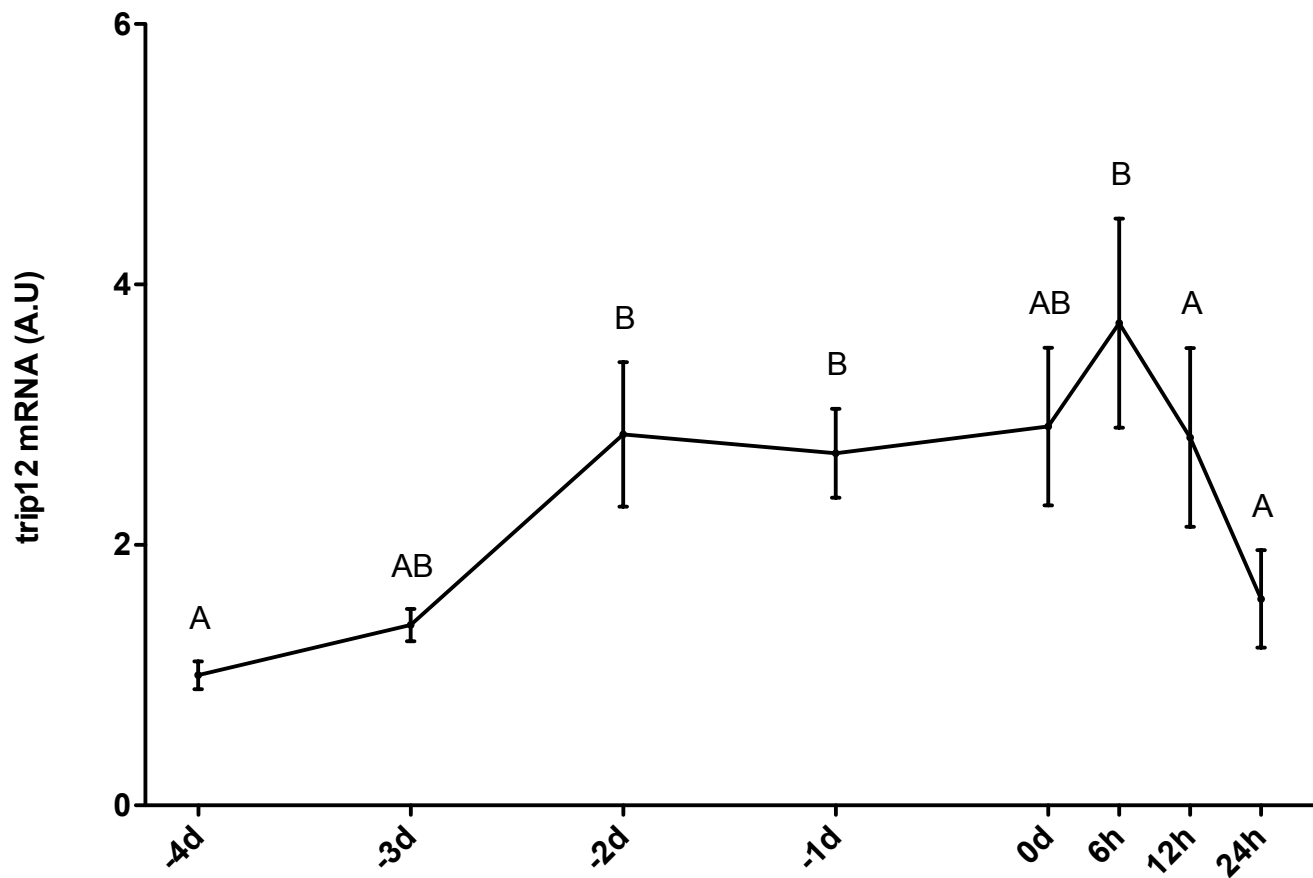

Supplement: Additional file 11: — Pacu ubiquitin specific proteases functional domains and digital gene expression. Ubiquitin specific proteases (USP) digital gene expression analysis between slow and fast skeletal muscle. Analysis between fast and slow skeletal muscle was performed using the number of reads mapped normalized by length and library size. Normalized counts for individual animals per contig are shown. Differences between tissues were analysed by t-test followed by a False Discovery Rate correction. Significant differences were considered when FDR < 0.05. Pacu, zebrafish and human protein USP functional domains were determined using InterProScan webserver. Relative gene expression was analysed for fbox25, huwe, mafbx, murf1a, murf1b, syah1, syyna, trip12, ufd2, igf1, igf2a, igf2b, igf3, igf1ra, igf1rb, usp2a, usp2b, usp4, usp5a, usp5b, usp8, usp9, usp11, usp12b, usp12b, usp14, usp16, usp19, usp21, usp24, usp28, usp30, usp36 and usp46, Values represents mean ± SE (n = 8 fish). [file 12864_2015_1423_MOESM11_ESM.zip › Supplementary_file_11_USPs_domains/Graphs_gene_expression_E3-ubiquitin_USP_IGF/trip12.pdf]

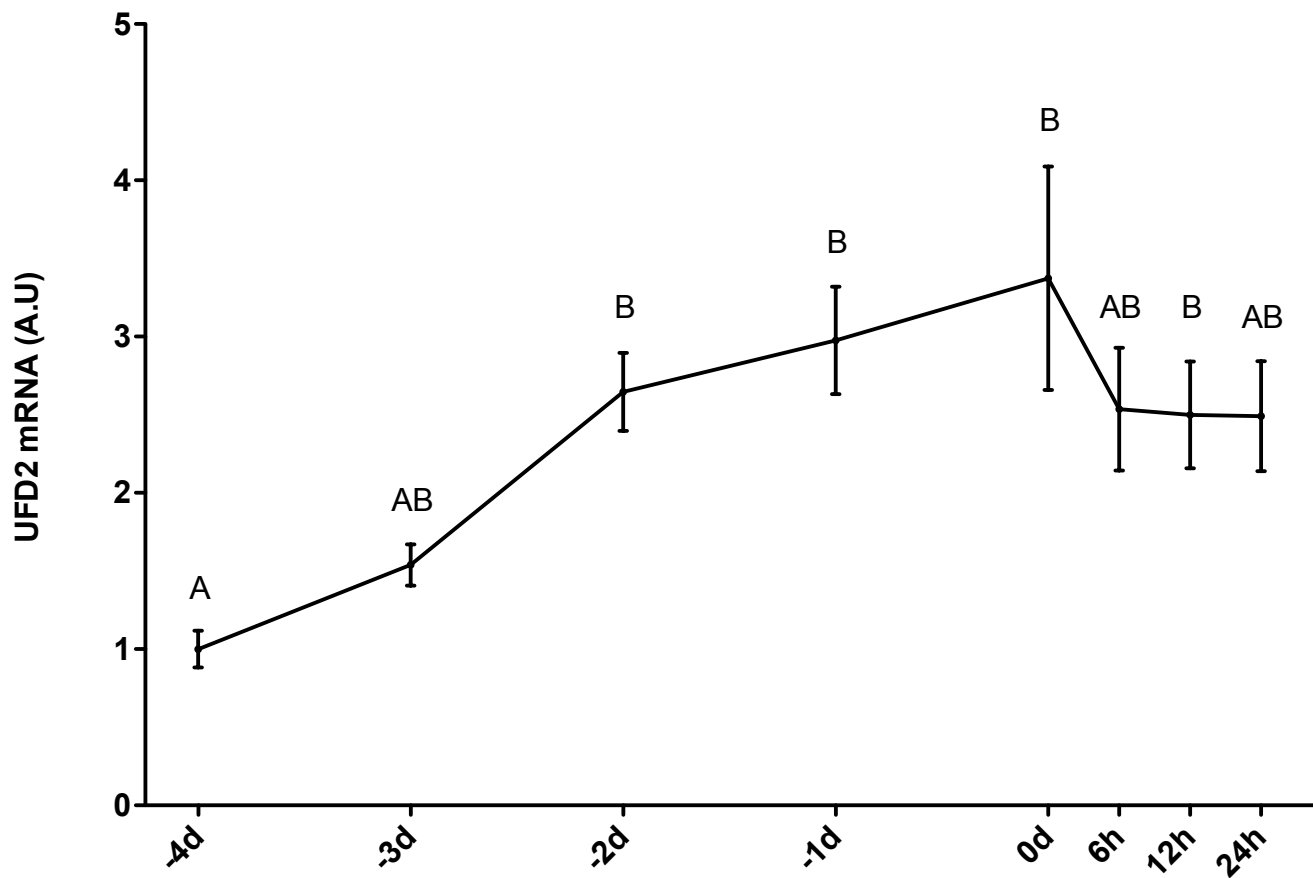

Supplement: Additional file 11: — Pacu ubiquitin specific proteases functional domains and digital gene expression. Ubiquitin specific proteases (USP) digital gene expression analysis between slow and fast skeletal muscle. Analysis between fast and slow skeletal muscle was performed using the number of reads mapped normalized by length and library size. Normalized counts for individual animals per contig are shown. Differences between tissues were analysed by t-test followed by a False Discovery Rate correction. Significant differences were considered when FDR < 0.05. Pacu, zebrafish and human protein USP functional domains were determined using InterProScan webserver. Relative gene expression was analysed for fbox25, huwe, mafbx, murf1a, murf1b, syah1, syyna, trip12, ufd2, igf1, igf2a, igf2b, igf3, igf1ra, igf1rb, usp2a, usp2b, usp4, usp5a, usp5b, usp8, usp9, usp11, usp12b, usp12b, usp14, usp16, usp19, usp21, usp24, usp28, usp30, usp36 and usp46, Values represents mean ± SE (n = 8 fish). [file 12864_2015_1423_MOESM11_ESM.zip › Supplementary_file_11_USPs_domains/Graphs_gene_expression_E3-ubiquitin_USP_IGF/UFD2.pdf]

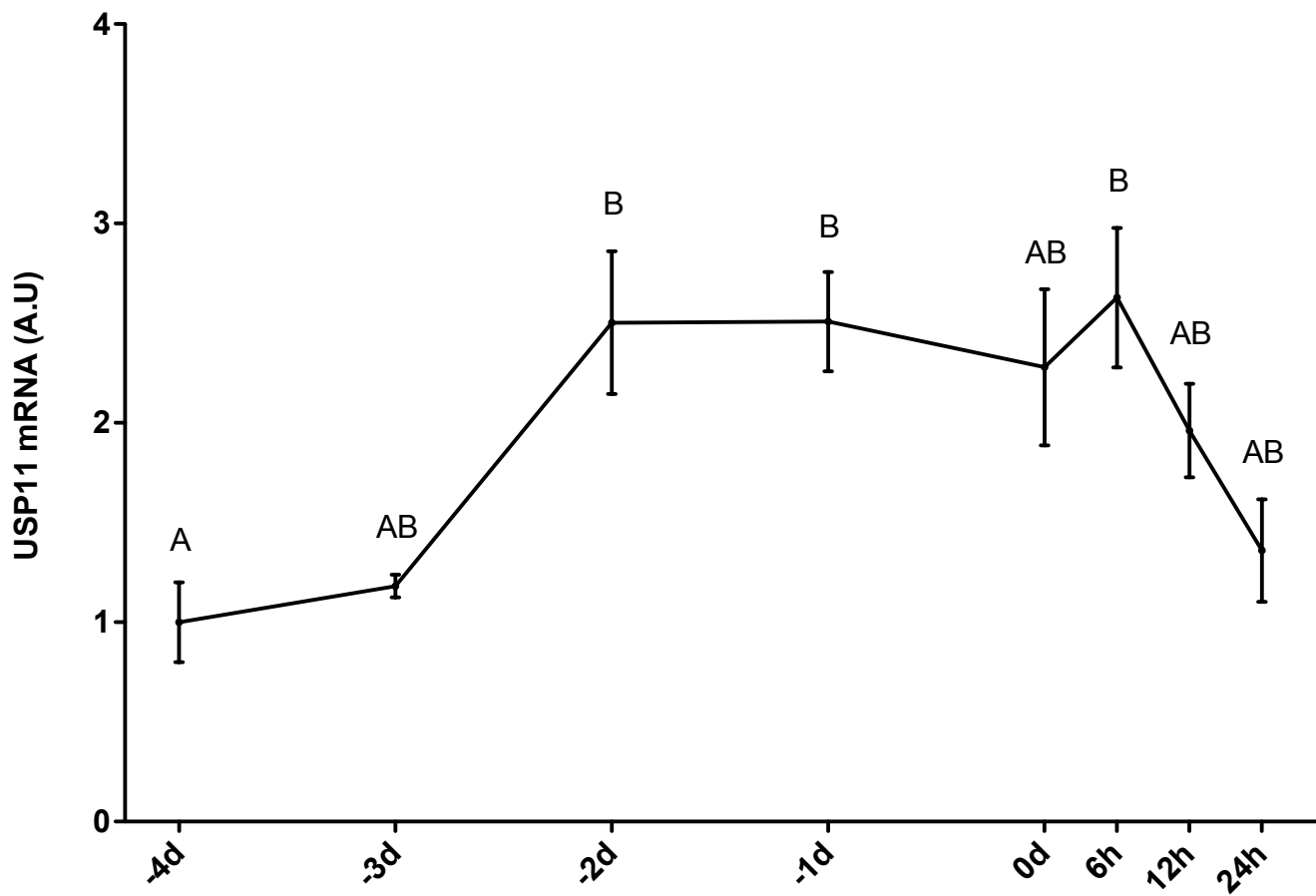

Supplement: Additional file 11: — Pacu ubiquitin specific proteases functional domains and digital gene expression. Ubiquitin specific proteases (USP) digital gene expression analysis between slow and fast skeletal muscle. Analysis between fast and slow skeletal muscle was performed using the number of reads mapped normalized by length and library size. Normalized counts for individual animals per contig are shown. Differences between tissues were analysed by t-test followed by a False Discovery Rate correction. Significant differences were considered when FDR < 0.05. Pacu, zebrafish and human protein USP functional domains were determined using InterProScan webserver. Relative gene expression was analysed for fbox25, huwe, mafbx, murf1a, murf1b, syah1, syyna, trip12, ufd2, igf1, igf2a, igf2b, igf3, igf1ra, igf1rb, usp2a, usp2b, usp4, usp5a, usp5b, usp8, usp9, usp11, usp12b, usp12b, usp14, usp16, usp19, usp21, usp24, usp28, usp30, usp36 and usp46, Values represents mean ± SE (n = 8 fish). [file 12864_2015_1423_MOESM11_ESM.zip › Supplementary_file_11_USPs_domains/Graphs_gene_expression_E3-ubiquitin_USP_IGF/USP11.pdf]

USP12a mRNA (A.U)

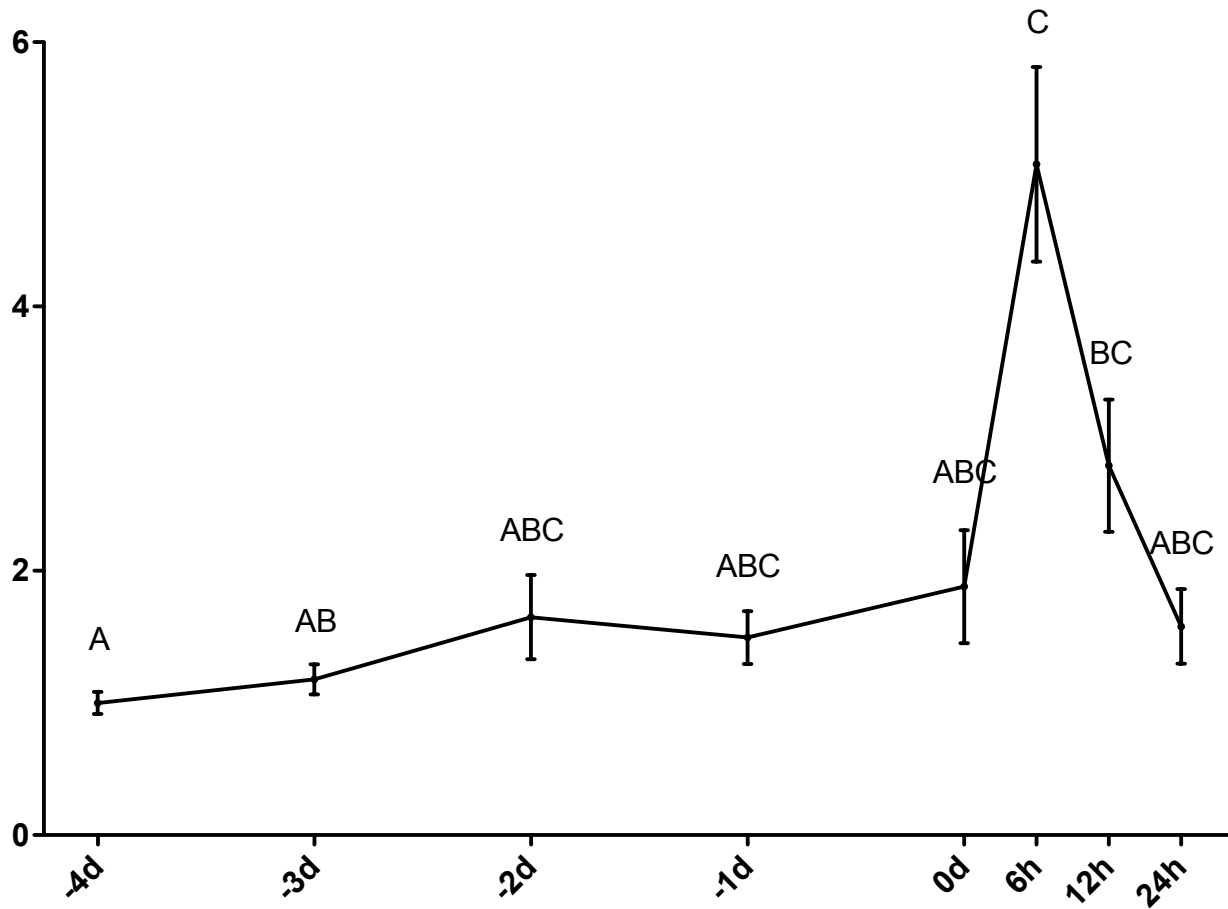

Supplement: Additional file 11: — Pacu ubiquitin specific proteases functional domains and digital gene expression. Ubiquitin specific proteases (USP) digital gene expression analysis between slow and fast skeletal muscle. Analysis between fast and slow skeletal muscle was performed using the number of reads mapped normalized by length and library size. Normalized counts for individual animals per contig are shown. Differences between tissues were analysed by t-test followed by a False Discovery Rate correction. Significant differences were considered when FDR < 0.05. Pacu, zebrafish and human protein USP functional domains were determined using InterProScan webserver. Relative gene expression was analysed for fbox25, huwe, mafbx, murf1a, murf1b, syah1, syyna, trip12, ufd2, igf1, igf2a, igf2b, igf3, igf1ra, igf1rb, usp2a, usp2b, usp4, usp5a, usp5b, usp8, usp9, usp11, usp12b, usp12b, usp14, usp16, usp19, usp21, usp24, usp28, usp30, usp36 and usp46, Values represents mean ± SE (n = 8 fish). [file 12864_2015_1423_MOESM11_ESM.zip › Supplementary_file_11_USPs_domains/Graphs_gene_expression_E3-ubiquitin_USP_IGF/USP12a.pdf]

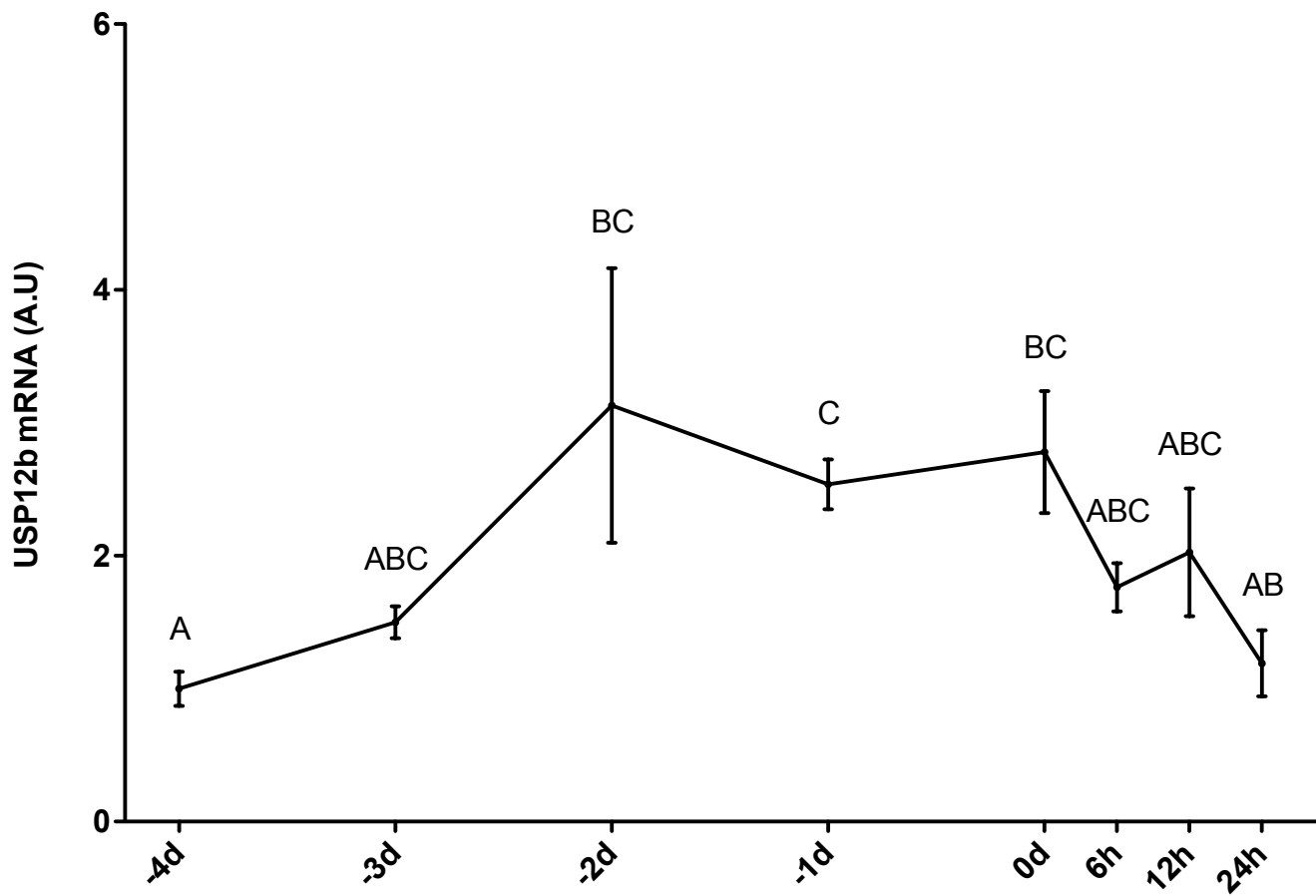

Supplement: Additional file 11: — Pacu ubiquitin specific proteases functional domains and digital gene expression. Ubiquitin specific proteases (USP) digital gene expression analysis between slow and fast skeletal muscle. Analysis between fast and slow skeletal muscle was performed using the number of reads mapped normalized by length and library size. Normalized counts for individual animals per contig are shown. Differences between tissues were analysed by t-test followed by a False Discovery Rate correction. Significant differences were considered when FDR < 0.05. Pacu, zebrafish and human protein USP functional domains were determined using InterProScan webserver. Relative gene expression was analysed for fbox25, huwe, mafbx, murf1a, murf1b, syah1, syyna, trip12, ufd2, igf1, igf2a, igf2b, igf3, igf1ra, igf1rb, usp2a, usp2b, usp4, usp5a, usp5b, usp8, usp9, usp11, usp12b, usp12b, usp14, usp16, usp19, usp21, usp24, usp28, usp30, usp36 and usp46, Values represents mean ± SE (n = 8 fish). [file 12864_2015_1423_MOESM11_ESM.zip › Supplementary_file_11_USPs_domains/Graphs_gene_expression_E3-ubiquitin_USP_IGF/USP12b.pdf]

USP14 mRNA (A.U)

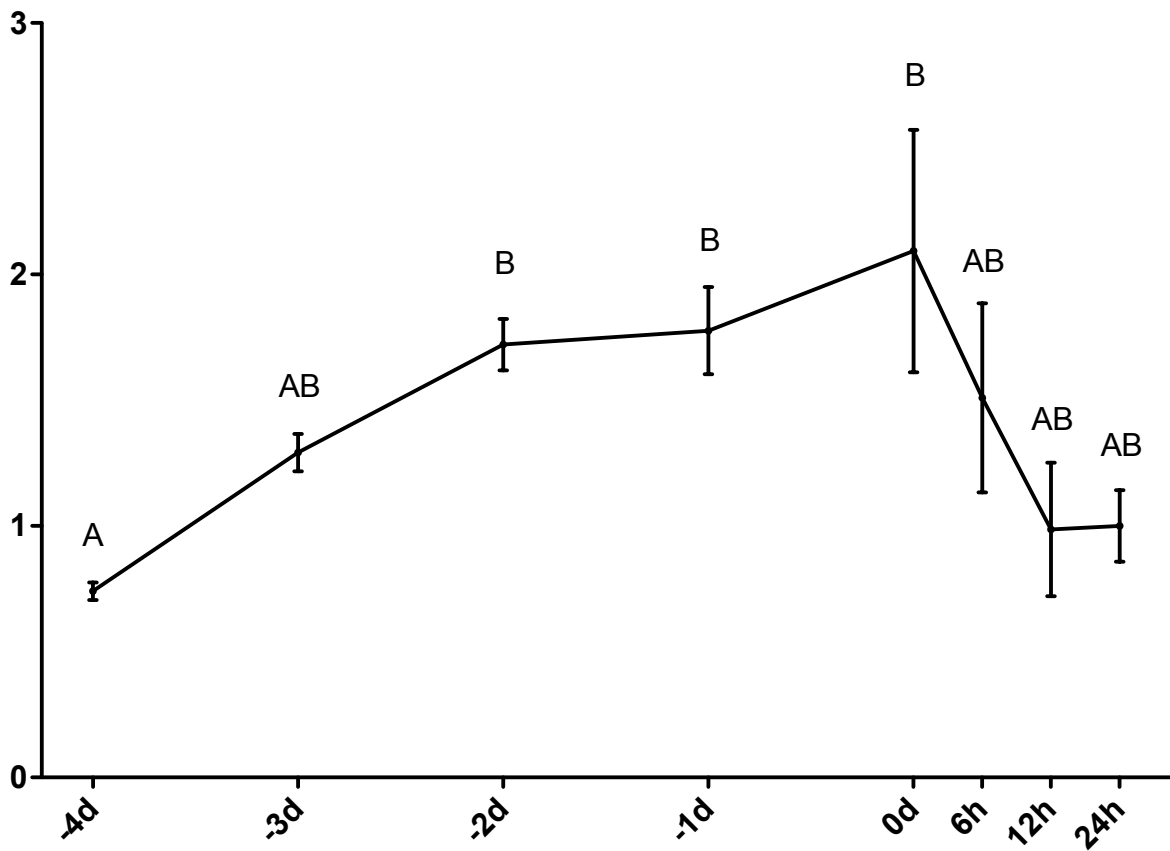

Supplement: Additional file 11: — Pacu ubiquitin specific proteases functional domains and digital gene expression. Ubiquitin specific proteases (USP) digital gene expression analysis between slow and fast skeletal muscle. Analysis between fast and slow skeletal muscle was performed using the number of reads mapped normalized by length and library size. Normalized counts for individual animals per contig are shown. Differences between tissues were analysed by t-test followed by a False Discovery Rate correction. Significant differences were considered when FDR < 0.05. Pacu, zebrafish and human protein USP functional domains were determined using InterProScan webserver. Relative gene expression was analysed for fbox25, huwe, mafbx, murf1a, murf1b, syah1, syyna, trip12, ufd2, igf1, igf2a, igf2b, igf3, igf1ra, igf1rb, usp2a, usp2b, usp4, usp5a, usp5b, usp8, usp9, usp11, usp12b, usp12b, usp14, usp16, usp19, usp21, usp24, usp28, usp30, usp36 and usp46, Values represents mean ± SE (n = 8 fish). [file 12864_2015_1423_MOESM11_ESM.zip › Supplementary_file_11_USPs_domains/Graphs_gene_expression_E3-ubiquitin_USP_IGF/USP14.pdf]

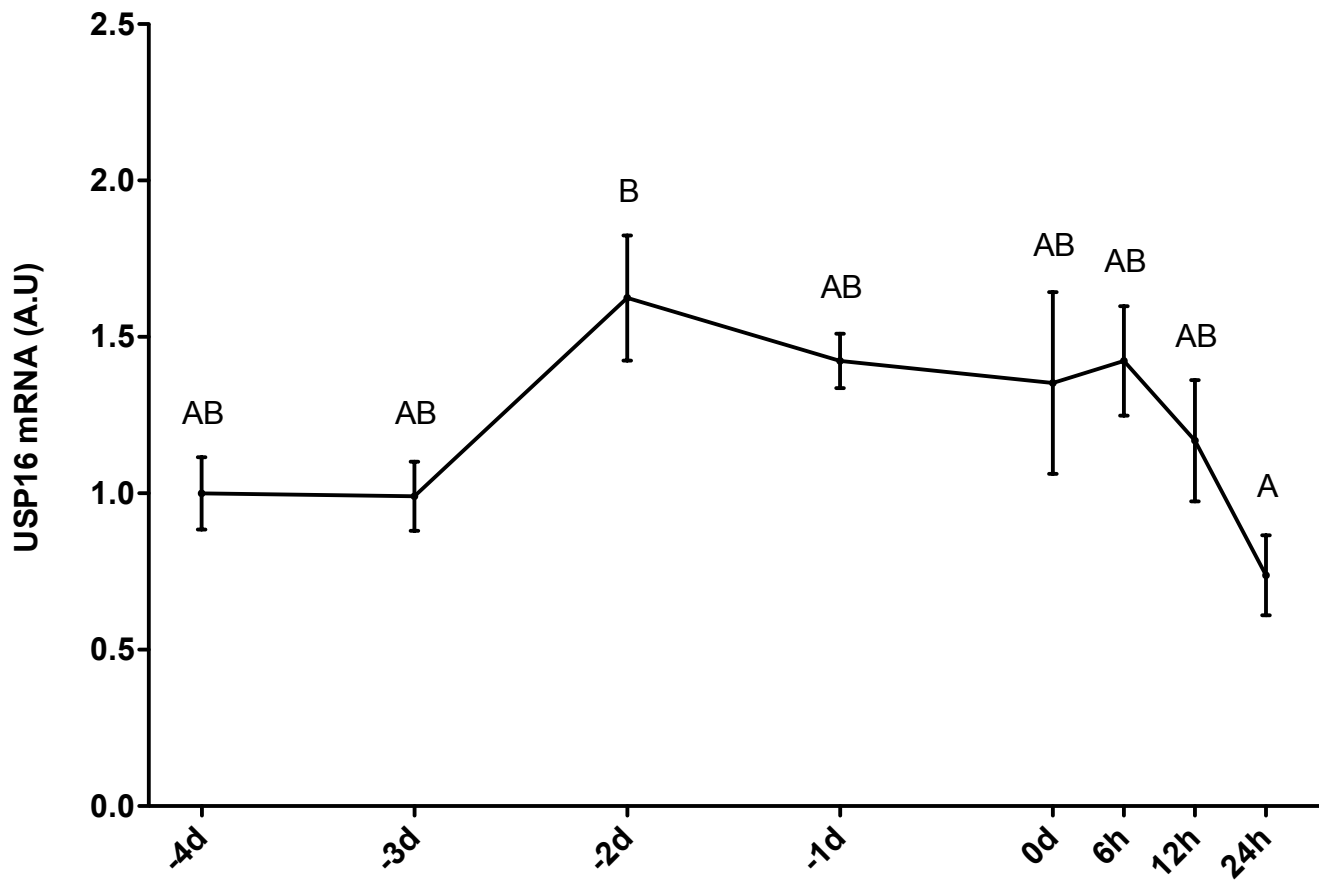

Supplement: Additional file 11: — Pacu ubiquitin specific proteases functional domains and digital gene expression. Ubiquitin specific proteases (USP) digital gene expression analysis between slow and fast skeletal muscle. Analysis between fast and slow skeletal muscle was performed using the number of reads mapped normalized by length and library size. Normalized counts for individual animals per contig are shown. Differences between tissues were analysed by t-test followed by a False Discovery Rate correction. Significant differences were considered when FDR < 0.05. Pacu, zebrafish and human protein USP functional domains were determined using InterProScan webserver. Relative gene expression was analysed for fbox25, huwe, mafbx, murf1a, murf1b, syah1, syyna, trip12, ufd2, igf1, igf2a, igf2b, igf3, igf1ra, igf1rb, usp2a, usp2b, usp4, usp5a, usp5b, usp8, usp9, usp11, usp12b, usp12b, usp14, usp16, usp19, usp21, usp24, usp28, usp30, usp36 and usp46, Values represents mean ± SE (n = 8 fish). [file 12864_2015_1423_MOESM11_ESM.zip › Supplementary_file_11_USPs_domains/Graphs_gene_expression_E3-ubiquitin_USP_IGF/USP16.pdf]

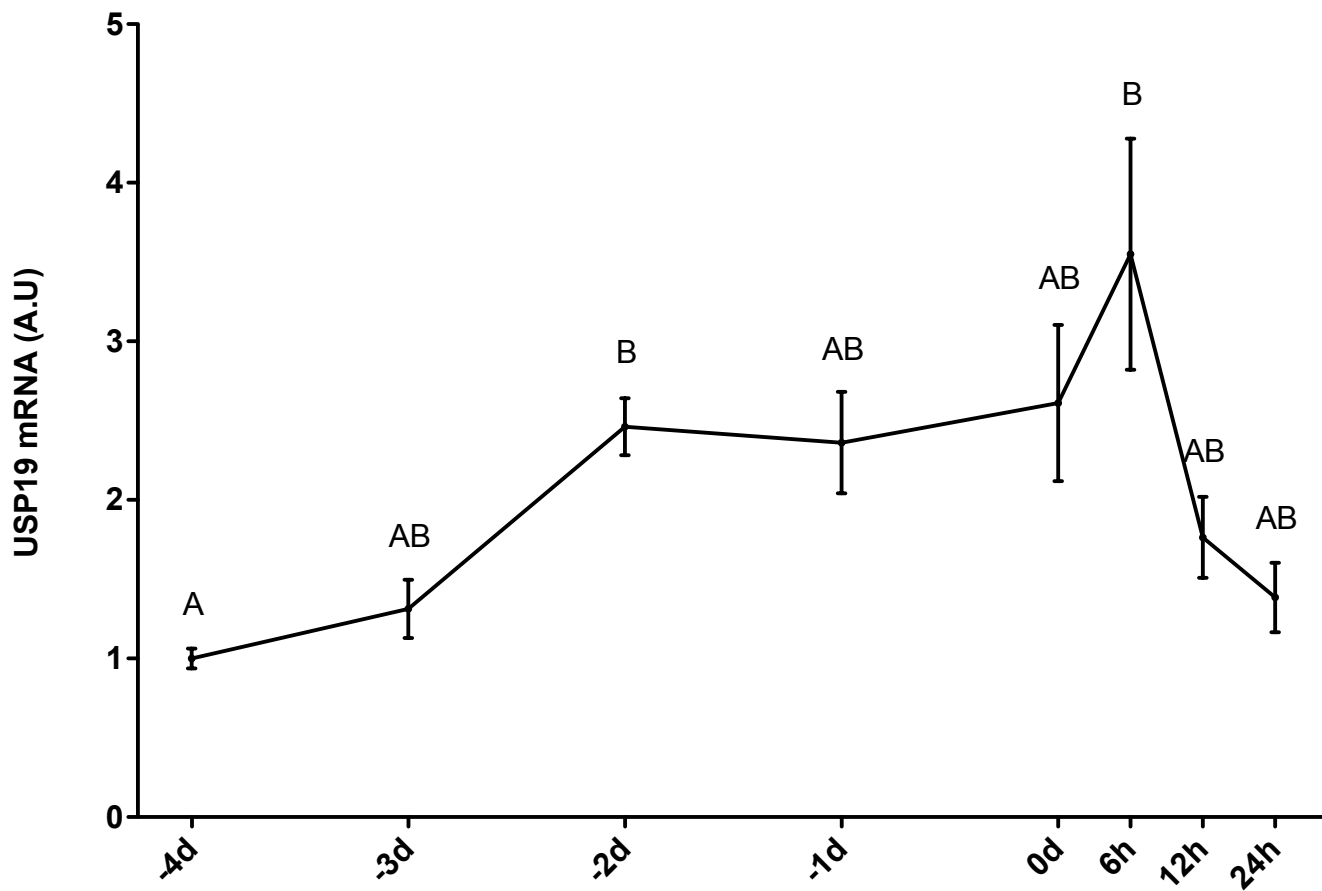

Supplement: Additional file 11: — Pacu ubiquitin specific proteases functional domains and digital gene expression. Ubiquitin specific proteases (USP) digital gene expression analysis between slow and fast skeletal muscle. Analysis between fast and slow skeletal muscle was performed using the number of reads mapped normalized by length and library size. Normalized counts for individual animals per contig are shown. Differences between tissues were analysed by t-test followed by a False Discovery Rate correction. Significant differences were considered when FDR < 0.05. Pacu, zebrafish and human protein USP functional domains were determined using InterProScan webserver. Relative gene expression was analysed for fbox25, huwe, mafbx, murf1a, murf1b, syah1, syyna, trip12, ufd2, igf1, igf2a, igf2b, igf3, igf1ra, igf1rb, usp2a, usp2b, usp4, usp5a, usp5b, usp8, usp9, usp11, usp12b, usp12b, usp14, usp16, usp19, usp21, usp24, usp28, usp30, usp36 and usp46, Values represents mean ± SE (n = 8 fish). [file 12864_2015_1423_MOESM11_ESM.zip › Supplementary_file_11_USPs_domains/Graphs_gene_expression_E3-ubiquitin_USP_IGF/USP19.pdf]

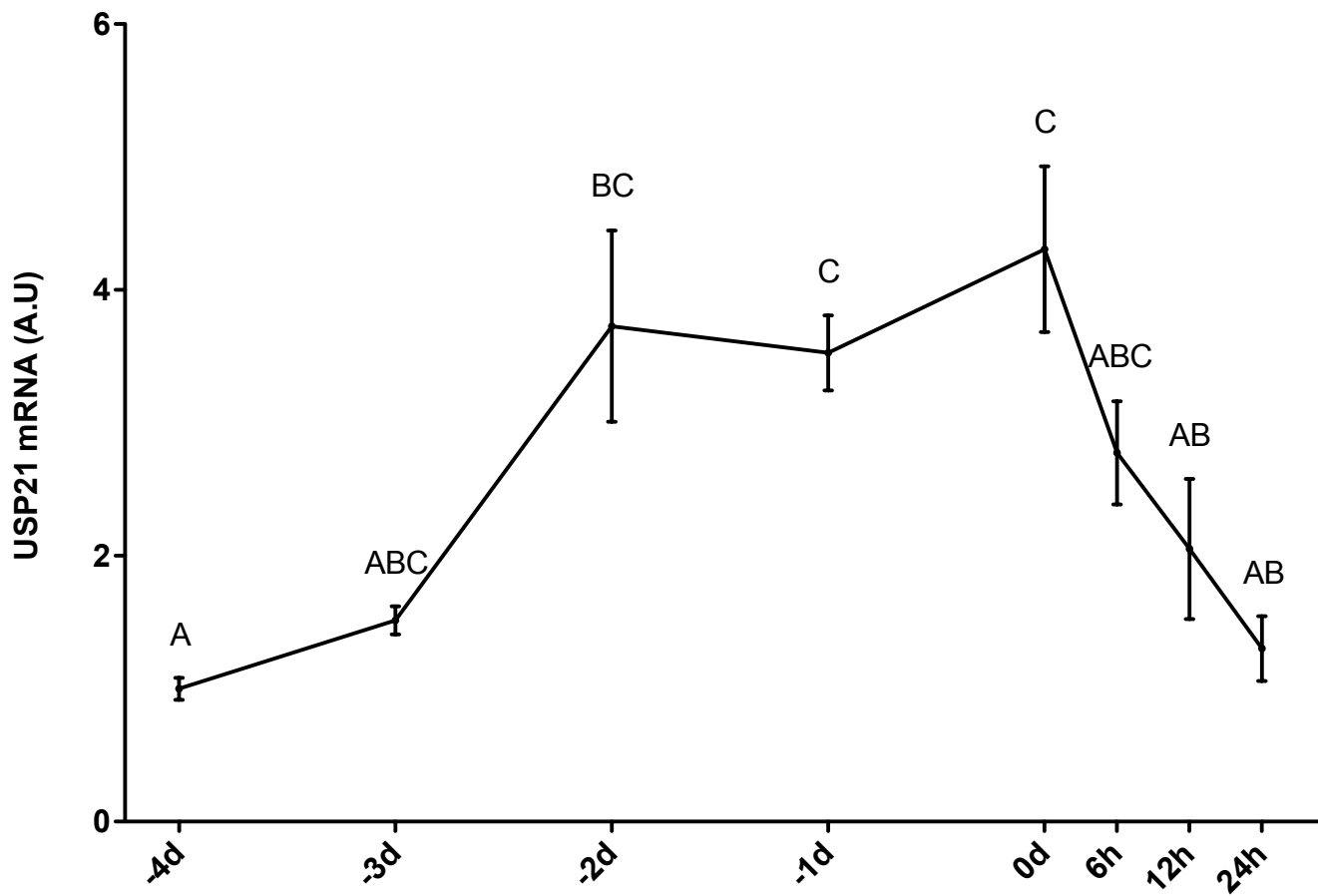

Supplement: Additional file 11: — Pacu ubiquitin specific proteases functional domains and digital gene expression. Ubiquitin specific proteases (USP) digital gene expression analysis between slow and fast skeletal muscle. Analysis between fast and slow skeletal muscle was performed using the number of reads mapped normalized by length and library size. Normalized counts for individual animals per contig are shown. Differences between tissues were analysed by t-test followed by a False Discovery Rate correction. Significant differences were considered when FDR < 0.05. Pacu, zebrafish and human protein USP functional domains were determined using InterProScan webserver. Relative gene expression was analysed for fbox25, huwe, mafbx, murf1a, murf1b, syah1, syyna, trip12, ufd2, igf1, igf2a, igf2b, igf3, igf1ra, igf1rb, usp2a, usp2b, usp4, usp5a, usp5b, usp8, usp9, usp11, usp12b, usp12b, usp14, usp16, usp19, usp21, usp24, usp28, usp30, usp36 and usp46, Values represents mean ± SE (n = 8 fish). [file 12864_2015_1423_MOESM11_ESM.zip › Supplementary_file_11_USPs_domains/Graphs_gene_expression_E3-ubiquitin_USP_IGF/USP21.pdf]

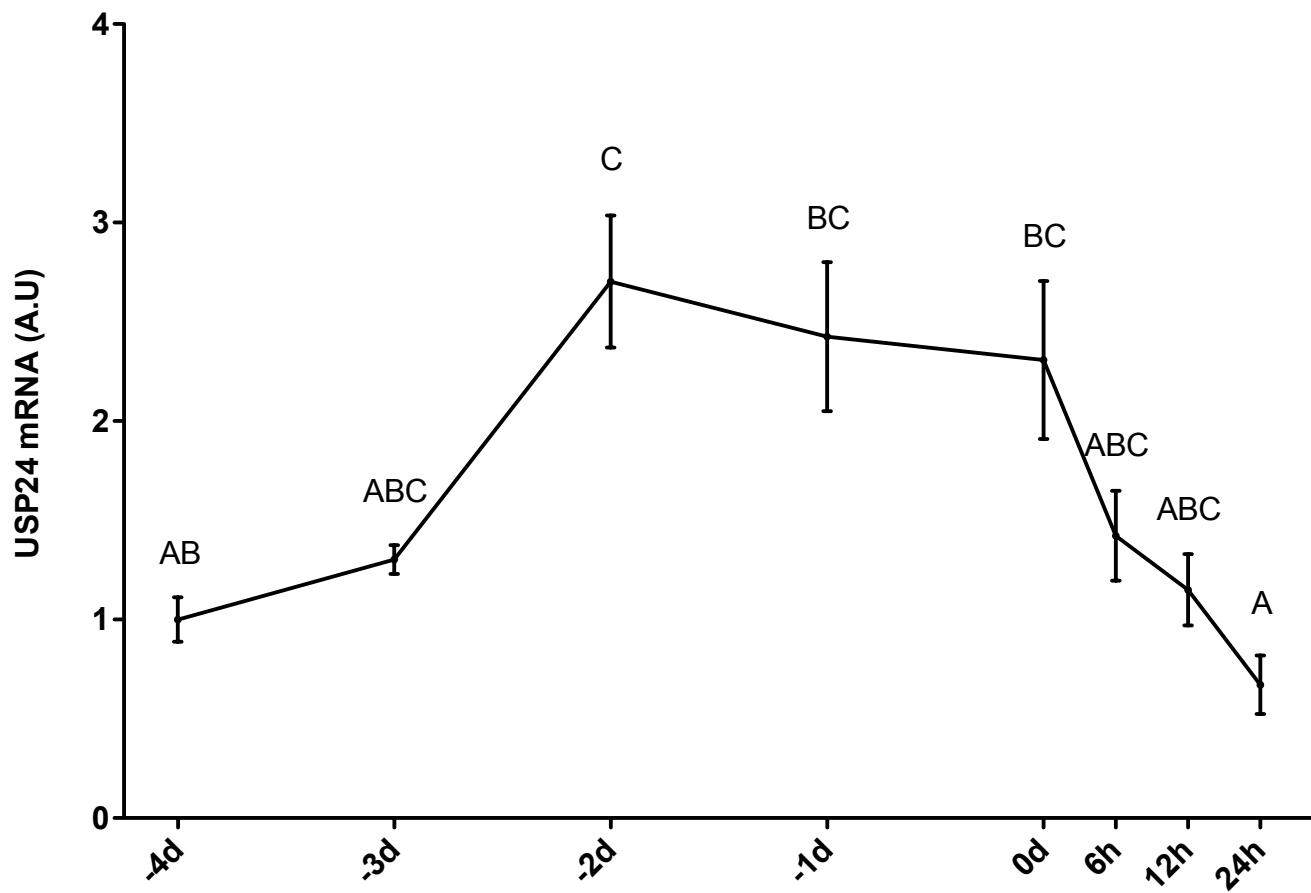

Supplement: Additional file 11: — Pacu ubiquitin specific proteases functional domains and digital gene expression. Ubiquitin specific proteases (USP) digital gene expression analysis between slow and fast skeletal muscle. Analysis between fast and slow skeletal muscle was performed using the number of reads mapped normalized by length and library size. Normalized counts for individual animals per contig are shown. Differences between tissues were analysed by t-test followed by a False Discovery Rate correction. Significant differences were considered when FDR < 0.05. Pacu, zebrafish and human protein USP functional domains were determined using InterProScan webserver. Relative gene expression was analysed for fbox25, huwe, mafbx, murf1a, murf1b, syah1, syyna, trip12, ufd2, igf1, igf2a, igf2b, igf3, igf1ra, igf1rb, usp2a, usp2b, usp4, usp5a, usp5b, usp8, usp9, usp11, usp12b, usp12b, usp14, usp16, usp19, usp21, usp24, usp28, usp30, usp36 and usp46, Values represents mean ± SE (n = 8 fish). [file 12864_2015_1423_MOESM11_ESM.zip › Supplementary_file_11_USPs_domains/Graphs_gene_expression_E3-ubiquitin_USP_IGF/USP24.pdf]

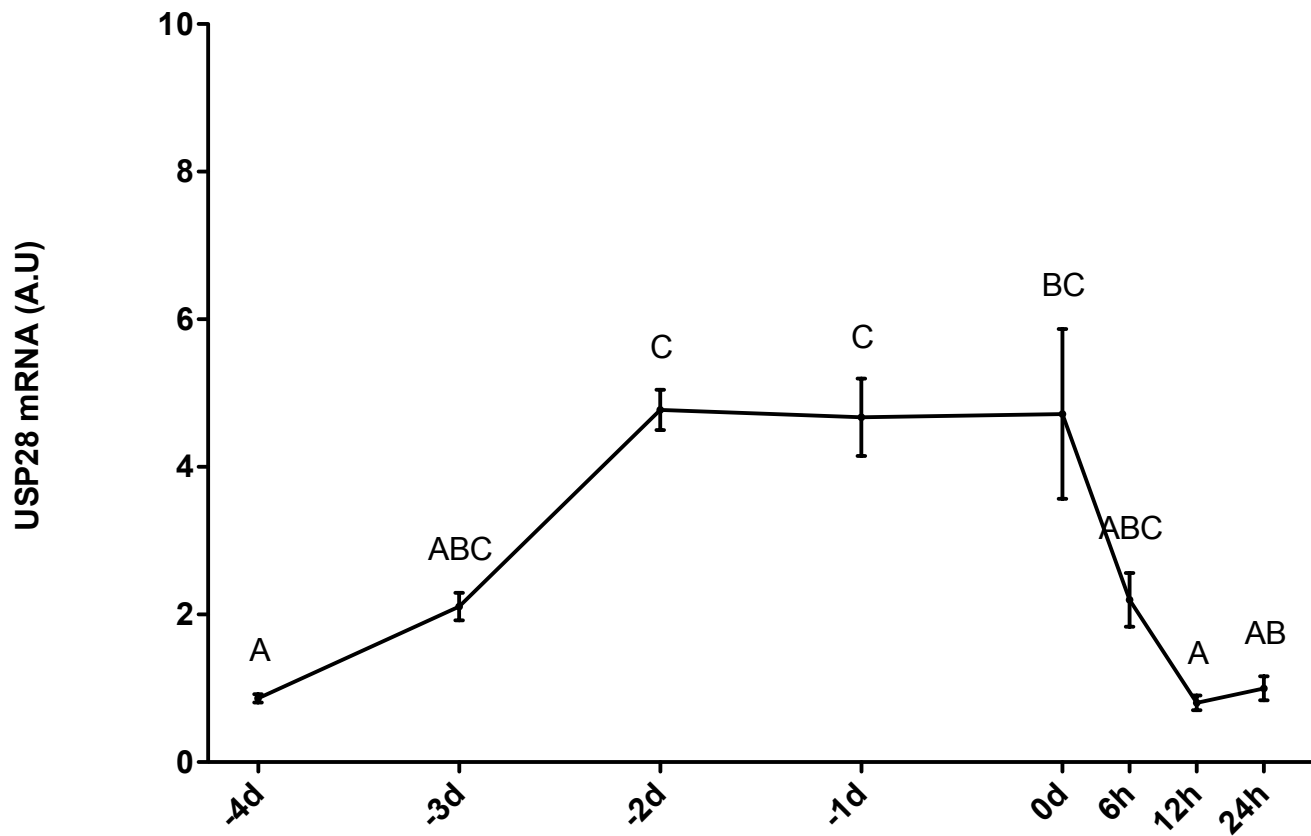

Supplement: Additional file 11: — Pacu ubiquitin specific proteases functional domains and digital gene expression. Ubiquitin specific proteases (USP) digital gene expression analysis between slow and fast skeletal muscle. Analysis between fast and slow skeletal muscle was performed using the number of reads mapped normalized by length and library size. Normalized counts for individual animals per contig are shown. Differences between tissues were analysed by t-test followed by a False Discovery Rate correction. Significant differences were considered when FDR < 0.05. Pacu, zebrafish and human protein USP functional domains were determined using InterProScan webserver. Relative gene expression was analysed for fbox25, huwe, mafbx, murf1a, murf1b, syah1, syyna, trip12, ufd2, igf1, igf2a, igf2b, igf3, igf1ra, igf1rb, usp2a, usp2b, usp4, usp5a, usp5b, usp8, usp9, usp11, usp12b, usp12b, usp14, usp16, usp19, usp21, usp24, usp28, usp30, usp36 and usp46, Values represents mean ± SE (n = 8 fish). [file 12864_2015_1423_MOESM11_ESM.zip › Supplementary_file_11_USPs_domains/Graphs_gene_expression_E3-ubiquitin_USP_IGF/USP28.pdf]

USP2a mRNA (A.U)

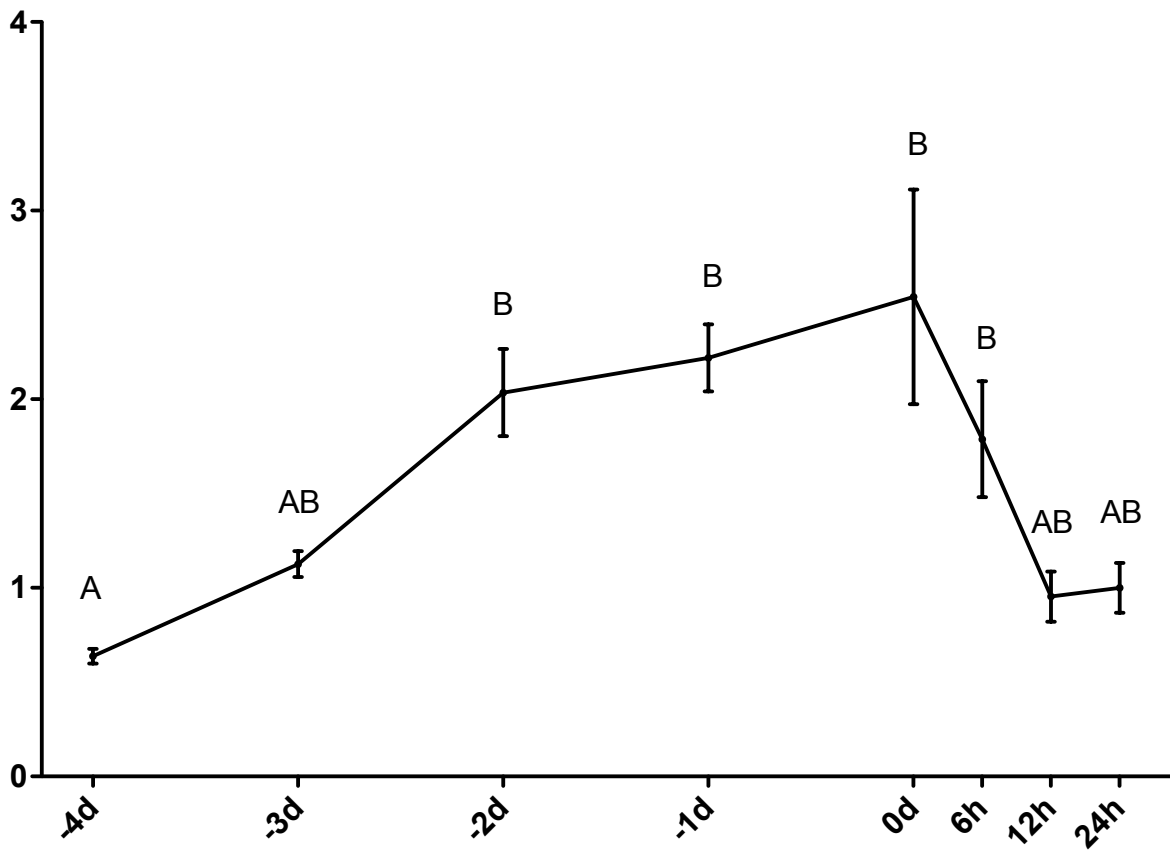

Supplement: Additional file 11: — Pacu ubiquitin specific proteases functional domains and digital gene expression. Ubiquitin specific proteases (USP) digital gene expression analysis between slow and fast skeletal muscle. Analysis between fast and slow skeletal muscle was performed using the number of reads mapped normalized by length and library size. Normalized counts for individual animals per contig are shown. Differences between tissues were analysed by t-test followed by a False Discovery Rate correction. Significant differences were considered when FDR < 0.05. Pacu, zebrafish and human protein USP functional domains were determined using InterProScan webserver. Relative gene expression was analysed for fbox25, huwe, mafbx, murf1a, murf1b, syah1, syyna, trip12, ufd2, igf1, igf2a, igf2b, igf3, igf1ra, igf1rb, usp2a, usp2b, usp4, usp5a, usp5b, usp8, usp9, usp11, usp12b, usp12b, usp14, usp16, usp19, usp21, usp24, usp28, usp30, usp36 and usp46, Values represents mean ± SE (n = 8 fish). [file 12864_2015_1423_MOESM11_ESM.zip › Supplementary_file_11_USPs_domains/Graphs_gene_expression_E3-ubiquitin_USP_IGF/USP2a.pdf]

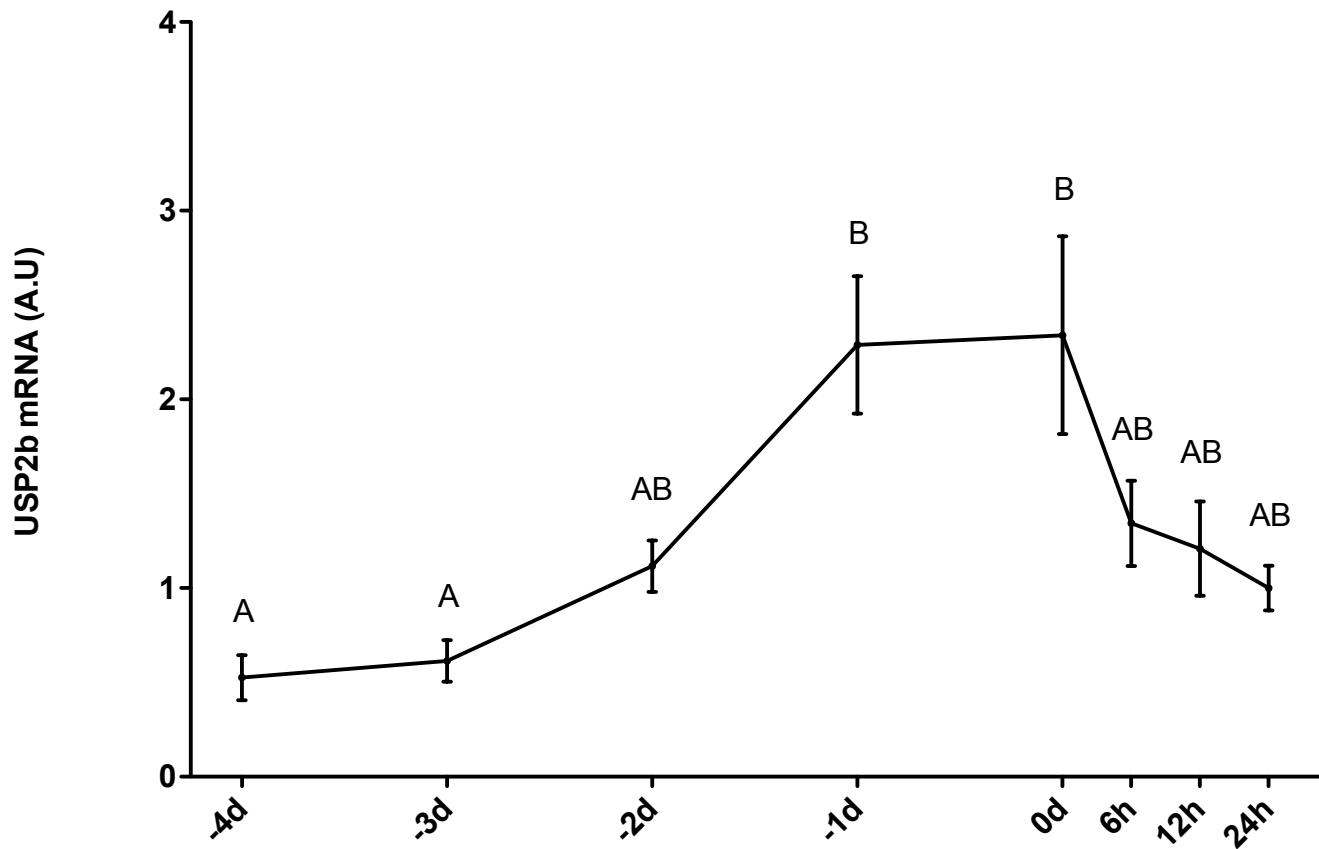

Supplement: Additional file 11: — Pacu ubiquitin specific proteases functional domains and digital gene expression. Ubiquitin specific proteases (USP) digital gene expression analysis between slow and fast skeletal muscle. Analysis between fast and slow skeletal muscle was performed using the number of reads mapped normalized by length and library size. Normalized counts for individual animals per contig are shown. Differences between tissues were analysed by t-test followed by a False Discovery Rate correction. Significant differences were considered when FDR < 0.05. Pacu, zebrafish and human protein USP functional domains were determined using InterProScan webserver. Relative gene expression was analysed for fbox25, huwe, mafbx, murf1a, murf1b, syah1, syyna, trip12, ufd2, igf1, igf2a, igf2b, igf3, igf1ra, igf1rb, usp2a, usp2b, usp4, usp5a, usp5b, usp8, usp9, usp11, usp12b, usp12b, usp14, usp16, usp19, usp21, usp24, usp28, usp30, usp36 and usp46, Values represents mean ± SE (n = 8 fish). [file 12864_2015_1423_MOESM11_ESM.zip › Supplementary_file_11_USPs_domains/Graphs_gene_expression_E3-ubiquitin_USP_IGF/USP2b.pdf]

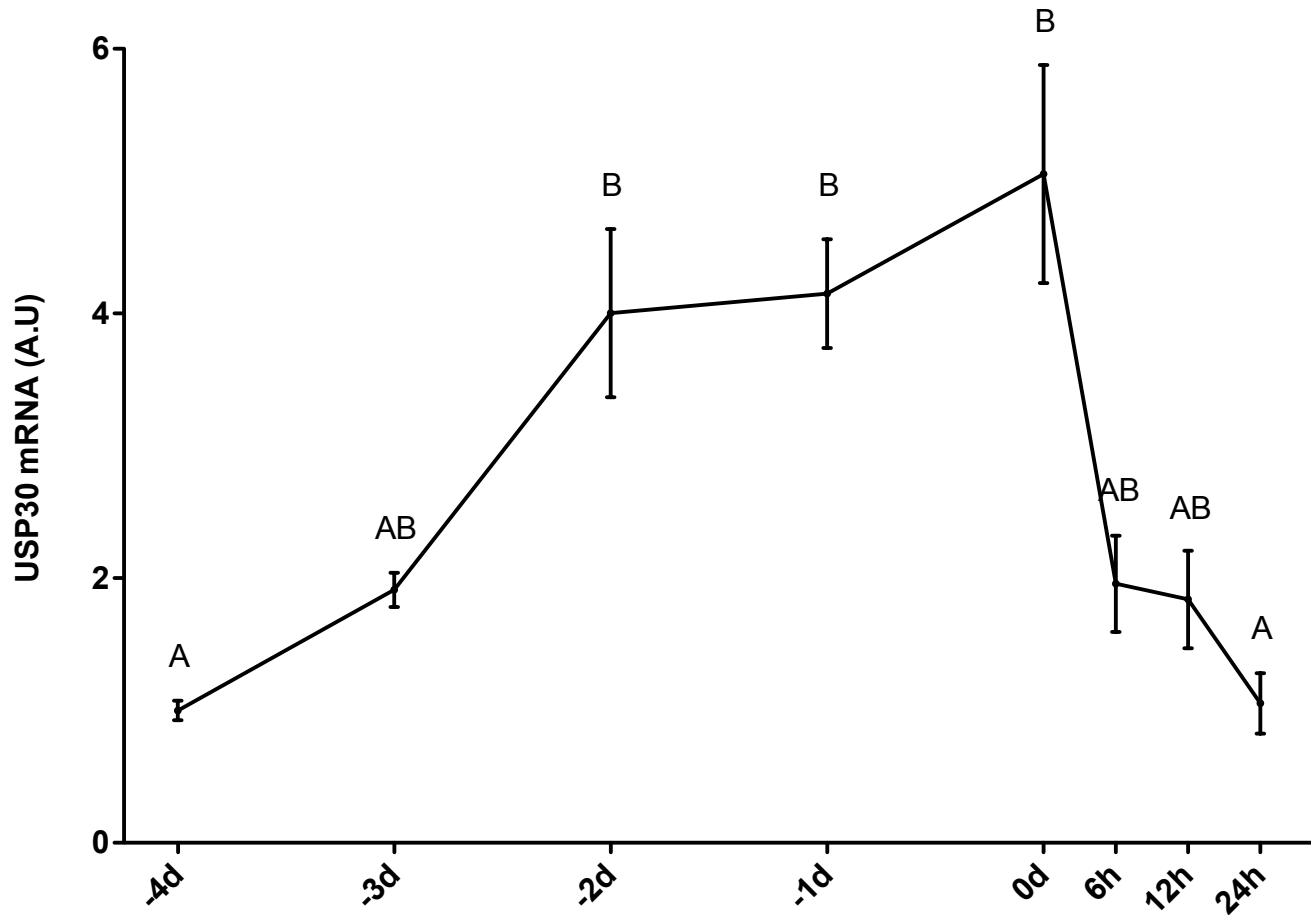

Supplement: Additional file 11: — Pacu ubiquitin specific proteases functional domains and digital gene expression. Ubiquitin specific proteases (USP) digital gene expression analysis between slow and fast skeletal muscle. Analysis between fast and slow skeletal muscle was performed using the number of reads mapped normalized by length and library size. Normalized counts for individual animals per contig are shown. Differences between tissues were analysed by t-test followed by a False Discovery Rate correction. Significant differences were considered when FDR < 0.05. Pacu, zebrafish and human protein USP functional domains were determined using InterProScan webserver. Relative gene expression was analysed for fbox25, huwe, mafbx, murf1a, murf1b, syah1, syyna, trip12, ufd2, igf1, igf2a, igf2b, igf3, igf1ra, igf1rb, usp2a, usp2b, usp4, usp5a, usp5b, usp8, usp9, usp11, usp12b, usp12b, usp14, usp16, usp19, usp21, usp24, usp28, usp30, usp36 and usp46, Values represents mean ± SE (n = 8 fish). [file 12864_2015_1423_MOESM11_ESM.zip › Supplementary_file_11_USPs_domains/Graphs_gene_expression_E3-ubiquitin_USP_IGF/USP30.pdf]

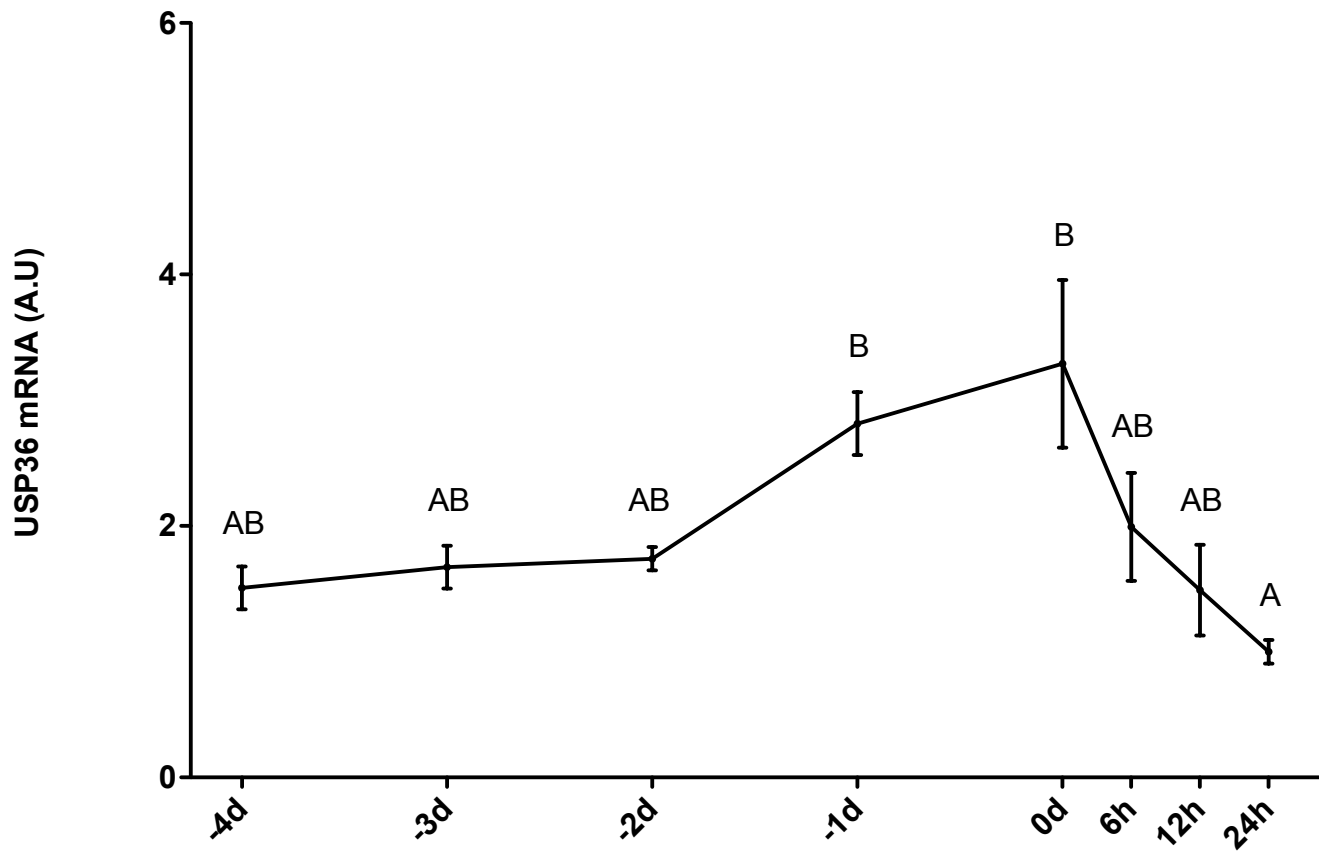

Supplement: Additional file 11: — Pacu ubiquitin specific proteases functional domains and digital gene expression. Ubiquitin specific proteases (USP) digital gene expression analysis between slow and fast skeletal muscle. Analysis between fast and slow skeletal muscle was performed using the number of reads mapped normalized by length and library size. Normalized counts for individual animals per contig are shown. Differences between tissues were analysed by t-test followed by a False Discovery Rate correction. Significant differences were considered when FDR < 0.05. Pacu, zebrafish and human protein USP functional domains were determined using InterProScan webserver. Relative gene expression was analysed for fbox25, huwe, mafbx, murf1a, murf1b, syah1, syyna, trip12, ufd2, igf1, igf2a, igf2b, igf3, igf1ra, igf1rb, usp2a, usp2b, usp4, usp5a, usp5b, usp8, usp9, usp11, usp12b, usp12b, usp14, usp16, usp19, usp21, usp24, usp28, usp30, usp36 and usp46, Values represents mean ± SE (n = 8 fish). [file 12864_2015_1423_MOESM11_ESM.zip › Supplementary_file_11_USPs_domains/Graphs_gene_expression_E3-ubiquitin_USP_IGF/USP36.pdf]

USP4 mRNA (A.U)

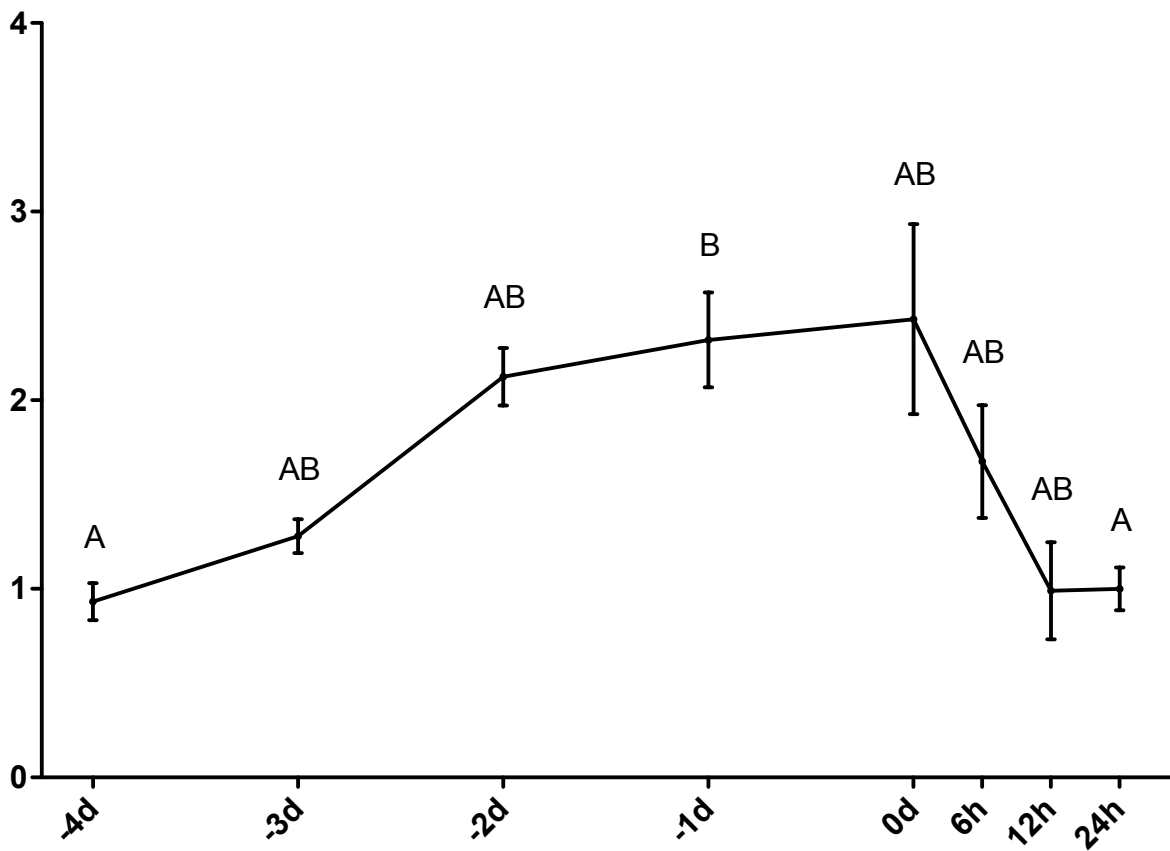

Supplement: Additional file 11: — Pacu ubiquitin specific proteases functional domains and digital gene expression. Ubiquitin specific proteases (USP) digital gene expression analysis between slow and fast skeletal muscle. Analysis between fast and slow skeletal muscle was performed using the number of reads mapped normalized by length and library size. Normalized counts for individual animals per contig are shown. Differences between tissues were analysed by t-test followed by a False Discovery Rate correction. Significant differences were considered when FDR < 0.05. Pacu, zebrafish and human protein USP functional domains were determined using InterProScan webserver. Relative gene expression was analysed for fbox25, huwe, mafbx, murf1a, murf1b, syah1, syyna, trip12, ufd2, igf1, igf2a, igf2b, igf3, igf1ra, igf1rb, usp2a, usp2b, usp4, usp5a, usp5b, usp8, usp9, usp11, usp12b, usp12b, usp14, usp16, usp19, usp21, usp24, usp28, usp30, usp36 and usp46, Values represents mean ± SE (n = 8 fish). [file 12864_2015_1423_MOESM11_ESM.zip › Supplementary_file_11_USPs_domains/Graphs_gene_expression_E3-ubiquitin_USP_IGF/USP4.pdf]

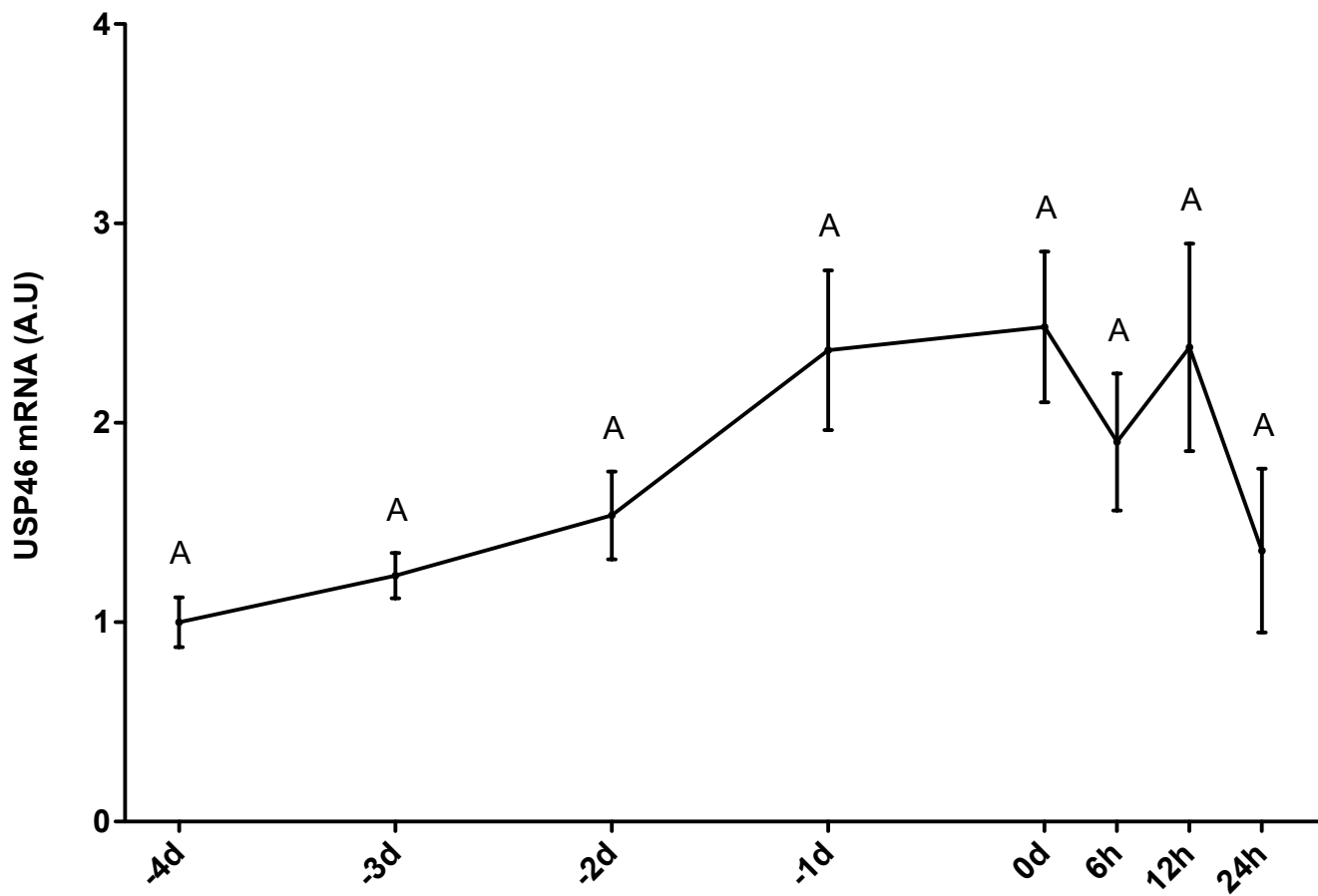

Supplement: Additional file 11: — Pacu ubiquitin specific proteases functional domains and digital gene expression. Ubiquitin specific proteases (USP) digital gene expression analysis between slow and fast skeletal muscle. Analysis between fast and slow skeletal muscle was performed using the number of reads mapped normalized by length and library size. Normalized counts for individual animals per contig are shown. Differences between tissues were analysed by t-test followed by a False Discovery Rate correction. Significant differences were considered when FDR < 0.05. Pacu, zebrafish and human protein USP functional domains were determined using InterProScan webserver. Relative gene expression was analysed for fbox25, huwe, mafbx, murf1a, murf1b, syah1, syyna, trip12, ufd2, igf1, igf2a, igf2b, igf3, igf1ra, igf1rb, usp2a, usp2b, usp4, usp5a, usp5b, usp8, usp9, usp11, usp12b, usp12b, usp14, usp16, usp19, usp21, usp24, usp28, usp30, usp36 and usp46, Values represents mean ± SE (n = 8 fish). [file 12864_2015_1423_MOESM11_ESM.zip › Supplementary_file_11_USPs_domains/Graphs_gene_expression_E3-ubiquitin_USP_IGF/USP46.pdf]

USP5a mRNA (A.U)

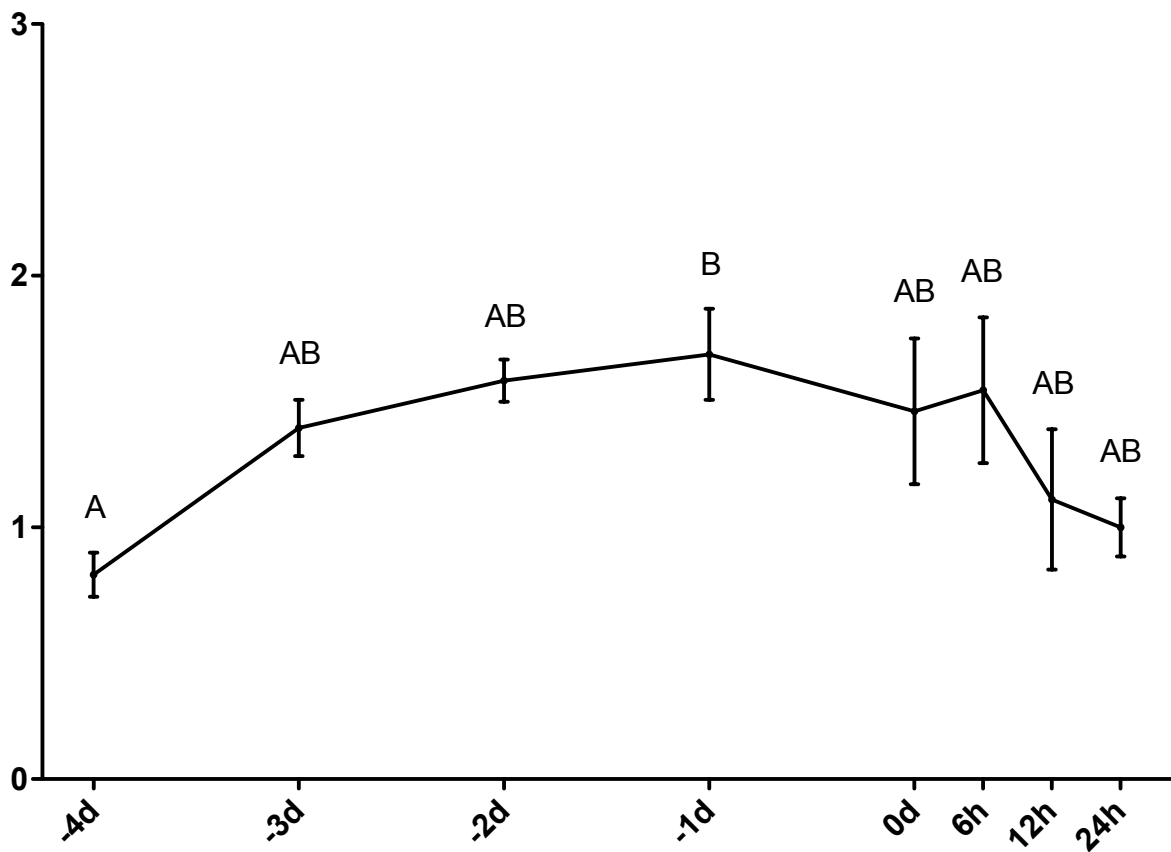

Supplement: Additional file 11: — Pacu ubiquitin specific proteases functional domains and digital gene expression. Ubiquitin specific proteases (USP) digital gene expression analysis between slow and fast skeletal muscle. Analysis between fast and slow skeletal muscle was performed using the number of reads mapped normalized by length and library size. Normalized counts for individual animals per contig are shown. Differences between tissues were analysed by t-test followed by a False Discovery Rate correction. Significant differences were considered when FDR < 0.05. Pacu, zebrafish and human protein USP functional domains were determined using InterProScan webserver. Relative gene expression was analysed for fbox25, huwe, mafbx, murf1a, murf1b, syah1, syyna, trip12, ufd2, igf1, igf2a, igf2b, igf3, igf1ra, igf1rb, usp2a, usp2b, usp4, usp5a, usp5b, usp8, usp9, usp11, usp12b, usp12b, usp14, usp16, usp19, usp21, usp24, usp28, usp30, usp36 and usp46, Values represents mean ± SE (n = 8 fish). [file 12864_2015_1423_MOESM11_ESM.zip › Supplementary_file_11_USPs_domains/Graphs_gene_expression_E3-ubiquitin_USP_IGF/USP5a.pdf]

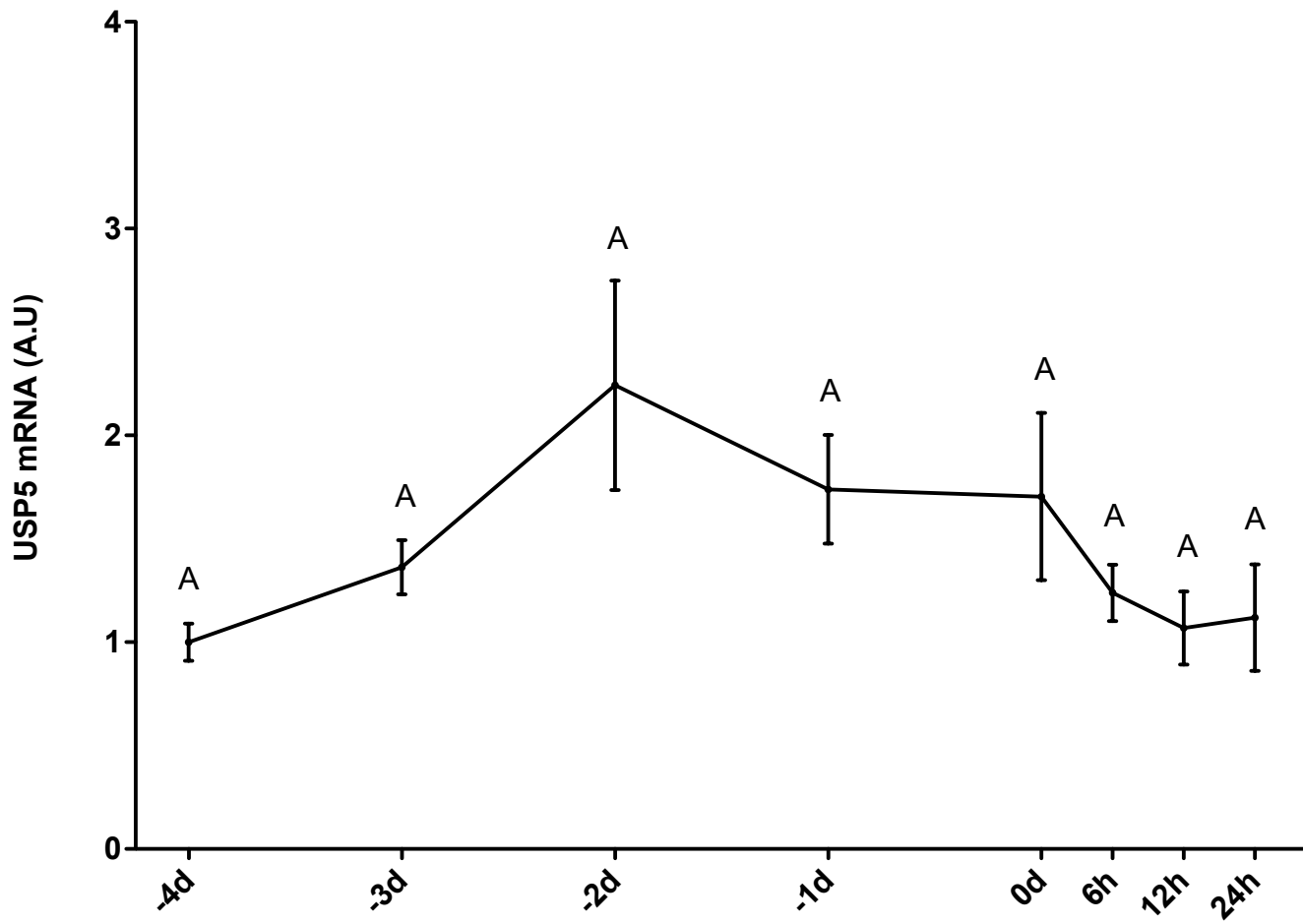

Supplement: Additional file 11: — Pacu ubiquitin specific proteases functional domains and digital gene expression. Ubiquitin specific proteases (USP) digital gene expression analysis between slow and fast skeletal muscle. Analysis between fast and slow skeletal muscle was performed using the number of reads mapped normalized by length and library size. Normalized counts for individual animals per contig are shown. Differences between tissues were analysed by t-test followed by a False Discovery Rate correction. Significant differences were considered when FDR < 0.05. Pacu, zebrafish and human protein USP functional domains were determined using InterProScan webserver. Relative gene expression was analysed for fbox25, huwe, mafbx, murf1a, murf1b, syah1, syyna, trip12, ufd2, igf1, igf2a, igf2b, igf3, igf1ra, igf1rb, usp2a, usp2b, usp4, usp5a, usp5b, usp8, usp9, usp11, usp12b, usp12b, usp14, usp16, usp19, usp21, usp24, usp28, usp30, usp36 and usp46, Values represents mean ± SE (n = 8 fish). [file 12864_2015_1423_MOESM11_ESM.zip › Supplementary_file_11_USPs_domains/Graphs_gene_expression_E3-ubiquitin_USP_IGF/USP5b.pdf]

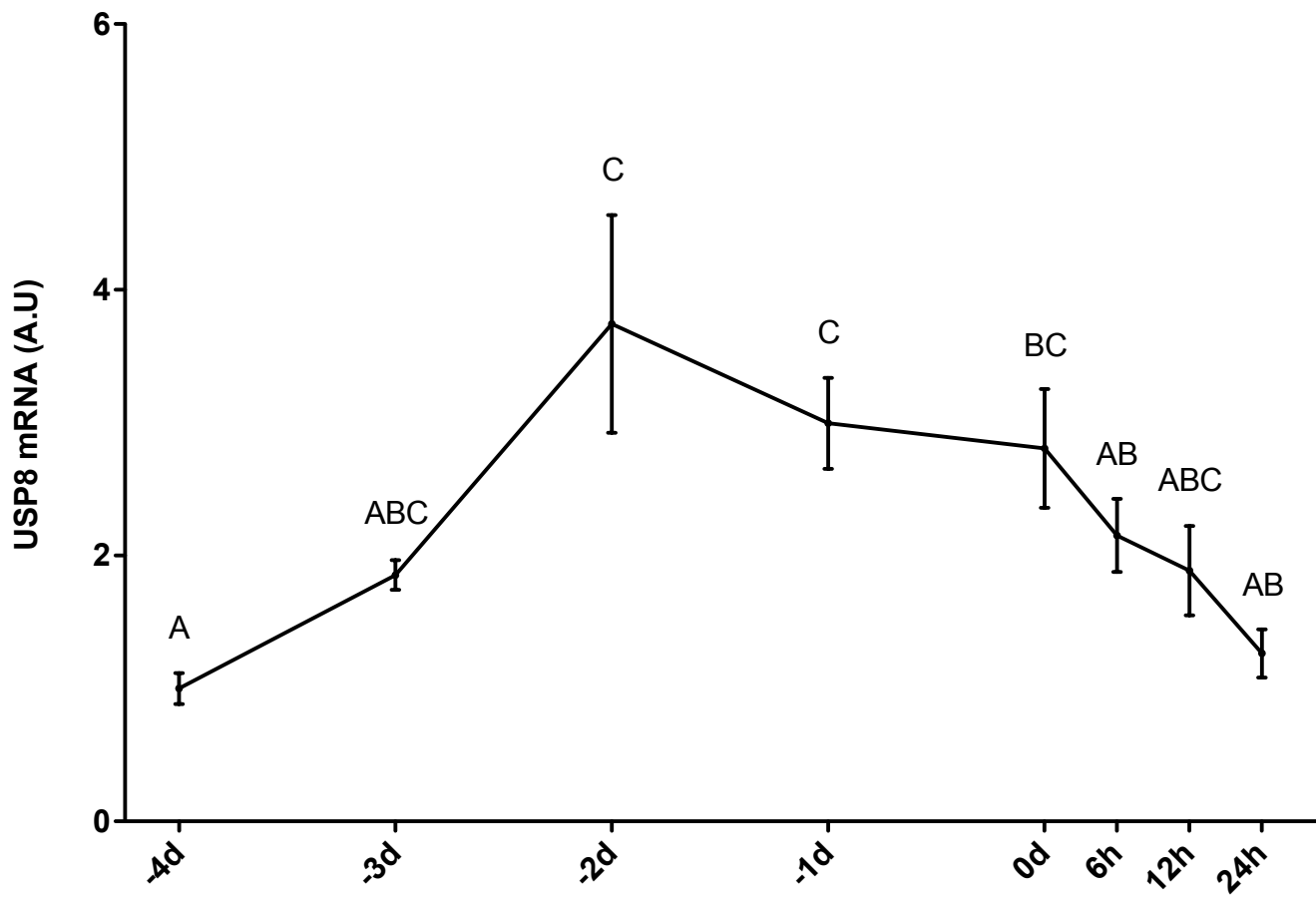

Supplement: Additional file 11: — Pacu ubiquitin specific proteases functional domains and digital gene expression. Ubiquitin specific proteases (USP) digital gene expression analysis between slow and fast skeletal muscle. Analysis between fast and slow skeletal muscle was performed using the number of reads mapped normalized by length and library size. Normalized counts for individual animals per contig are shown. Differences between tissues were analysed by t-test followed by a False Discovery Rate correction. Significant differences were considered when FDR < 0.05. Pacu, zebrafish and human protein USP functional domains were determined using InterProScan webserver. Relative gene expression was analysed for fbox25, huwe, mafbx, murf1a, murf1b, syah1, syyna, trip12, ufd2, igf1, igf2a, igf2b, igf3, igf1ra, igf1rb, usp2a, usp2b, usp4, usp5a, usp5b, usp8, usp9, usp11, usp12b, usp12b, usp14, usp16, usp19, usp21, usp24, usp28, usp30, usp36 and usp46, Values represents mean ± SE (n = 8 fish). [file 12864_2015_1423_MOESM11_ESM.zip › Supplementary_file_11_USPs_domains/Graphs_gene_expression_E3-ubiquitin_USP_IGF/USP8.pdf]

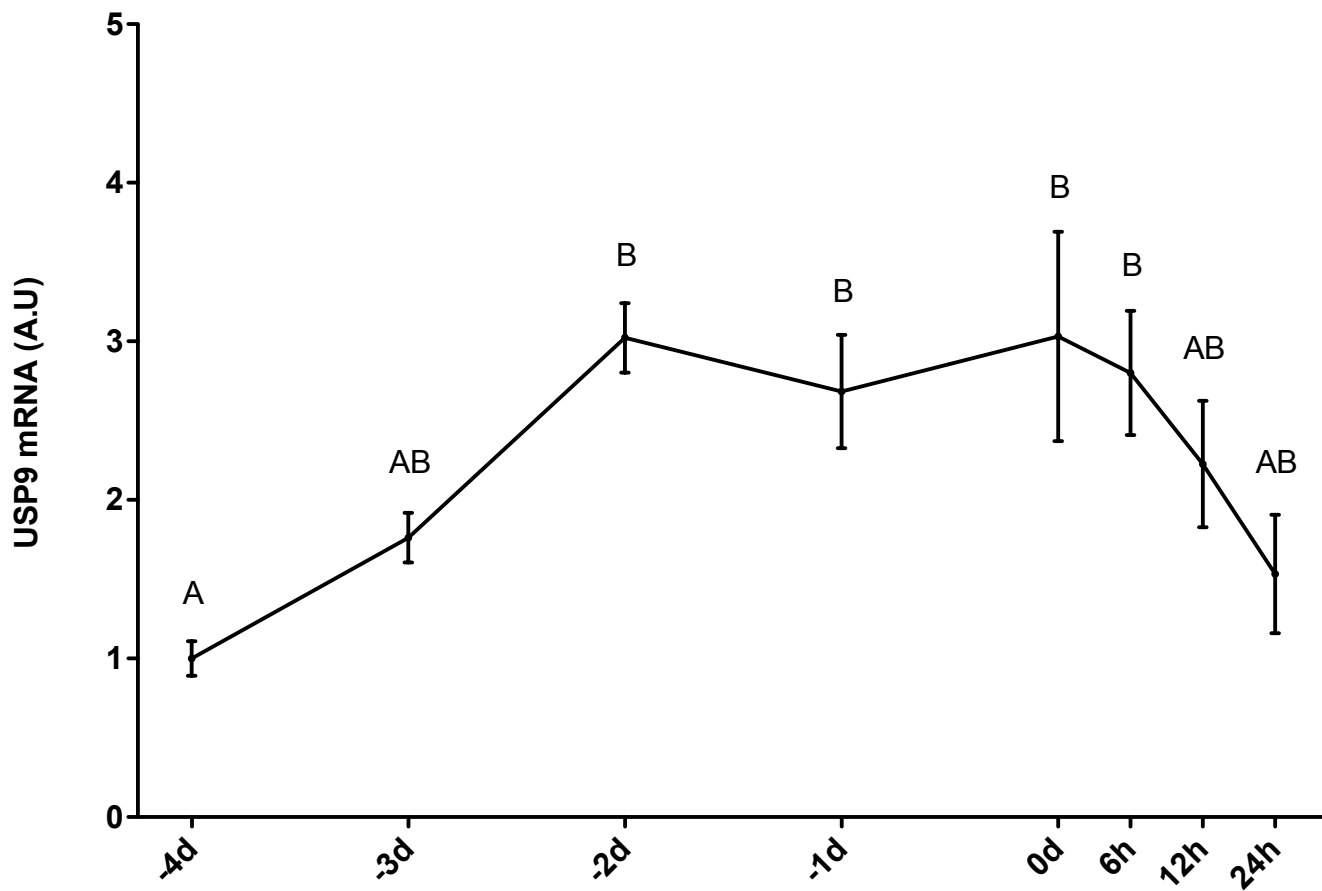

Supplement: Additional file 11: — Pacu ubiquitin specific proteases functional domains and digital gene expression. Ubiquitin specific proteases (USP) digital gene expression analysis between slow and fast skeletal muscle. Analysis between fast and slow skeletal muscle was performed using the number of reads mapped normalized by length and library size. Normalized counts for individual animals per contig are shown. Differences between tissues were analysed by t-test followed by a False Discovery Rate correction. Significant differences were considered when FDR < 0.05. Pacu, zebrafish and human protein USP functional domains were determined using InterProScan webserver. Relative gene expression was analysed for fbox25, huwe, mafbx, murf1a, murf1b, syah1, syyna, trip12, ufd2, igf1, igf2a, igf2b, igf3, igf1ra, igf1rb, usp2a, usp2b, usp4, usp5a, usp5b, usp8, usp9, usp11, usp12b, usp12b, usp14, usp16, usp19, usp21, usp24, usp28, usp30, usp36 and usp46, Values represents mean ± SE (n = 8 fish). [file 12864_2015_1423_MOESM11_ESM.zip › Supplementary_file_11_USPs_domains/Graphs_gene_expression_E3-ubiquitin_USP_IGF/USP9.pdf]
