# Supplementary material for: Characterization of the transcriptome of fast and slow muscle myotomal fibres in the pacu (Piaractus mesopotamicus)
Source: BMC Genomics. 2015 Mar 14;16(1):182. doi: 10.1186/s12864-015-1423-6 (PMC4372171; doi:10.1186/s12864-015-1423-6)
Supplement: Additional file 11: — Pacu ubiquitin specific proteases functional domains and digital gene expression. Ubiquitin specific proteases (USP) digital gene expression analysis between slow and fast skeletal muscle. Analysis between fast and slow skeletal muscle was performed using the number of reads mapped normalized by length and library size. Normalized counts for individual animals per contig are shown. Differences between tissues were analysed by t-test followed by a False Discovery Rate correction. Significant differences were considered when FDR < 0.05. Pacu, zebrafish and human protein USP functional domains were determined using InterProScan webserver. Relative gene expression was analysed for fbox25, huwe, mafbx, murf1a, murf1b, syah1, syyna, trip12, ufd2, igf1, igf2a, igf2b, igf3, igf1ra, igf1rb, usp2a, usp2b, usp4, usp5a, usp5b, usp8, usp9, usp11, usp12b, usp12b, usp14, usp16, usp19, usp21, usp24, usp28, usp30, usp36 and usp46, Values represents mean ± SE (n = 8 fish). [file 12864_2015_1423_MOESM11_ESM.zip › Supplementary_file_11_USPs_domains/USP_Domains.docx]

|  | Pacu | | | | | Zebrafish | | | | | | Human | | | | |
| --- | --- | --- | --- | --- | --- | --- | --- | --- | --- | --- | --- | --- | --- | --- | --- | --- |
|  | Length | Domains | CDS (%) | SignalP | PEST | Length | Domains | ID  (%) | | SignalP | PEST | Length | Domains | ID  (%) | SignalP | PEST |
| USP1 | 802 | UCH_2_3  (81-798) | 100 | NO | 34-73  128-143  457-486  615-628 | 772 | UCH_2_3  (81-771) |  | | NO | 35-60  436-465 | 785 | UCH_2_3  (81-775) |  | NO | 305-316  567-469 |
| USP2b | 395 | UCH_2_3  (62-394) | 100 | NO | NO | 395 | UCH_2_3  (59-391) |  | | NO | NO | 396 | UCH_2_3  (58-391) |  | NO | NO |
| USP2a |  |  |  |  |  | 600 | UCH_2_3  (268-600) |  | | NO | NO | 605 | UCH_2_3  (267-600) |  | NO | NO |
| USP4 | 742 | UCH_2_3  (320-742)  DUSP  (27-138) | 73 | NO | 1-33  727-742 | 1009 | UCH_2_3  (319-982)  DUSP  (27-138) |  | | NO | 1-18  735-757  988-1005 | 963 | UCH_2_3  (302-924)  DUSP  (11-122) |  | NO | 671-695  929-944 |
| USP5b | 859 | UCH_2_3  (318-857)  ZF-UBP  (190-262)  UBA  (634-675/709-759)  EF-HAND  (623-635) | 100 | NO | 675-725  749-778  778-793 | 860 | UCH_2_3  (318-858)  ZF-UBP  (190-262)  UBA  (635-676/710-760)  EF-HAND  (624-636) |  | | NO | 676-726  750-779  779-794 |  |  |  |  |  |
| USP5a | 834 | UCH_2_3  (324-833)  ZF-UBP  (195-267)  UBA  (632-673/699-739) | 97 | NO | 80-93  389-405  508-523  673-704 | 853 | UCH_2_3  (324-852)  ZF-UBP  (195-267)  UBA  (651-692/718-768) |  | | NO | 80-93  389-405  508-523  692-723 | 858 | UCH_2_3  (326-857)  ZF-UBP  (197-269)  UBA  (654-695/722-762) |  | NO | 80-93  603-628 |
| USP7 | 946 | UCH_2_3  (215-530)  MATH  (69-196) | 85 | NO | 12-36  778-792 | 1103 | UCH_2_3  (215-523)  MATH  (69-196) |  | | NO | 12-36  771-785 | 1102 | UCH_2_3  (214-522)  MATH  (68-195) |  | NO | 11-35  770-784 |
| USP8 | 508 | RHODANESE  (196-318)  DUF1873  (6-113) | 48 | NO | NO | 1067 | UCH_2_3  (735-1067)  RHODANESE  (188-310)  DUF1873  (6-113) |  | |  | 356-375  641-655 | 1118 | UCH_2_3  (777-1110)  RHODANESE  (195-313)  DUF1873  (6-113) |  | NO | 677-696 |
| USP9 | 2593 | UCH_2_3  (1559-1961) | 100 | NO | 6-71  967-993  1843-1864  2516-2541  2575-2592 | 2594 | UCH_2_3  (1556-1960) |  | | NO | 6-69  372-385  964-990  1840-1863  2575-2593 | 2554 | UCH_2_3  (1557-1957) |  | NO | 6-69  372-385  964-990  1834-1862  2488-2501  2535-2552 |
| USP10 | 874 | UCH_2_3  (488-872)  PAM2  (93-110) | 100 | NO | 246-291  313-374  374-408  454-468  646-662 | 865 | UCH_2_3  (481-863)  PAM2  (93-110) |  | | NO | 308-335  335-401  619-639  639-653 | 798 | UCH_2_3  (415-796)  PAM2  (78-95) |  | NO | 207-237  305-316  316-340  562-586 |
| USP11 | 491 | UCH_2_3  (148-479)  DUF1055  (1-115) | 49 | NO | NO | 990 | UCH_2_3  (242-949)  DUSP  (14-149)  GLU-RICH  (583-705) |  | NLS-BP  (714-728) | | 577-598  626-650  662-692  692-714 | 963/920 | UCH_2_3  (309-971)  DUSP  (76-184)  ALA-RICH  (2-20)  DUF1055  (114-275) |  | M/S | 645-657  677-693 |
| USP12a | 371 | UCH_2_3  (39-371) | 100 | NO | NO | 371 | UCH_2_3  (39-371) |  | | NO | NO | 370 | UCH_2_3  (39-370) |  | NO | NO |
| USP14 | 526 | UCH_2_3  (138-517)  UBIQU  (37-105) | 100 | M | 247-272  289-300 | 489 | UCH_2_3  (105-484)  UBIQU  (4-72) |  | | NO | 214-237  255-266 | 483 | UCH_2_3  (94-473) |  | NO | 213-227 |
| USP15 | 811 | UCH_2_3  (189-811)  DUF1055  (16-111) | 92 | NO | 535-599 | 879 | UCH_2_3  (258-866)  DUSP  (9-116)  DUF1055  (76-211) |  | | NO | 581-629 | 981 | UCH_2_3  (289-934)  DUSP  (7-118)  DUF1055  (78-212) |  | NO | 648-711  949-979 |
| USP16 | 701 | UCH_2_3  (76-701) | 86 | NO | 341-379  390-406  427-481 | 815 | UCH_2_3  (208-815)  ZF-UBP  (46-126) |  | | NO | 9-26  420-447  473-519  520-540  540-602 | 822 | UCH_2_3  (195-822)  ZF-UBP  (60-125) |  | NO | NO |
| USP19 | 1526 | UCH_2_3  (723-1453)  CS  (130-220/506-608)  ZF-MYND2  (1017-1059)  DUF1875  (18-72) | 100 | S | 46-68  248-261  1116-1174  1174-1192 | 1436 | UCH_2_3  (638-1363)  CS  (52-143/421-523)  ZF-MYND2  (932-974) |  | | NO | 369-382  400-422  1047-1083  1184-1200 | 1321 | UCH_2_3  (537-1255)  CS  (51-140/320-422)  ZF-MYND2  (831-873) |  | NO | 12-23  513-529 |
| USP20 | 304 | UCH_2_3  (148-304)  ZF-UBP  (28-92) | 33 | NO | 123-143  251-268  280-298 | 913 | UCH_2_3  (148-686)  ZF-UBP  (28-92)  DUSP  (687-782/791-893) |  | | NO | 123-143  280-298  360-386  837-849  890-901 | 914 | UCH_2_3  (145-686)  ZF-UBP  (28-92)  DUSP  (687-780/789-892) |  | NO | 106-117  120-140  276-292  338-370  835-847 |
| USP21 | 352 | UCH_2_3  (19-351) | 90 | NO | NO | 390 | UCH_2_3  (62-389) |  | | NO | NO | 565 | UCH_2_3  (212-559) |  | NO | 339-354  425-439 |
| USP22 | 494 | UCH_2_3  (147-490)  ZF-UBP  (32-92) | 100 | NLS-BP | NO | 466 | UCH_2_3  (119-462)  ZF-UBP  (4-64) |  | | NLS-BP  M | NO | 525 | UCH_2_3  (176-521)  ZF-UBP  (61-121) |  | NLS-BP  M | NO |
| USP24 | 2593 | UBA  (2-44)  UBIQUITIN-2  (932-1009)  UCH_2_3  (1665-2019)  SER-RICH  (995-1026) | 100 | NO | 1003-1039  1196-1208  2036-2059  2549-569 | 2589 | UBA  (3-45)  UBIQUITIN-2  (930-1007)  UCH_2_3  (1661-2015)  SER-RICH  (993-1024) |  | | NO | 1001-1037  1194-1206  2032-2055  2545-2565 | 2620 | UBA  (3-44)  UCH_2_3  (1689-2043)  SER-RICH  (1035-1062) |  | NO | 1033-1069  1220-1232  2060-2083  2576-2596 |
| USP25 | 687 | UCH_2_3  (1-248)  GLN-RICH  (12-104) | 64 | NO | 318-349 | 1072 | UIM  (96-115)  UCH_2_3  (168-660)  ALA-RICH  (719-733) |  | | NO | 751-766 | 1055 | UIM  (97-117)  UCH_2_3  (169-658) |  |  | 723-746  790-803 |
| USP28 | 731 | UCH_2_3  (1-175)  GLU-RICH  (216-404) | 63 | NO | 6-32  218-238  238-252  307-329  341-392 | 1163 | UCH_2_3  (150-631)  GLU-RICH  (672-836) |  | | NO | 442-488  685-772  772-842  895-911 | 1077 | UCH_2_3  (162-651)  GLU-RICH  (474-521) |  | NO | 64-85  455-468  486-511  702-729 |
| USP30 | 337 | UCH_2_3  (64-337) | 69 | NO | NO | 491 | UCH_2_3  (64-483) |  | | NO | NO | 486 | UCH_2_3  (37-472) |  | S | NO |
| USP31 | 1116 | UCH_2_3  (1-554)  SER-RICH  (558-1108) | 87 | NO | 857-873 | 1290 | UCH_2_3  (91-732)  SER-RICH  (736-1282) |  | | M | 343-371  1032-1044  1198-1219 | 1352 | UCH_2_3  (128-766)  SER-RICH  (770-1221)  PRO-RICH  (95-125) |  | NO | 165-178  1043-1065  1102-1114 |
| USP32 | 1422 | EF-HAND  (45-80/81-116/131-166)  DUSP  (186-403)  UCH_2_3  (548-386)  SER-RICH  (1169-1249)  DUF1055  (360-515) | 90 | NO | 1247-1305 | 1585 | EF-HAND  (91-126/228-263/264-299/314-349)  DUSP  (369-589)  UCH_2_3  (734-1549)  DUF1055  (546-701) |  | | NO | 459-481  1415-1469 | 1604 | EF-HAND  (91-126/228-263/264-299)  DUSP  (369-585)  UCH_2_3  (734-1568)  SER-RICH  (1359-1398)  DUF1055  (546-701) |  |  | 1323-1336 |
| USP33 | 896 | ZF-UBP  (29-93)  UCH_2_3  (156-670)  DUSP  (671-764/773-875) | 100 | NLS-BP | 130-151  259-283  285-304  819-831 | 897 | ZF-UBP  (29-93)  UCH_2_3  (156-671)  DUSP  (672-765/774-876) |  | | NLS-BP | 130-151  259-283 | 911 | ZF-UBP  (28-92)  UCH_2_3  (154-685)  DUSP  (686-779/787-890) |  | NO | 257-280  834-846 |
| USP34 | 840 | ASP-RICH  (620-651)  SER-RICH  (524-619)  HIS-RICH  (833-840) | >100 | NO | 101-116  116-134  148-174  522-538  561-593 | 582 | NO |  | | NO | 89-116  145-173  519-535 | 3546 | UCH_2_3  (1894-2240)  HIS-RICH  (749-763)  SER-RICH  (560-611) |  | NLS-BP | 141-152  509-530  553-585  1546-1481  2477-2508  3377-3391  3391-3413  3457-3484 |
| USP36 | 804 | UCH_2_3  (1-70)  IG-MHC  (22-28)  SER-RICH  (185-342) | 73 | NLS-BP | 277-289 | 1104 | UCH_2_3  (122-424)  IG-MHC  (376-382)  LYS-RICH  (749-815)  SER-RICH  (533-705) |  | | NO | 759-776 | 1123 | UCH_2_3  (122-424)  IG-MHC  (376-382)  PRO-RICH  (709-808) |  | NO | 666-687  708-720  796-811  936-954 |
| USP37 | 939 | UCH_2_3  (322-914)  UIM  (674-693/768-787) | 100 | NLS-BP  M | 619-638  696-727 | 935 | UCH_2_3  (322-910)  UIM  (764-783/786-805) |  | | NLS-BP  M | 433-446  619-638  793-835 | 979 | UCH_2_3  (341-952)  UIM  (704-723/802-825/828-847) |  | NLS-BP | 731-759  452-469 |
| USP38 | 1022 | UCH_2_3  (445-929) | 100 | NO | 577-588  749-780 | 1002 | UCH_2_3  (446-908)  SER-RICH  (763-854) |  | | NO | 762-787  843-863  553-565 | 1042 | UCH_2_3  (445-950) |  | NO | 825-836 |
| USP39 | 524 | ZF-UBP  (80-141)  UCH_2_3  (183-513) | 100 | NO | NO | 497 | ZF-UBP  (53-114)  UCH_2_3  (156-486) |  | | NO | NO | 536 | ZF-UBP  (122-183)  UCH_2_3  (225-536)  ARG-RICH  (4-103) |  | NO | NO |
| USP40 | 1281 | UCH_2_3  (42-528) | 100 | NO | NO | 1263 | UCH_2_3  (38-499) |  | | NO | 342-368  421-463 | 1235 | UCH_2_3  (41-483) |  | NO | NO |
| USP42 | 1173 | UCH_2_3  (113-414)  IG-MHC  (366-372)  ARG-RICH  (837-1073)  LYS-RICH  (1026-1065)  SER-RICH  (464-572) | 100 | NO | 516-576 | 1168 | UCH_2_3  (114-415)  IG-MHC  (367-373)  NMT2  (833-839)  ARG-RICH  (841-933)  LYS-RICH  (1007-1049)  SER-RICH  (497-567) |  | | NLS-BP | 30-50 | 1316 | UCH_2_3  (111-413)  IG-MHC  (365-371)  ARG-RICH  (946-1118)  LYS-RICH  (1176-1246)  PRO-RICH  (742-937) |  | NO | 474-492  598-620  635-649  649-670  746-773  787-836  911-923 |
| USP43a | 647 | UCH_2_3  (1-193) | 58 | NO | 235-254 | 1120 | UCH_2_3  (114-715) |  | | NO | 754-773 | 1123 | UCH_2_3  (101-711)  PRO-RICH  (41-98) |  | NO | NO |
| USP43b |  |  |  |  |  | 1183 | UCH_2_3  (116-763)  SER-RICH  (767-808) |  | | NO | 413-428  802-816 |  |  |  |  |  |
| USP44 | 690 | ZF-UBP  (24-85)  UCH_2_3  (290-680)  ARG-RICH  (133-217)  PRO-RICH  (241-287) | 99 | NO | NO | 695 | ZF-UBP  (24-85)  UCH_2_3  (295-685)  ARG-RICH  (149-264) |  | | NO | NO | 712 | ZF-UBP  (27-88)  UCH_2_3  (273-679)  ARG-RICH  (149-264) |  | NO | 698-712 |
| USP45 | 805 | ZF-UBP  (60-135)  UCH_2_3  (191-805)  SER-RICH  (476-550) | 100 | NO | 483-506 | 803 | ZF-UBP  (60-135)  UCH_2_3  (192-803)  SER-RICH  (409-542) |  | | NO | 494-507 | 814 | ZF-UBP  (60-136)  UCH_2_3  (190-814) |  |  | 481-492  496-515 |
| USP46 | 371 | UCH_2_3  (39-371) | 100 | NO | NO | 370 | UCH_2_3  (35-370) |  | | NO | NO | 366 | UCH_2_3  (35-366) |  | NO | NO |
| USP47 | 704 | UCH_2_3  (172-546) | >100 | NO | 109-136  411-430 | 357 | UCH_2_3  (170-357) |  | | NO | 107-134 | 1287 | UCH_2_3  (100-477) |  | NO | 339-358  810-827  841-855 |
| USP48 | 825 | UCH_2_3  (89-417)  DUSP  (457-551/567-695/715-825) | 78 | NO | NO | 1055 | UCH_2_3  (89-422)  DUSP  (463-557/573-705/725-838)  UBIQUITIN2  (969-1020) |  | | NO | 128-142  891-908 | 983 | UCH_2_3  (89-422)  DUSP  (460-554/569-691/711-824) |  | NO | 877-894 |
| USP49 | 681 | ZF-UBP  (24-85)  UCH_2_3  (288-680)  PRO-RICH  (215-258)  SER-RICH  (100-129) | 100 | NO | NO | 649 | ZF-UBP  (24-85)  UCH_2_3  (265-648) |  | | M | NO | 688 | ZF-UBP  (24-85)  UCH_2_3  (253-658)  ARG-RICH  (145-195) |  | NO | NO |
| USP53a | 1020 | UCH_2_3  (39-350) | 99 | M | 780-815 | 1029 | UCH_2_3  (50-363)  SER-RICH  (790-940) |  | | M | 788-813  918-931 | 1073 | UCH_2_3  (39-352) |  | NO | NO |
| USP53b | 1008 | UCH_2_3  (50-363) |  | NO | 783-808  808-821 |  |  |  | |  |  |  |  |  |  |  |
| USP54a | 583 | UCH_2_3  (33-354)  SER.RICH  (380-514) | 39 | M | NO | 1497 | UCH_2_3  (40-353)  SER-RICH  (378-437) |  | | NLS-BP  M | 435-452  1054-1087 | 1256 | UCH_2_3  (35-355)  SER-RICH  (374-513) |  | M | 988-1004 |
