# Supplementary material for: Characterization of the transcriptome of fast and slow muscle myotomal fibres in the pacu (Piaractus mesopotamicus)
Source: BMC Genomics. 2015 Mar 14;16(1):182. doi: 10.1186/s12864-015-1423-6 (PMC4372171; doi:10.1186/s12864-015-1423-6)
Supplement: Additional file 12: — qPCR primers sequence of target genes: fbox25, huwe, mafbx, murf1a, murf1b, syah1, syyna, trip12, ufd2, igf1, igf2a, igf2b, igf3, igf1ra, igf1rb, usp2a, usp2b, usp4, usp5a, usp5b, usp8, usp9, usp11, usp12b, usp12b, usp14, usp16, usp19, usp21, usp24, usp28, usp30, usp36 and usp46 and housekeeping genes: Ef1a, Polr2a, Gapdh, Hprt1, ppiaa, sdha, Rpl13, Rpl19. [file 12864_2015_1423_MOESM12_ESM.docx]

| Supplementary file 12 – qPCR primer sequences | | | | |
| --- | --- | --- | --- | --- |
| Gene | Sequence | | amplicon size | efficiency of reaction |
|  | Forward (5'-3') | Reverse (5'-3') |  |  |
| Mafbx | tctttggtgctccccttgtg | taaaaccgaggacggctgg | 231 | 1.90 |
| Fbox25 | cgtaggtgaagtagccgcca | ggagaagtccagcctgttga | 166 | 1.90 |
| Muf1a | ccattgatgccttggttgc | ccttctcgtcctgctctttg | 198 | 1.85 |
| Muf1b | ccccctgaaagacaaagaca | gctcagccttctggacttc | 179 | 1.89 |
| Siah1 | cacaaatccatcaccacgc | gccatcatacttttcctgc | 150 | 1.89 |
| Syvn1 | cgacctacagagcgagaacc | cagccttcttaaattgcctca | 185 | 1.88 |
| Trip12 | cgcagaagcagtcgtgataa | tgctgggggagactcactac | 222 | 1.87 |
| Huwe1 | gagacctaatcctccgccag | caccactgctctccacaatc | 228 | 1.87 |
| Ufd2 | agtcgtcacagggagatgct | ttgggaacctcaggaatcag | 206 | 1.89 |
| Usp2a | agcctgcgagactactgt | actcctgagcgtcctgttg | 214 | 1.89 |
| Usp2b | gcaccgacctcaacaacaac | acgaaacggggagcatactt | 151 | 1.94 |
| Usp4 | gccttcttgctggatggact | tcaggacagactagcgtgga | 194 | 1.92 |
| Usp5a | ccttcagcacttccagcaga | tgcgatttccaactccgtca | 199 | 1.90 |
| Usp5b | cccagaccactttgagattcc | atttcgcacttggcacactt | 222 | 1.94 |
| Usp8 | agagggcacacacttcaggt | gcgagagcagaggagagaga | 111 | 1.89 |
| Usp9 | cacaaacctcggaccaaaat | gcacagtcaaaaccctaacca | 179 | 1.88 |
| Usp11 | cactaacctcggcaacacct | agaaactgggacgcaaaatg | 249 | 1.90 |
| Usp12a | tcctaatgacagtttccaa | ggtgagcaggttctctttgc | 239 | 1.80 |
| Usp12b | atactgatgacagtccgaaag | aggtcagcaggctctctttg | 241 | 1.92 |
| Usp14 | ctctgaggaggtattcgggt | cagcaaactgtgggaaggc | 168 | 1.93 |
| Usp16 | ccgtcctcactctccacctg | ctgtgctccacgattccata | 181 | 1.89 |
| Usp19 | gccccacaagaaaccagtaa | gcacctcaaagcagaagagc | 215 | 1.89 |
| USP21 | tgtgctggatggagatgaga | cttctgtcggaggcaaactc | 210 | 1.81 |
| Usp22 | aactcctgcctcgtgtgtg | ttcttcccgttgtcgtcttt | 206 | 1.87 |
| Usp24 | aatcagaatcgtcggtggag | agtggcaaaagaggtgagga | 173 | 1.90 |
| Usp28 | acagtttgtgtcaggtcccc | ctcctcccacgtcgattct | 155 | 1.91 |
| Usp30 | ctctctgtccattcctgctgt | gtcgtcctctggctctcaac | 168 | 1.88 |
| Usp36 | gatgaagactggtgccttgga | ccttgggcggagagagtttt | 118 | 1.90 |
| Usp46 | agcggttccgttgttcttca | cttccacagcatcgccaca | 217 | 1.87 |
| Igf1ra | gcttctcaggctccgttcc | attcaccgtctaccgcatcg | 248 | 1.87 |
| Igf1rb | aggctggatgtgctctctt | gggtggctatcaaaacggt | 223 | 1.88 |
| Igf1 | atttcagcaagccaacaggt | cgcacaatacatctcaagtcg | 116 | 1.87 |
| Igf2a | cagcaagcagaagacaacga | gcagcattcctccactatcc | 217 | 1.90 |
| Igf2b | gttttgtcggttttggagga | gaaggtgctggaacaggaat | 199 | 1.91 |
| Igf3 | ggctcacactgaaggacaca | cgctctccacacacaaactc | 183 | 1.90 |
| Ef1a | attccactgagcccccttac | ttacgctcaaccttccatcc | 190 | 1.94 |
| Polr2a | atcaacccacgaactccaac | cctctgtgctaaggcgaaac | 205 | 1.92 |
| Gapdh | acacacgacgacaagaccaa | gtccctctcgctgaaaactg | 267 | 1.87 |
| Hprt1 | tgggctaaaggaaggatgag | tacaaagcgtgaggtggcta | 202 | 1.89 |
| ppiaa | attgtggttcgtgaagtcgc | ccgctgggcagagtgattat | 170 | 1.87 |
| sdha | acctgatgctgaatgctgtg | agtgtgcttcctccagtgc | 170 | 1.90 |
| Rpl13 | atcaacaggaaagtagccc | aggatgagtttggagcggta | 122 | 1.89 |
| Rpl19 | gcaaactggtgaaggatggt | cttggactccctgtaacgcc | 215 | 1.85 |
